# Transcriptomic analysis of tuberous root in two sweet potato varieties reveals the important genes and regulatory pathways in tuberous root development

**DOI:** 10.1186/s12864-022-08670-x

**Published:** 2022-06-27

**Authors:** Zhaoqin Cai, Zhipeng Cai, Jingli Huang, Aiqin Wang, Aaron Ntambiyukuri, Bimei Chen, Ganghui Zheng, Huifeng Li, Yongmei Huang, Jie Zhan, Dong Xiao, Longfei He

**Affiliations:** 1 National Demonstration Center for Experimental Plant Science Education, College of Agriculture, Guangxi University, Nanning, 530004 People’s Republic of China; 2 Guangxi South Subtropical Agricultural Science Research Institute, Chongzuo, 532406 People’s Republic of China; 3 Guangxi Colleges and Universities Key Laboratory of Crop Cultivation and Tillage, Nanning, 530004 People’s Republic of China; 4 Hepu Institute of Agricultural Sciences, Beihai, 536101 People’s Republic of China; 5 Maize Research Institute of Guangxi Academy of Agricultural Sciences, Nanning, 530007 People’s Republic of China

**Keywords:** Tuberous root, Transcriptomic analysis, Sweet potato, Development, Core genes

## Abstract

**Background:**

Tuberous root formation and development is a complex process in sweet potato, which is regulated by multiple genes and environmental factors. However, the regulatory mechanism of tuberous root development is unclear.

**Results:**

In this study, the transcriptome of fibrous roots (R0) and tuberous roots in three developmental stages (Rl, R2, R3) were analyzed in two sweet potato varieties, GJS-8 and XGH. A total of 22,914 and 24,446 differentially expressed genes (DEGs) were identified in GJS-8 and XGH respectively, 15,920 differential genes were shared by GJS-8 and XGH. KEGG pathway enrichment analysis showed that the DEGs shared by GJS-8 and XGH were mainly involved in “plant hormone signal transduction” “starch and sucrose metabolism” and “MAPK signal transduction”. Trihelix transcription factor (Tai6.25300) was found to be closely related to tuberous root enlargement by the comprehensive analysis of these DEGs and weighted gene co-expression network analysis (WGCNA).

**Conclusion:**

A hypothetical model of genetic regulatory network for tuberous root development of sweet potato is proposed, which emphasizes that some specific signal transduction pathways like “plant hormone signal transduction” “Ca^2+^signal” “MAPK signal transduction” and metabolic processes including “starch and sucrose metabolism” and “cell cycle and cell wall metabolism” are related to tuberous root development in sweet potato. These results provide new insights into the molecular mechanism of tuberous root development in sweet potato.

**Supplementary Information:**

The online version contains supplementary material available at 10.1186/s12864-022-08670-x.

## Introduction

Sweet potato (*Ipomoea batatas* L) is a dicotyledonous plant of the family Convolvulaceae, growing in tropical, subtropical, and temperate regions, it is the most important rhizome crop after potato and cassava, and one of the most important food crops in the world [1], with an annual global output of more than 100 million tons. China is the largest sweet potato producer in the world, accounting for 80–85% of the global output [1]. Sweet potato is nutritious and contains many ingredients for human health, which has the medicinal values such as anti-cancer, anti-diabetes and anti-inflammatory activity [2, 3], and has been selected as one of the test foods for long-term space travel [4]. The tuberous root of sweet potato is rich in starch and soluble sugar, and its biomass is the highest in all crops. Sweet potato is listed as the key raw material for ethanol production because of its high starch content [5]. How to improve the yield and quality of sweet potato has become a top priority.

Endogenous hormones play an important role in the process of tuberous root expansion. Cytokinin (CTK) and abscisic acid (ABA) are involved in the formation of stored roots [6–11], t-zeatin is thought to play an important role in the induction of tuberous roots by activating the primary cambium. ABA regulates the thickening of tuberous roots by activating the cell division of meristem. The content of Auxin (IAA) increased gradually at the initial stage of root expansion in sweet potato tuberous root, and began to decrease after the beginning of secondary growth, while the content of ABA and cytokinin was steadily increased [12, 13]. In tuberous root, the content of jasmonic acid (JA) was very high, while the contents in burdock root and fibrous root were less [14].

The growth and expansion of tuberous root in sweet potato are genetically regulated. Previous studies have shown that MADS-box, KNOX genes were highly expressed and related to the expansion of tuberous root in sweet potato [15–17]. The overexpression of *SRD1* gene promoted the proliferation of cambium cells and xylem cells, and played a role in auxin-mediated initial root thickening [12]. SRF6 was the most abundantly expressed in tuberous root, and its mRNA was located around the primary cambium and meristem of the xylem, promoting the thickening of the tuberous root [18, 19]. Besides, an expansin coding gene *IbEXP1* was found to play an inhibitory role in the proliferation of cambium cells and xylem cells, which in turn inhibited the initial expansion of tuberous root in sweet potato [19]. The tuberous root development of sweet potato is regulated by multiple genes. However, few genes related to tuberous root development have been identified, and no specific genes regulating tuberous root development of sweet potato have been found, so more researches are needed to reveal the molecular mechanism of tuberous root development of sweet potato.

With the rapid development of sequencing and molecular technology, the study on the molecular mechanism of underlying tuberous root expansion in sweet potato has made great progress. However, the development of tuberous root in sweet potato is a complex biological process, and its mechanism is not clear. Sweet potato is a heterohexaploid plant (2n = 6x = 90) with a genome of 4.4 GB [20]. There are some studies on the development mechanism of sweet potato tuberous root at the transcriptional level. It was found that some specific genes and proteins associated with starch and phytohormone synthesis as well as various transcription factors are involved in storage root formation and development [17, 21–23], but there are many genes should be found at transcriptional level. In the meanwhile, previous studies were based on a single variety, however, there are great genetic differences among varieties. It is difficult to explain the general mechanism and variety specificity from transcriptomic analysis using a single variety. In this study, two main sweet potato cultivars with similar developmental processes but having great genetic differences and usually planted in Guangxi Zhuang Autonomous Region of PR China, Xiguahong (XGH, orange flesh sweet potato) and Guijingshu 8 (GJS-8, purple flesh sweet potato), were used as plant materials. RNA sequencing and weighted gene co-expression network analysis (WGCNA) were performed to identify the key candidate genes mediating tuberous root development.

## Results

### Identification of differentially expressed genes between fibrous root and tuberous root

**Fig. 1:**
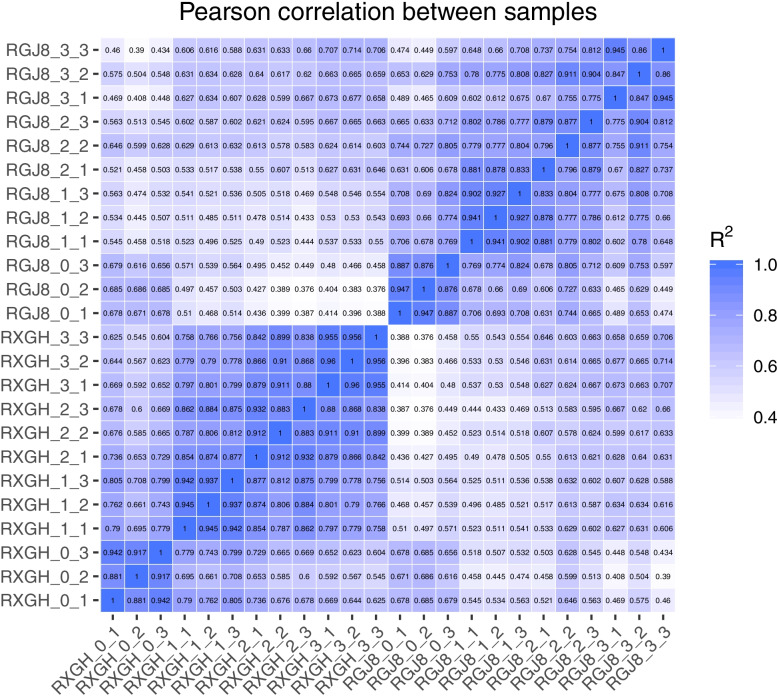
Correlation analysis between sample replicates

To explore the molecular mechanism of the formation and development of tuberous roots of sweet potato, 8 cDNA libraries were generated from the fibrous roots(R0) and the tuberous roots at different development stages (R1, R2, R3) in GJS-8 and XGH. Based on Illumina sequencing, a total of 1,514,457,568 original readings were obtained. After removing the connectors, unknown bases and low-quality reads, 1,486,623,198 clean readings were obtained, with an error rate of less than 0.03, Q20 > 97%, Q30 > 93%, which met the quality requirements of database construction. These clean readings were compared to the sweet potato genome using HISAT2 platform, and each library compared the number of reads on the genome to more than 69%. The number of reads aligned to the unique location of the reference genome was more than 63%, and the number of reads aligned to multiple locations of the reference genome was about 3.2–3.8% (Table 1). The sample correlation heat map showed that the R2 value among three biological repetitive samples was greater than 0.8, and that of most of samples was greater than 0.9, indicating that this experiment was highly repeatable and the data were reliable (Fig. 1).

**Table 1:** Quality statistics of original sequencing data and alignment analysis of filtered data with reference genome sequence

Sample	Raw_reads	Clean_reads	Clean_bases	Q20	Q30	Total_map	Unique_map	Multi_map
RGJ8_0_1	54,855,784	53,608,048	8.04G	97.83	93.58	35,656,422(66.51%)	33,895,916(63.23%)	1,760,506(3.28%)
RGJ8_0_2	50,754,086	49,391,586	7.41G	98.02	93.98	33,894,841(68.62%)	32,215,839(65.23%)	1,679,002(3.4%)
RGJ8_0_3	60,342,940	58,716,810	8.81G	97.92	93.76	41,968,662(71.48%)	39,919,527(67.99%)	2,049,135(3.49%)
RGJ8_1_1	55,763,332	54,758,234	8.21G	97.84	93.58	41,532,205(75.85%)	39,473,934(72.09%)	2,058,271(3.76%)
RGJ8_1_2	54,963,332	53,697,528	8.05G	97.98	93.92	40,923,281(76.21%)	39,087,326(72.79%)	1,835,955(3.42%)
RGJ8_1_3	53,018,150	52,143,466	7.82G	98.06	94.11	38,338,903(73.53%)	36,568,391(70.13%)	1,770,512(3.4%)
RGJ8_2_1	62,021,164	60,915,180	9.14G	97.74	93.36	47,688,641(78.29%)	45,463,907(74.63%)	2,224,734(3.65%)
RGJ8_2_2	65,214,940	64,099,854	9.61G	98.07	94.11	47,955,582(74.81%)	45,694,127(71.29%)	2,261,455(3.53%)
RGJ8_2_3	63,298,644	62,034,718	9.31G	97.9	93.72	47,573,504(76.69%)	45,225,371(72.9%)	2,348,133(3.79%)
RGJ8_3_1	69,201,124	67,485,452	10.12G	97.84	93.58	50,006,430(74.1%)	47,384,881(70.21%)	2,621,549(3.88%)
RGJ8_3_2	77,918,304	76,533,928	11.48G	97.96	93.82	57,958,641(75.73%)	54,988,545(71.85%)	2,970,096(3.88%)
RGJ8_3_3	68,399,538	67,593,522	10.14G	97.91	93.77	51,855,415(76.72%)	49,265,248(72.88%)	2,590,167(3.83%)
RXGH_0_1	60,470,236	59,395,634	8.91G	97.77	93.47	42,773,878(72.02%)	40,808,492(68.71%)	1,965,386(3.31%)
RXGH_0_2	64,126,042	63,252,848	9.49G	98.08	94.18	46,675,900(73.79%)	44,499,900(70.35%)	2,176,000(3.44%)
RXGH_0_3	58,523,750	57,486,536	8.62G	97.82	93.59	42,259,753(73.51%)	40,308,434(70.12%)	1,951,319(3.39%)
RXGH_1_1	59,230,394	58,084,228	8.71G	97.92	93.82	41,855,180(72.06%)	40,072,574(68.99%)	1,782,606(3.07%)
RXGH_1_2	62,660,420	61,717,000	9.26G	97.79	93.49	45,089,703(73.06%)	43,198,530(69.99%)	1,891,173(3.06%)
RXGH_1_3	62,732,322	61,817,364	9.27G	97.78	93.45	46,399,696(75.06%)	44,450,491(71.91%)	1,949,205(3.15%)
RXGH_2_1	74,887,522	73,620,460	11.04G	97.74	93.36	56,299,859(76.47%)	53,684,052(72.92%)	2,615,807(3.55%)
RXGH_2_2	86,367,676	84,896,874	12.73G	97.66	93.14	66,256,302(78.04%)	63,046,641(74.26%)	3,209,661(3.78%)
RXGH_2_3	68,133,460	67,070,154	10.06G	98.34	94.79	51,470,162(76.74%)	49,089,797(73.19%)	2,380,365(3.55%)
RXGH_3_1	59,273,332	58,226,124	8.73G	97.77	93.46	45,695,896(78.48%)	43,444,717(74.61%)	2,251,179(3.87%)
RXGH_3_2	58,250,960	57,096,602	8.56G	97.81	93.5	44,842,303(78.54%)	42,632,664(74.67%)	2,209,639(3.87%)
RXGH_3_3	64,050,116	62,981,048	9.45G	97.91	93.77	49,630,747(78.8%)	47,242,197(75.01%)	2,388,550(3.79%)

**Fig. 2:**
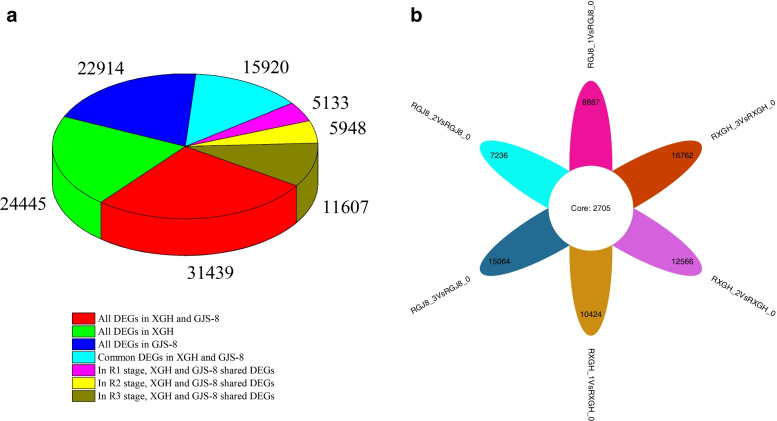
The number of differentially expressed genes between tuberous root and fiber root at different stages of GJS-8 and XGH. **a** Statistics on the number of differential genes in different situations. **b** The number of differential genes between GJS-8 and XGH

The expression levels of genes were measured and analyzed. Taking | log2 (FoldChange) | > 1 and padj < 0.05 as the standard, we identified 31,440 differentially expressed genes (DEGs) for the tuberous roots (R1, R2 and R3) vs. fibrous root in GJS-8 and XGH, of which 22,914 were in GJS-8, and 24,446 DEGs in XGH. GJS-8 and XGH shared 15,920 DEGs, of which 5133 DEGs in R1 stage, 5948 in R2 stage, and 11,607 in R3 stage (Fig. 2A). In addition, there were 2705 common genes involved in the whole tuberous root development process in GJS-8 and XGH (Fig. 2B).

### GO and KEGG enrichment analysis of DEGs

To further determine the main biological functions of all DEGs shared by GJS-8 and XGH in the process of tuberous root development, functional annotation was performed by mapping all common DEGs to gene ontology (GO) terms in the GO database. GO enrichment analysis was implemented using a Bonferroni-corrected *p* ≤ 0.05 as the threshold. Based on this criterion, 33 biological process terms, 3 cellular component terms and 36 molecular function terms were significantly enriched in R1 vs. R0 comparison. Among the DEGs between R1 vs. R0, the “cellular carbohydrate metabolic process” and “single-organism carbohydrate metabolic process” were the major terms of biological process, the “cell wall” and “external encapsulating structure” were the major terms of cellular component, and the “nucleic acid binding transcription factor activity” was the most represented molecular function term (Table S1). A total of 91 biological process terms, 7 cellular component terms and 49 molecular function terms were significantly enriched in R2 vs. R0 comparison. Among the DEGs between R2 vs. R0, the “response to stress” and “single-organism carbohydrate metabolic process” were the major terms of biological process, the “cell periphery”, “cell wall” and “external encapsulating structure” were the major terms of cellular component, and the “nucleic acid binding transcription factor activity” was the most represented molecular function term (Table S2). Moreover, 75 biological process terms, 6 cellular component terms, and 39 molecular function terms were significantly enriched in R3 vs. R0 comparisons. Among the DEGs between R3 vs. R0, the “ion transport” and “cell communication” were the major terms of biological process, “cell periphery” “cell wall” and “external encapsulating structure” were major terms of cellular component, and “nucleic acid binding transcription factor activity” was the most represented molecular function term (Table S3).

**Fig. 3:**
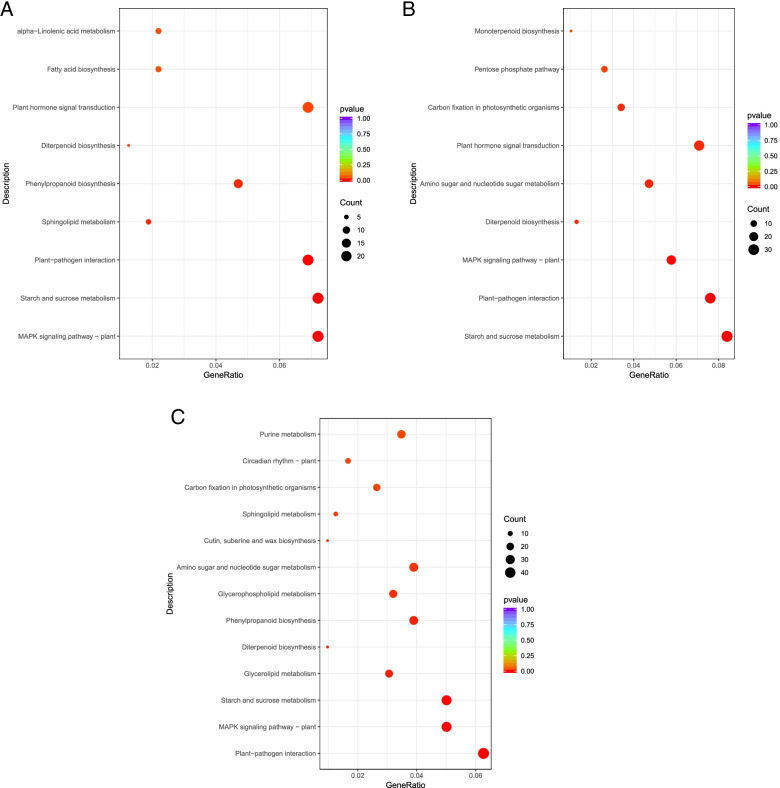
KEGG enrichment analysis of DEGs shared by GJS-8 and XGH at R1, R2, R3 stages. **a** DEGs shared by GJS-8 and XGH at R1 stage; **b** DEGs shared by GJS-8 and XGH at R2 stage; **c** DEGs shared by GJS-8 and XGH at R3 stage

To further determine the metabolic or signal transduction pathways that common DEGs may participate in tuberous root development, pathway enrichment analysis was performed by using KEGG database. A total of 5133 (R1 vs. R0), 5948 (R2 vs. R0), and 11,607 (R3 vs. R0) DEGs were respectively assigned to 101, 105, and 110 pathways by KEGG pathway enrichment analysis. Nine pathways were identified as significantly enriched pathways in R1 vs. R0 and R2 vs. R0, respectively, and 13 were identified as significantly enriched pathways in R3 vs. R0 (Q ≤ 0.05) (Table 2; Fig. 3). The “Starch and sucrose metabolism (sot00500)” “MAPK signaling pathway - plant (sot04016)” “plant hormone signal transductiont (sot04075)” and “plant-pathogen interaction (sot04626)” were the major represented pathways among the DEGs of R1 vs. R0 and R2 vs. R0. Among the DEGs between R3 vs. R0, the “Starch and sucrose metabolism (sot00500)” “MAPK signaling pathway - plant (sot04016)” “Circadian rhythm - plant (sot04712)” and “Plant-pathogen interaction (sot04626)” were the major represented pathways. The results suggest that genes involved in regulation of plant hormone levels, metabolism and signal transduction played vital roles in tuberous root of sweet potato.

**Table 2:** KEGG enrichment analysis of common differential genes in different stages of GJS-8 and XGH

KEGGID	Term	p-value	Gene Number	
sot04016	MAPK signaling pathway - plant	1.40436E-05	23	R_1_ Vs R_0_
sot00500	Starch and sucrose metabolism	3.30079E-05	23	
sot04626	Plant-pathogen interaction	0.000156386	22	
sot00600	Sphingolipid metabolism	0.011080663	6	
sot00940	Phenylpropanoid biosynthesis	0.012234384	15	
sot00904	Diterpenoid biosynthesis	0.024164376	4	
sot04075	Plant hormone signal transduction	0.028135623	22	
sot00061	Fatty acid biosynthesis	0.038762762	7	
sot00592	alpha-Linolenic acid metabolism	0.038762762	7	
sot00500	Starch and sucrose metabolism	1.35885E-08	32	R_2_ Vs R_0_
sot04626	Plant-pathogen interaction	1.35835E-06	29	
sot04016	MAPK signaling pathway - plant	0.000622851	22	
sot00904	Diterpenoid biosynthesis	0.008765197	5	
sot00520	Amino sugar and nucleotide sugar metabolism	0.010132352	18	
sot04075	Plant hormone signal transduction	0.010903657	27	
sot00710	Carbon fixation in photosynthetic organisms	0.011375788	13	
sot00030	Pentose phosphate pathway	0.02528945	10	
sot00902	Monoterpenoid biosynthesis	0.032595474	4	
sot04626	Plant-pathogen interaction	1.658E-07	45	R_3_ Vs R_0_
sot04016	MAPK signaling pathway - plant	0.000129123	36	
sot00500	Starch and sucrose metabolism	0.000398031	36	
sot00561	Glycerolipid metabolism	0.007070632	22	
sot00904	Diterpenoid biosynthesis	0.007556666	7	
sot00940	Phenylpropanoid biosynthesis	0.008022448	28	
sot00564	Glycerophospholipid metabolism	0.015204748	23	
sot00520	Amino sugar and nucleotide sugar metabolism	0.017481772	28	
sot00073	Cutin, suberine and wax biosynthesis	0.019411125	7	
sot00600	Sphingolipid metabolism	0.021856661	9	
sot00710	Carbon fixation in photosynthetic organisms	0.029264501	19	
sot04712	Circadian rhythm - plant	0.0300729	12	
sot00230	Purine metabolism	0.032032208	25

### Comprehensive analysis of differential expression of signal transduction pathway genes

The KEGG enrichment analysis of the DEGs shared by GJS-8 and XGH during tuberous root expansion showed that they were significantly enriched in many signal transduction pathways. Furthermore, these DEGs were annotated using NR, GO, and KEGG annotations, and a large number of DEGs were involved in signal transduction, cell wall, cell division, starch and sucrose metabolism pathways, indicating that signal transduction pathways played an important role in the process of sweet potato tuberous root expansion. Therefore, we analyzed the related genes of these pathways.

### Hormone signal

In this study, a total of 58 genes related to biosynthesis, metabolism and signal transduction of various hormones were identified (Table S4). The auxin signal transduction pathway was the most active, followed by ethylene signal transduction pathway. The genes related to hormone signal transduction in two varieties at the same developmental stage were further analyzed. In the auxin pathway, 3 AUX/IAA (Tai6.27980, Tai6.39648, and Tai6.22518) and 1 CH3(Tai6.36369) were significantly up-regulated in R1 phase; 1 AUX1(Tai6.1708), 1 SAUR (Tai6.14155), 1 AUX/IAA (Tai6.27980), and 2 ARF (Tai6.44587, Tai6.23113) were significantly up-regulated in R3 phase. In the ethylene pathway, 1 ERF (Tai6.17891) was significantly up-regulated in R1 phase, 1 ETR (Tai6.12247), 1 SIMKK (Tai6.10820) and 1 ERF (Tai6.10820) were significantly up-regulated in R2 phase, 5 ethylene-related genes (ETR: Tai6.12247, SIMKK: Tai6.10820, EIN2: Tai6.36354, EBF: Tai6.54900, and EIN3: Tai6.48960) were significantly up-regulated in R3 phase. In cytokinin signal transduction pathway, 1 AHP (Tai6.10485) was significantly enhanced during tuberous root development. In the abscisic acid pathway, 1 PYR/RYL (Tai6.18308) was significantly up-regulated in R1and R3 phase, 1 ABF (Tai6.48900) was significantly up-regulated in R2 phase. In the gibberellin pathway, 1 TF (Tai6.39357) was significantly up-regulated in R2, R2 and R3 phase. In the brassinolide pathway, 2 CYCD3(Tai6.43006, Tai6.37902) were significantly up-regulated in R2 phase. In the salicylic acid pathway, 2 NPR1(Tai6.32738, Tai6.52704) were significantly up-regulated in R1 phase,1 NPR1(Tai6.52704) was significantly up-regulated in R1 phase.

### MAPK, calcium and phospholipid signaling

Among the DEGs shared by XGH and GJS-8, 1 mitogen-activated protein kinases (MAPK) gene (Tai6.51134) was up-regulated in whole expansion stage, 1 MAPK (Tai6.44720) was up-regulated in R1 and R2 stages, 1 MAPK (Tai6.10820) was up-regulated in R2 and R3 stages, 4 MAPK (Tai6.53239, Tai6.7760, Tai6.9123, and Tai6.4327) were up-regulated in R3 stage, 10 MAPK genes were down-regulated during whole expansion stage, 10 MAPK genes were down-regulated in R2 and R3 stages (Table S5).

A total of 147 calcium signal related to genes, including 36 calcium-dependent protein kinases (CDPKs), 40 calcium-binding proteins (CBPs), 45 calmodulin/calmodulin-binding protein (CaM/CaM-binding), and 26 Calreticulin (CBL) were identified from the common DEGs of two varieties (Table S6). It is worth noting that most of genes were down-regulated in whole expansion stage.

A total of 22 phospholipid signal-related genes were identified from the common DEGs of two varieties (Table S7). Among them, 6 genes were significantly up-regulated in R1, R2 and R3 stages, 4 genes were significantly down-regulated in R1, R2 and R3 stages.

### Light signal

Sixty-five photoperiod related genes were identified as DEGs shared by XGH and GJS-8 during tuberous root development (Table S8). These genes included 20 CONSTANS-likes (COL), 5 phototropins, 14 GATA transcription factors (GATA), 12 LOB domain-containing proteins (LOB), 6 COP-interactive proteins genes (COP) and 8 phytochromes. In R1 stage, 15 genes were significantly up-regulated, including 3 phototropins, 4 COLs, 1 LOB, 1 COP and 6 phytochromes. In R2 stage, 21 genes were significantly up-regulated, including 7 COLs, 1 phototropin, 1 GATA, 3 LOBs, 3 COPs and 6 phytochrome genes. In R3 stage, 24 genes, including 3 phototropins, 8 COLs, 1 GATA, 4 LOBs, 4 COPs and 6 phytochromes, were significantly up-regulated.

### Cell wall and cell cycle

We identified 95 genes related to cell wall and cell cycle from the DEGs shared by GJS-8 and XGH (Table S9), including 29 xyloglucan endotransglucosylase/hydrolases (XTH), 22 expansins, 3 extensins, 8 cell division proteases (FtsZ), 6 cell division cycle 5-like proteins (CDC5), 9 cell division control proteins (CDC), 7 cyclin-dependent kinases (CDKs) and 11 cyclin-dependent kinase inhibitors (CDKIs). Among these genes, most of XTH and CDC genes were down-regulated, and most of the genes related to FtsZ, CDC5 and CDKIs were up-regulated.

### Starch and sucrose metabolism

Seventy genes related to starch and sucrose metabolism were identified from the DEGs shared by GJS-8 and XGH (Table S10), including 13 sucrose synthases (SuSy), 2 sucrose phosphate synthases (SPS), 10 starch synthases (SS), 5 invertase genes (INV), 10 granule-bound starch synthases (GBSS), 4 soluble starch synthases (SSS), 11 starch branching enzymes (SBE), 5 Beta-amylases, 5 alpha-amylases, and 5 isoamylases. Most of the genes were significantly up-regulated during the root expansion stage in sweet potato, and only a few genes were down-regulated.

### Transcription factor

**Fig. 4:**
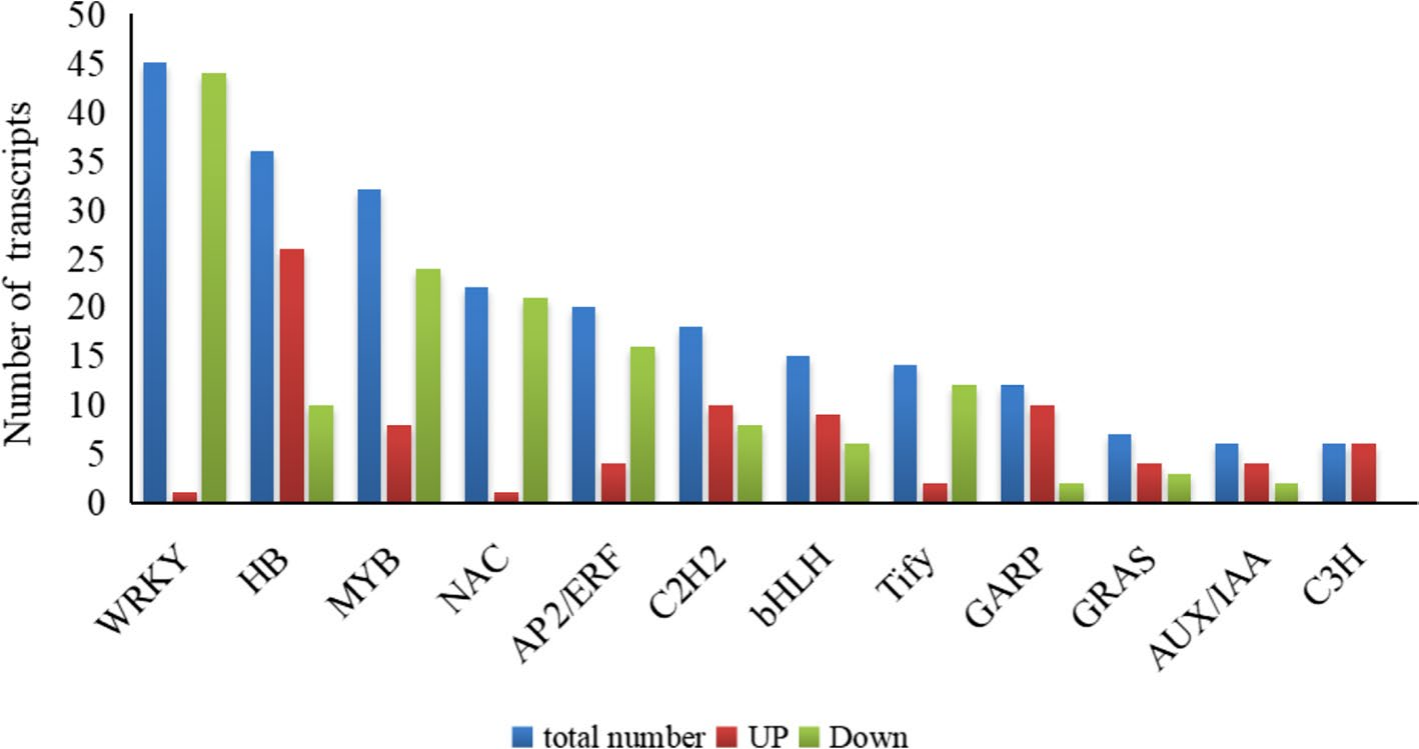
The number of transcription factors expressed significantly differentially in the R1, R2 and R3 stages of tuberous root expansion in GJS-8 and XGH

**Fig. 5:**
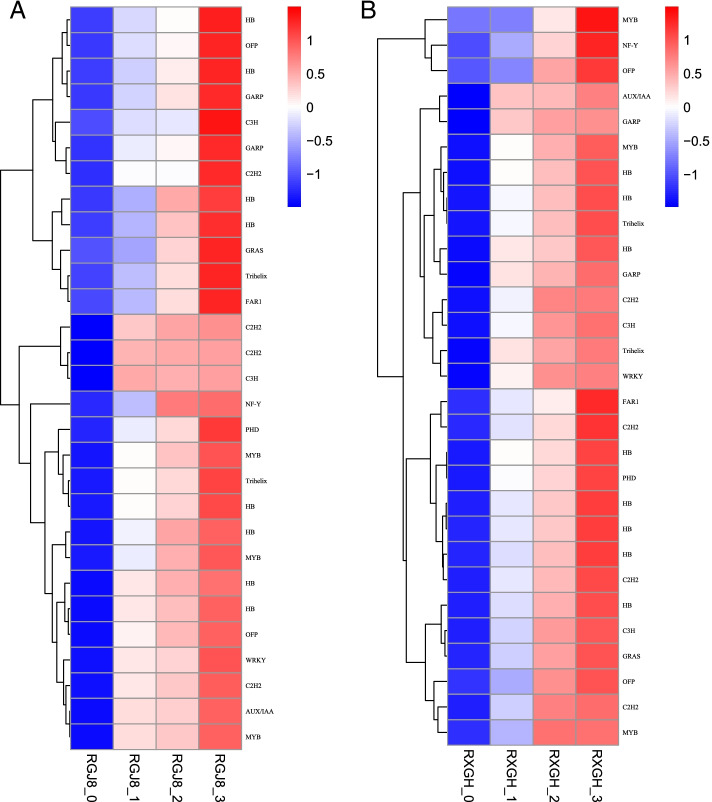
Heat map of highly expressed transcription factors. Every row shows a different TF gene. Red, white, and blue indicate slow, middle and high levels of mRNA expression, respectively. (**a**) Expression of transcription factors in GJS-8; (**b**) Expression of transcription factors in XGH

In this study, 296 TF genes were identified as DEGs shared by GJS-8 and XGH. Among them, 126 TFs were up-regulated, and 170 TFs were down-regulated during the tuberous root development (Table S11). WRKYs, HBs, MYBs were the major represented TF families (Fig. 4). Twenty-nine transcription factors in these families were significantly up-regulated, and their expression levels increased successively in the R1, R2 and R3 stages of tuberous root development in two cultivars, it mainly included the family of HB, C2H2, MYB transcription factors (Fig. 5).

### Weighted gene co-expression network analysis

**Fig. 6:**
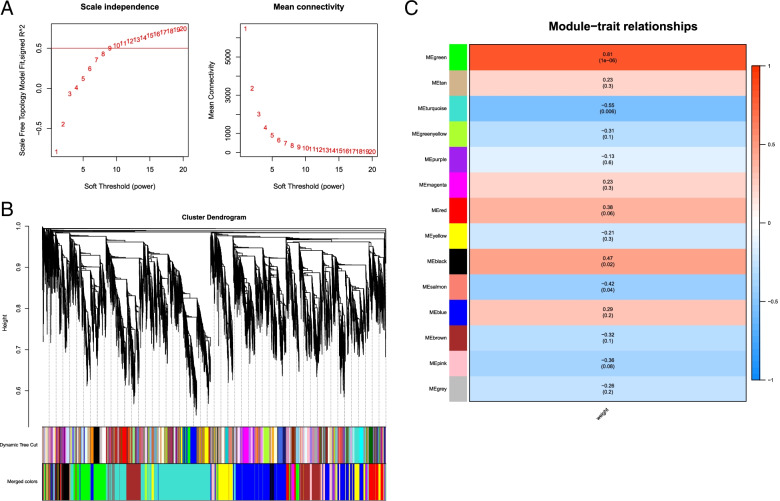
Soft-thresholding values estimation and module identification. **a** Scale independence and mean connectivity of various soft-thresholding values (β). **b** Dendrogram of all filtered genes enriched according to a dissimilarity measure (1-TOM) and the cluster module colors. **c** Heatmap of the correlation between the root tuber expansion traits and MEs of bladder cancer. The darker the module color, the more significant their relationship

To further understand the relationship between gene expression and tuberous root development, the weighted gene co-expression network analysis (WGCNA) was performed. In this study, β (soft-power threshold) = 9 was set to guarantee high scale independence and low mean connectivity (near 0) (Fig. 6A). The dissimilarity of the modules was set as 0.75, and a total of 14 modules were generated (Fig. 6B). The module trait relationship was shown in Fig. 6C. Green modules are highly related to tuberous root development (r > 0.80, *p* < 0.005). GO enrichment analysis was further carried out on the genes of green module (Table S12). The result showed that the biological processes were the most enriched in this module related to energy metabolism and transport. In addition, it was also significantly enriched in mRNA processing, hormone response, endogenous stimulus response and stress response. KEGG enrichment analysis showed that the green module was significantly enriched in transcription factors, plant circadian rhythm (sot04712), MAPK plant signal pathway (sot04016), and plant hormone signal transduction (sot04075) (Table S13).

**Fig. 7:**
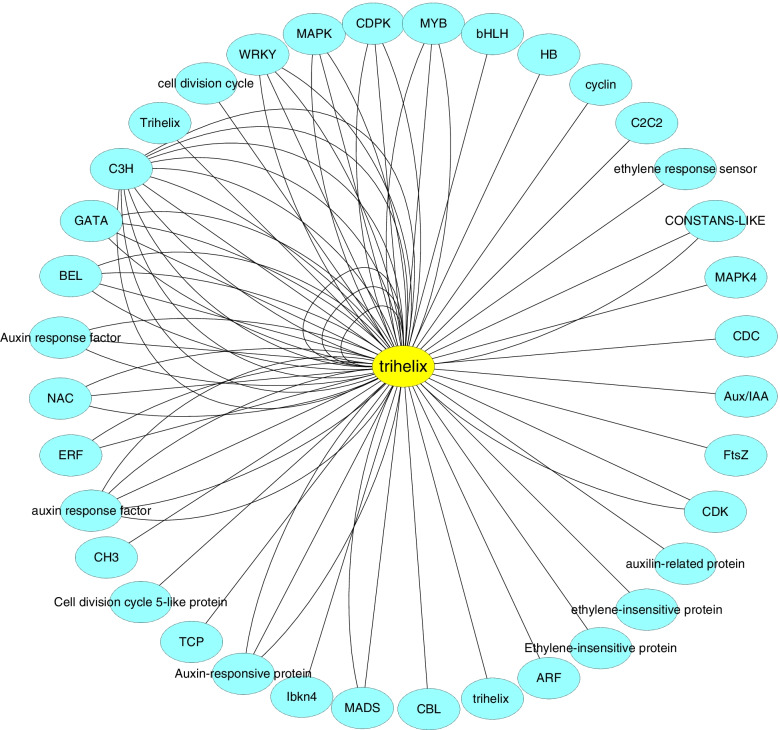
Trihelix transcription factor interactions based on co-expression pattern

The gene connectivity in the modules represents the regulatory relationship between the gene and other genes. The higher the connectivity, the greater the regulatory role of the gene in the modules, the more likely it was a hub gene. The gene with the highest connectivity in the green module was selected as the core gene of the module. This gene encoded a trihelix transcription factor (Tai6.25300). The homology of this gene in Arabidopsis is AT1G13450.1 (trihelix transcription factor: GT-1). A total of 1272 genes interacted with trihelix, including genes related to light signaling, calcium signaling, and plant hormone signaling, implying the processes the genes involved were potentially co-regulated. The interaction network of core genes was visualized by Cytoscape software. Because there were many genes interacting with hub genes, only partial genes were shown here (Fig. 7).

### Genes with significant differences in tuberous root development between two varieties

Taking | log2 (FoldChange) | > 1 and padj < 0.05 as the standard, we identified 18,028 differentially expressed genes (DEGs) for the GJS_8 vs. XGH (R1, R2 and R3), of which 12,792 were in R1 stage, 9979 in R2 stage and 8828 DEGs in R3. KEGG enrichment analysis showed that the up-regulated genes were significantly enriched to phenylpropanoid biosynthesis (sot00940), flavonoid biosynthesis (sot00941), starch and sucrose metabolism (sot00500) and pentose and glucuronate interconversions pathway (sot00040) in stage R1. In stage R2, the up-regulated genes were significantly enriched to the flavonoid biosynthesis pathway (sot00941). In R3 stage, the up-regulated genes were not significantly enriched to any pathway. In addition, 88 MYB, 86 bHLH, 3 WD40 transcription factors, and 30 anthocyanin biosynthesis related genes [6 trans-cinnamate 4-monooxygenases (C4H), 12 4-coumarate--CoA ligases (4CL), 8 chalcone synthases (CHS), 2 chalcone-flavanone isomerases (CHIL), 2 leucoanthocyanidin dioxygenases (LDOX/ANS)] were identified from these DEGs (Table S14). The difference of these anthocyanin related genes was the greatest in the R1 stage of the two varieties, and the difference was more than 10 times.

### Verification of gene expression patterns by qRT-PCR

**Fig. 8:**
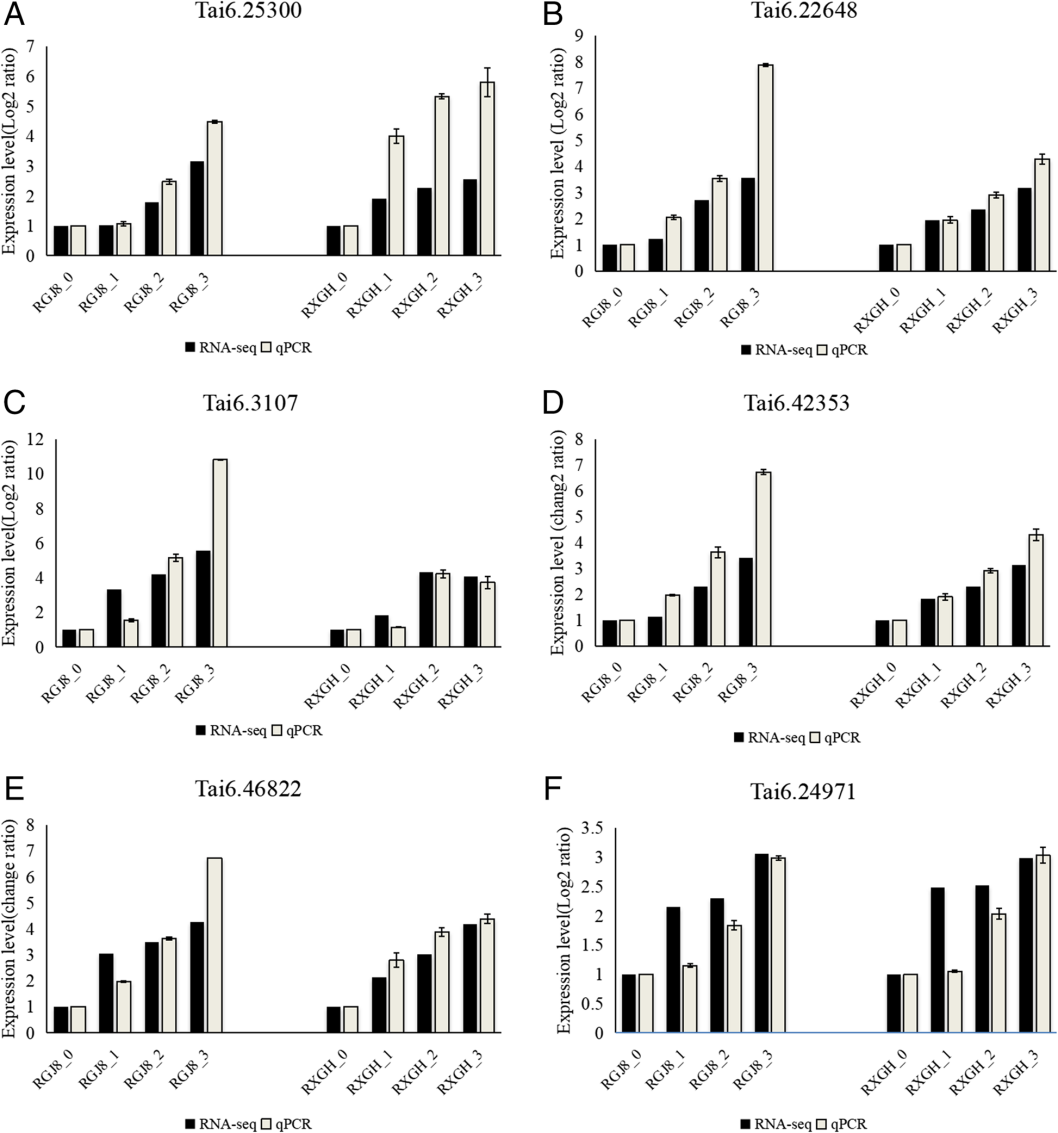
qRT-PCR validation profiles of six randomly selected genes. The data was normalized by using UBI as an internal reference. The expression level of fibrous root(R0) in each cultivar was used as reference state, which was set to 1, and fold change values were shown here. (**a**) Trihelix transcription factor (Tai6.25300); (**b**) BEL (Tai6.22648); (**c**) CONSTANS-like (Tai6.3107); (**d**) BEL (Tai6.42353); (**e**) BEL (Tai6.46822); (**f**) auxin-responsive protein (Tai6.24971)

In order to verify the accuracy of RNA-Seq results, we randomly selected 6 genes (Tai6.25300, Tai6.22648, Tai6.3107, Tai6.42353, Tai6.46822, and Tai6.24971) for qRT-PCR analysis. The results showed that the expression pattern of these 6 differential genes was similar to that of RNA-Seq (Fig. 8). The results indicated that the RNA-Seq was reliable.

## Discussion

The formation and development of the tuberous root of sweet potato is a complex process, which mainly involves the formation of vascular cambium and secondary cambium. After the formation of round vascular cambium, the tuberous root begins to thicken, then the cells continue to proliferate and expand to form a secondary cambium, which is accompanied by the continuous accumulation of starch and other substances, resulting in the continuous enlargement of the tuberous root.

Previous studies showed that the meristems are always active during tuberous root bulking, the transcriptome data obtained in this study reveal that the regulators of meristem development, such as LBD4 (LOB domain-containing protein 4, Tai6.18322, and Tai6.27010), WOX4 (WUSCHEL HOMEOBOX RELATED 14, Tai6.17770, and Tai6.44989) were significantly upregulated at tuberous root development, which is consistent with the results of previous studies [23]. Moreover, the genes are involved in cell division, including cell division protein FtsZ (FtsZ), cell division cycle 5 (CDC5), cell division control protein (CDC), and cyclin-dependent kinase (CDK), their expression levels were significantly enhanced in the tuberous root expansion stage (Table S9). The Genes involved in cell extension and expansion, including extension, XET, and expansin, also were significantly enhanced in the tuberous root expansion stage (Table S9). These results indicate that the formation and development of tuberous roots are inseparable from the active meristems and cell division.

A series of studies have shown that the initiation and induction of root/tuber is affected by the environment. For potatoes, photoperiod is essential for tuber formation [24]. Moreover, light is also important for the expansion of *Rehmannia glutinosa* tuberous root [25]. Photoperiod response protein, lateral organ boundaries protein (LOB), and GATA transcription factor are important members of photoperiod regulation. In this study, the expression of LOB (Tai6.27900) and GATA (Tai6.27468) were significantly enhanced during the tuberous root expansion stage. Furthermore, genes related to light signal transduction including phototropin, CONSTANS, and COP-interactive proteins were also significantly enhanced during the tuberous root expansion stage (Table S8). However, their peaks and expression patterns were obviously different, suggesting that light regulation is very critical to tuberous root formation and continuous development.

Moreover, genes detected in the roots may also be transcribed in the leaves and then transported to the root. For example, after being transcribed in leaves, potato stBEL5 mRNA was transported through the phloem to the stolon tip for translation into protein, thereby promoting the formation of storage organs [26]. In this study, 14 BELs genes were consistently up-regulated during the tuberous root expansion stage (Table S8), which suggest that these genes may be functionally similar to the stBEL5. Although the storage organs of potato and sweet potato are different, they may have similar regulatory systems. Therefore, they may be involved in light signal-regulated tuberous root development via similar mechanisms.

### The relationship between hormones and tuberous root swelling

Hormones are important signals in plant root development [27, 28]. In this study, the plant hormone signal transduction pathway was one of the most enriched KEGG pathways in tuberous root expansion stage. Auxin plays an important role in cambium cell proliferation and cell expansion [12], also maintains the meristem state of cambium cells and increase the number of xylem elements [29]. In the studies of radish, *Rehmannia glutinosa* and *Callerya speciosa*, the expressions of auxin-related genes were significantly up-regulated during tuberous root expansion stage [25, 30, 31]. In this study, 7 auxin-related genes (AUX / IAA, ARF, SAUR, and CH3) were up-regulated in tuberous root expansion stage, implying that they may relate to cell expansion in the secondary growth of cambium.

The results showed that cytokinin was involved in the proliferation and development of cambium cells, and the expression reached the highest level in the rapid growth stage of tuberous root, which was related to the development and formation of tuberous root / tuber [29, 32–34]. In this study, the expression of cytokinin related gene (Tai6.10485) was significantly up-regulated during tuberous root expansion, suggesting that cytokinin may promote root expansion by participating in the development of cambium.

Ethylene is a key regulator of rhizome induction and development [35], which promotes tuber formation by inhibiting GA biosynthesis [36]. Moreover, it has been shown that GA, auxin,and ethylene affect cell growth in the root by opposing the action of DELLA proteins. In this study, the expressions of ethylene-related genes were significantly up-regulated during tuberous root expansion (Table S4). Overall, these results suggest that these hormone signals related genes play vital roles during the tuberous root expansion stage.

### Multiple signal pathways are activated to regulate tuberous root development

Cellular processes involved in a series of signaling pathways are usually triggered by specific stimuli and hormones. Phospholipid signal plays an important role in root growth, cell division, and hormone regulation [37, 38]. It was reported that the expression levels of phospholipid signal-related genes/proteins were increased in the early stage of tuberous root expansion in *Rehmannia glutinosa*. In addition, the phospholipid-calcium signal system regulated potato tuber formation [25, 39]. In this study, 6 phospholipid signal-related genes were up-regulated in the stage of tuberous root expansion in GJS-8 and XGH, and the expression profiles in two varieties were quite similar, indicating that phospholipid signal was involved in the initiation and of tuberous root expansion.

Calcium is one of the main nutrients and is involved in almost the whole process of plant growth, including the controls of cell division, differentiation, and stress response as the second messenger [40, 41]. Studies revealed that CDPK played a role in the signal pathway of root initiation in potato and cassava, and exogenous calcium levels could affect the quantity and weight of potato tuber [42–44]. In addition, Ca^2+^ concentration and calcium signal- related genes (CBP, CBL, CaM, and CDPK) were significantly up-regulated during tuberous root formation in *Rehmannia Glutinosa *[25]. In this study, there was an increase in the stage of tuberous root expansion in the expression level of calcium signaling-related genes, including 9 CDPKs, 8 CBLs, and 1 CaM (Table S6), which suggests that calcium signal is involved in the formation and expansion of tuberous root in sweet potato. In addition, some genes related to the MAPK signaling pathway were up-regulated during tuberous root expansion development (Table S5), suggesting that the MAPK signal participats in the initiation and expansion of tuberous root formation. It has been shown that the MAPK signal plays an important role in cell cycle regulation, hormone, and stress response  [45].

### Transcription factor regulation and weighted gene co-expression network analysis

Transcription factors play an important role in the regulation of plant growth and development and secondary metabolism. Many transcription factors have been identified to play key roles in organ development, including MADS, bHLH, MYB, NAC, GRAS et al. In this study, we identified 29 transcription factors that were significantly up-regulated during the tuberous root expansion stage in two varieties. Their expression levels increased successively (Fig. 5). Among these TFs, MYBs and HBs were the main transcription factors with large up-regulation multiples. One trihelix transcription factor gene (Tai6.25300) was identified as a tuberous root expansion-related gene through WGCNA analysis, its homologous gene in Arabidopsis was AT1G13450.1(Trihelix, GT-1), which was considered to be a molecular switch responded to light signals through Ca^2+^-dependent phosphorylation/dephosphorylation [46]. The Trihelix factor is a plant-specific triple helix DNA binding transcription factor. Many studies have proved that the trihelix transcription factor was involved in plant light response [47, 48]. In this study, the expression of light signal related-genes was coordinated with Tai6.25300, and significantly up-regulated during tuberous root development. Moreover, qRT-PCR confirmed that the expression of Tai6.25300 was up-regulated and increased successively during tuberous root development in two varieties, suggesting that Tai6.23500 was closely related to tuberous root development. We infer that Tai6.25300 participates in tuberous root expansion by positively regulating light signal related genes.

MYBs were involved in cell cycle regulation, plant morphogenesis, cell wall synthesis, secondary metabolism, xylem/phloem differentiation, root radial pattern formation, and so on [49, 50]. Furthermore, previous studies have found that the transcriptional level of MYB was significantly up-regulated during rhizome development [30, 51], and MYBs were highly expressed at the rapid thickening stages of *Callerya speciosa *[36]. In this study, 32 MYB transcription factors were significantly differentially expressed as tuberous root development, of which 8 were significantly up-regulated. Homeodomain (Homebox, HB) transcription factors are very important regulatory proteins in plants, which are mainly divided into 14 categories, including KNOX, BEL, and HD-ZIP, etc. Arabidopsis HB transcription factors were involved in cell division, differentiation, replication, growth, and regulation of the early development of vascular tissue [52, 53], In addition, the members of HB family were also involved in the regulation of cambium cell differentiation to phloem and lignin biosynthesis [54, 55]. RNA-Seq data revealed that 3 homeobox genes were notably upregulated during the formation and thickening of storage roots [22]. In this study, 36 HB transcription factors were significantly differentially expressed in tuberous root development, of which 26 were significantly up-regulated.

To sum up, these results suggest that transcription factors may drive root/stem growth through cell cycle regulation, cell division, and secondary wall strength. The TFs revealed in this study may be the important candidate genes for breeding sweet potato with high production in the future.

### Starch and sucrose metabolism regulation

Sucrose and starch accumulation occurs during the bulking of storage roots, they are considered to be one of the most important carbohydrates, and play an important role in the formation of storage organs. Sucrose invertase and sucrose synthase were involved in the introduction and accumulation of sucrose in storage roots [56]. In addition, sucrose synthase was related to the tuber /tuberous root growth of potato and radish and was a key enzyme in the early development of radish storage root [57–60]. In this study, 5 SuSy genes were significantly up-regulated during tuberous root development in GJS-8 and XGH, while 2 INV genes were significantly down-regulated (Table S10), Invertase was active in fibrous roots of sweet potato but rapidly decreased to an undetectable level during storage root development [61]. Furthermore, Jackson showed that high content of sucrose was required as a necessary condition during the formation of storage organs [62]. In the present study, SPS (Tai6.24187), the major source of sucrose synthesis activity [63], was up-regulated during tuberous roots expansion. This result was consistent with previous studies in radish that found up-regulation of SPS playing a major role in the thickening stage of radish taproot [64].

The accumulation of starch occurs at the same time as the expansion of storage organs. It has shown that the expansions of potato and lotus root tubers were highly coordinated with the accumulation of starch [65, 66]. The expansion of cassava root was synchronized with the accumulation of starch [67], and granule-bound starch synthase (GBSS) has been shown to affect starch synthesis in storage organs [68]. In this study, 22 starch-related genes (6 GBSSs, 4 SSSs, 8 SBEs, and 4 isoamylases) were significantly up-regulated during root tuber expansion (Table S10), which was similar to previous studies. SBE, GBSS, and SS-related genes were significantly up-regulated during root expansion of *Panax notoginseng *[69]. These starch and sucrose metabolism genes play important roles in tuberous root expansion.

### Genes with significant differences in tuberous root development between two varieties

GJS_8 and XGH are two varieties with different anthocyanin content. GJS_8 has higher anthocyanin content than XGH. Anthocyanins are water-soluble pigments and an important class of flavonoids. We found that there was a large number of genes with significant differences in tuberous root development between two varieties. KEGG enrichment analysis showed that the DEGs were significantly enriched to phenylpropanoid biosynthesis (sot00940), flavonoid biosynthesis (sot00941), and starch and sucrose metabolism pathway (sot00500). It was also found that phenylpropanoid biosynthesis and flavonoid biosynthesis was significantly enriched in the process of anthocyanin biosynthesis [70]. In addition, we identified a large number of MYB, bHLH, WD40 transcription factors, and anthocyanin biosynthesis genes from these differential genes, including 6 MYBs,17 bHLHs, 3 C4Hs, 5 4CLs,6 CHSs, 2CHILs, and 2 LDOX/ANSs, which were significantly differentially expressed between GJS_8 and XGH and also significant differentially expressed between tuberous root and fiber root, especially in GJS_8 tuberous root. A large number of studies have shown that MYB, bHLH, and WD40 transcription factors were the regulators of flavonoid biosynthesis, and the results also showed that *IbMYB1* controls the biosynthesis of anthocyanins in sweet potato [71]. It was found that 10 anthocyanin biosynthesis genes were significantly up-regulated during *Aronia melanocarpa* fruit development [72]. Hence, it shows that anthocyanin biosynthesis related-genes may be involved in the tuberous root development in sweet potato, and their regulatory mechanism should be studied in the next step.

### Regulatory networks associated with tuberous root development

**Fig. 9:**
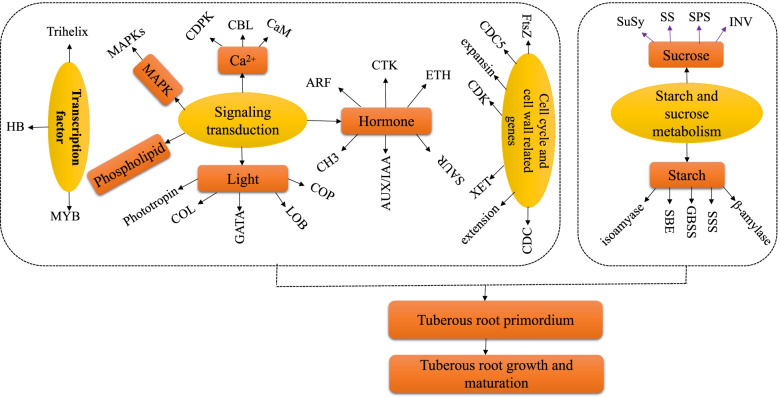
A hypothetical model of regulatory network related to tuberous root expansion in sweet potato

Tuberous root development is a complex regulatory process, which is affected by many factors. In this study, through transcriptome analysis, combined with previous research results, a hypothetical model of sweet potato tuberous root development regulatory network is proposed (Fig. 9). The cells in the vascular cambium divide and expand continuously to produce secondary xylem and secondary phloem, resulting in the expansion of tuberous root. Cell proliferation is regulated through several signal transduction pathways (light, Phospholipid, calcium, MAPK, hormone, and transcription signaling) and metabolism possesses (cell wall, sucrose, and starch metabolism). Several genes including photoperiod (LOB, GATA, Phototropin, COL, and COP), calcium signal (CDPK, CBL, and CaM), MAPK signal, auxin-related genes (Aux/IAA, CH3, ARF, and SAUR), HB transcription factors (BELL, KNOX, and HD-ZIP), are highly expressed to promote cell differentiation, division, expansion and sucrose and starch accumulation at the secondary structure. In addition, FtsZ, CDC, CDK, XTH, expansin, and extension, are involved in cell division extension and expansion. Finally, SuSy, SPS, SSS, GBSS, and SBE are involved in the hydrolysis of sucrose and the synthesis of starch. Further functional identification studies were needed to confirm the functions of these potential genes.

## Conclusion

Integrated transcriptomic and WGCNA analyses were performed in the study, there were 15,920 differential genes shared by XGH and GJS-8. GO and KEGG pathway enrichment analysis revealed that these DEGs were mainly involved in plant hormone signal transduction, starch and sucrose metabolism, MAPK signal transduction, light signal, phospholipid signal, calcium signal, transcription factor, cell wall, and cell cycle. Furthermore, WGCNA and qRT-PCR analysis suggested that Tai6.25300 played an important role in tuberous root development in sweet potato. A hypothetical model of a genetic regulatory network associated with tuberous roots in sweet potato is put forward. The tuberous root development of sweet potato is mainly attributed to cell differentiation, division, and expansion, which are regulated and promoted by certain specific signal transduction pathways and metabolism processes. These findings can not only provide novel insights into the molecular regulation mechanism of tuberous root expansion, but also support theoretical basis for genetic improvement of sweet potato.

## Materials and methods

### Materials

**Fig. 10:**
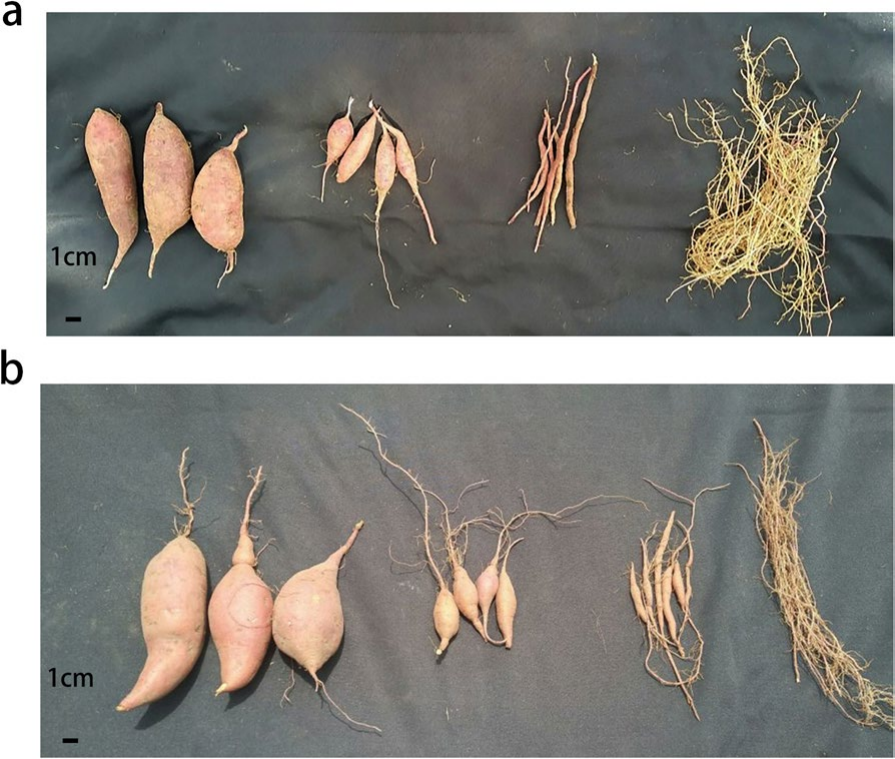
Anatomical diagram with sampling diagram. **a** GJS_8; **b** XGH

Two sweet potato varieties, GJS-8 and XGH were used in this study. They were planted in the experimental farm of Hepu Institute of Agricultural Science in Beihai, Guangxi. At 90 days after planting, Sample collection refers to Ku et al’s method [14], Fibrous roots (R0:RGJ8_0, RXGH_0; 1 mm diameter) and developing tuberous roots [(R1:RGJ8_1, RXGH_1; 1 cm diameter, less than 2 g), (R2:RGJ8_2, RXGH_2; 3 cm diameter, 5-10 g), (R3:RGJ8_3, RXGH_3; 5 cm diameter, approx 50 g)] were collected,respectively (Fig. 10). Three plants were selected randomly from every repetition each time. At least five roots were mixed as a biological biological repetition. For the big tuberous root samples, five fresh tuberous roots from a repetition were washed with distilled water, cut down into slices, and mixed as a biological repetition. Three biological replicates were performed. The samples were stored at − 80 °C for extracting total RNA.

### RNA extraction, cDNA library construction, and RNA-Seq

A conventional trizol method was used to extract RNA from the samples. The concentration and purity of total RNA were determined by a NanoPhotometer® spectrophotometer (IMPLEN, CA, USA). RNA integrity was assessed using the RNA Nano 6000 Assay Kit of the Bioanalyzer 2100 system (AgilentTechnologies, CA, USA). Sequencing libraries were generated using NEBNext®UltraTM RNA Library Prep Kit for Illumina® (NEB, USA).

### RNA sequencing and data analysis

3 μg total RNA from each sample was used as the input material, fragmentation was carried out using divalent cations under elevated temperature in NEBNext First Strand Synthesis Reaction Buffer (5X). First strand cDNA was synthesized using random hexamer primer and M-MuLV Reverse Transcriptase (RNase H-). Second strand cDNA synthesis was subsequently performed using DNA Polymerase I and RNase H. Remaining overhangs were converted into blunt ends via exonuclease/polymerase activities. After adenylation of 3′ ends of DNA fragments, NEBNext Adaptor with hairpin loop structure were ligated to prepare for hybridization. In order to select cDNA fragments of preferentially 250 ~ 300 bp in length, the library fragments were purified with AMPure XP system (Beckman Coulter, Beverly, USA). Then 3 μl USER Enzyme (NEB, USA) was used with size-selected, adaptor-ligated cDNA at 37 °C for 15 min followed by 5 min at 95 °C before PCR. Then PCR was performed with Phusion High -Fidelity DNA polymerase, Universal PCR primers and Index (X) Primer. At last, PCR products were purified (AMPure XP system) and library quality was assessed on the Agilent Bioanalyzer 2100 system. Clean reads were obtained by removing reads containing an adapter, reads containing ploy-N and low-quality reads from the raw data. The clean reads were then aligned with the sweet potato genome (http://public-genomes-ngs.molgen.mpg.de/cgi-bin/hgGateway?hgsid=9052&clade=plant&org=Ipomoea+batatas&db=ipoBat4 ) [23]. Feature Counts v1.5.0-p3 was used to count the read numbers mapped to each gene, and the FPKM of each gene was then calculated based on the length of the gene and the read count mapped to the gene [23]. Genes with an adjusted *P*-value < 0.05 and | log2 (FoldChange) | > 1 obtained by DESeq2 were considered DEGs.

### Functional annotation

Gene Ontology (GO) enrichment analysis of the DEGs was implemented using the cluster Profiler R package, and the gene length bias was corrected during this process [73]. KOBAS software was used to test the statistical enrichment of the DEGs in Kyoto Encyclopedia of Genes and Genomes (KEGG) pathways [74]. To obtain more information about the DEGs, the DEGs were annotated using seven databases: NR (NCBI nonredundant pro-tein), NT (NCBI Nucleotide Sequences), Gene Ontology (GO), KO (KO, KEGG Orthology), KOG (Eukaryotic Or Thologous Groups), Pfam (Protein Family Database) and Swiss-Prot (a manually annotated and reviewed protein sequence database). All the DEGs were subjected to hierarchical clustering analysis using the average linkage method [75].

### Weighted gene co-expression network analysis

The DEGs detected with DESeq2 were combined and the TPM values for the 24 samples were determined. Each TPM value was increased by 0.01 and further transformed by a log10 calculation. The converted data were analyzed with the R package WGCNA (version 1.66), with a power value of 9 [76, 77].

### Validation of the DEGs data using qRT-PCR

Total RNAs were extracted from the tuberous samples (fibrous root, tuberous roots less than 2 g, tuberous roots 5-10 g, tuberous roots greater than 50 g) with Trizol® Reagent (Magen, China). and then reverse transcribed into cDNA with HiScript III SuperMix for qPCR(+gDNA wiper) (Vazyme, China). qRT-PCR was carried out using SYBR Premix Ex TaqII Kit (TaKaRa, Dalian, China) on a Bio-Rad iQ5 Real-time PCR System (Bio-Rad Laboratories, CA, USA), Ten μl reaction solution contained 5 μl SYBR Green I Master, 1 μl specific Primer, 1 μl cDNA samples, 3 μl RNase-Free H2O. One-third dilution of the cDNA sample was used, and the reaction conditions were: 30s at 95 °C followed by 40 cycles of 30s at 95 °C, and 30s at 60 °C. Each sample had three biological replicates with three technical replicates for each biological replicate. The relative expression level was calculated by the equation ratio 2^-ΔΔCt^. The primers of selected genes were designed using primer 5 software (Table S15), and UBI gene was used as the internal control.

## Supplementary Information

**Additional file 1.**

## Data Availability

The materials of this study were provided by the College of Agriculture at Hepu Institute of Agricultural Science. Correspondence and requests for materials should be addressed to Longfei He (lfhe@gxu.edu.cn). The raw sequencing data have submitted to the NCBI SRA database (PRJNA678375).

## Abbreviations

CTK: Cytokinin
ABA: Abscisic acid
IAA: Auxin
GA: Gibberellin
MAPK: Mitogen-activated protein kinases
CDPK: Calcium-dependent protein kinases
CBP: Calcium-binding proteins
CaM/CaM-Binding: Calmodulin/calmodulin-binding protein
CBL: Calreticulin
COL: CONSTANS-like
XTH: Xyloglucan endotransglucosylase/hydrolases
FtsZ: Cell division proteases
CDC5: Cell division cycle 5-like proteins
CDC: Cell division control proteins
CDK: Cyclin-dependent kinasess
CDKI: Cyclin-dependent kinase inhibitors
SuSy: Sucrose synthases
SPS: Sucrose phosphate synthases
INV: Invertase genes
GBSS: Granule- bound starch synthases
SSS: Soluble starch synthases
SBE: Starch branching enzymes
WGCNA: Co-expression network analysis
