# Supplementary material for: Transcriptomic analysis of tuberous root in two sweet potato varieties reveals the important genes and regulatory pathways in tuberous root development
Source: BMC Genomics. 2022 Jun 27;23:473. doi: 10.1186/s12864-022-08670-x (PMC9235109; doi:10.1186/s12864-022-08670-x)
Supplement: Supplementary file 1 — Additional file 1. [file 12864_2022_8670_MOESM1_ESM.docx]

**Supplementary data**

**Table S1** In R1 stage, GJS-8 and XGH shared all DEGs GO enrichment significant pathway.

| Category | ID | Term | pvalue | Gene Number |
| --- | --- | --- | --- | --- |
| BP | GO:0009605 | response to external stimulus | 1.33683E-07 | 12 |
| BP | GO:0043207 | response to external biotic stimulus | 7.06955E-07 | 9 |
| BP | GO:0051707 | response to other organism | 7.06955E-07 | 9 |
| BP | GO:0098542 | defense response to other organism | 7.06955E-07 | 9 |
| BP | GO:0005984 | disaccharide metabolic process | 5.39652E-06 | 19 |
| BP | GO:0009311 | oligosaccharide metabolic process | 7.79391E-06 | 19 |
| BP | GO:0048583 | regulation of response to stimulus | 3.82139E-05 | 9 |
| BP | GO:0005992 | trehalose biosynthetic process | 6.29525E-05 | 12 |
| BP | GO:0046351 | disaccharide biosynthetic process | 9.92563E-05 | 12 |
| BP | GO:0009312 | oligosaccharide biosynthetic process | 0.000152171 | 12 |
| BP | GO:0051704 | multi-organism process | 0.000283484 | 17 |
| BP | GO:0005991 | trehalose metabolic process | 0.000332198 | 12 |
| BP | GO:0006721 | terpenoid metabolic process | 0.000937925 | 8 |
| BP | GO:0016114 | terpenoid biosynthetic process | 0.000937925 | 8 |
| BP | GO:0009607 | response to biotic stimulus | 0.000959984 | 14 |
| BP | GO:0046434 | organophosphate catabolic process | 0.00330176 | 7 |
| BP | GO:0044262 | cellular carbohydrate metabolic process | 0.004000535 | 42 |
| BP | GO:0005985 | sucrose metabolic process | 0.004956445 | 7 |
| BP | GO:0006066 | alcohol metabolic process | 0.004956445 | 7 |
| BP | GO:0019751 | polyol metabolic process | 0.004956445 | 7 |
| BP | GO:0009628 | response to abiotic stimulus | 0.007362117 | 6 |
| BP | GO:0006869 | lipid transport | 0.018081837 | 7 |
| BP | GO:0034637 | cellular carbohydrate biosynthetic process | 0.021808286 | 25 |
| BP | GO:1901615 | organic hydroxy compound metabolic process | 0.023460499 | 7 |
| BP | GO:0006979 | response to oxidative stress | 0.025981555 | 22 |
| BP | GO:0072330 | monocarboxylic acid biosynthetic process | 0.027122004 | 11 |
| BP | GO:0010876 | lipid localization | 0.029845362 | 7 |
| BP | GO:1901292 | nucleoside phosphate catabolic process | 0.032994072 | 5 |
| BP | GO:0006720 | isoprenoid metabolic process | 0.033766252 | 10 |
| BP | GO:0008299 | isoprenoid biosynthetic process | 0.033766252 | 10 |
| BP | GO:0006952 | defense response | 0.039557293 | 15 |
| BP | GO:0044723 | single-organism carbohydrate metabolic process | 0.045122985 | 47 |
| BP | GO:0071554 | cell wall organization or biogenesis | 0.047299209 | 13 |
| CC | GO:0005618 | cell wall | 0.029326157 | 16 |
| CC | GO:0030312 | external encapsulating structure | 0.029326157 | 16 |
| CC | GO:0045263 | proton-transporting ATP synthase complex, coupling factor F(o) | 0.03043615 | 4 |
| MF | GO:0043565 | sequence-specific DNA binding | 1.64757E-08 | 62 |
| MF | GO:0001071 | nucleic acid binding transcription factor activity | 6.63113E-07 | 101 |
| MF | GO:0005509 | calcium ion binding | 5.69988E-06 | 53 |
| MF | GO:0008661 | 1-deoxy-D-xylulose-5-phosphate synthase activity | 0.000426212 | 7 |
| MF | GO:0016744 | transferase activity, transferring aldehyde or ketonic groups | 0.000426212 | 7 |
| MF | GO:0016758 | transferase activity, transferring hexosyl groups | 0.000608095 | 82 |
| MF | GO:0004650 | polygalacturonase activity | 0.001080764 | 10 |
| MF | GO:0016157 | sucrose synthase activity | 0.002014716 | 7 |
| MF | GO:0016684 | oxidoreductase activity, acting on peroxide as acceptor | 0.002880778 | 27 |
| MF | GO:0008047 | enzyme activator activity | 0.00354194 | 10 |
| MF | GO:0050660 | flavin adenine dinucleotide binding | 0.005243868 | 23 |
| MF | GO:0048037 | cofactor binding | 0.006855284 | 78 |
| MF | GO:0004420 | hydroxymethylglutaryl-CoA reductase (NADPH) activity | 0.00719232 | 6 |
| MF | GO:0004620 | phospholipase activity | 0.00719232 | 6 |
| MF | GO:0016298 | lipase activity | 0.00719232 | 6 |
| MF | GO:0016835 | carbon-oxygen lyase activity | 0.007531424 | 22 |
| MF | GO:0016209 | antioxidant activity | 0.007783865 | 27 |
| MF | GO:0004601 | peroxidase activity | 0.008124503 | 25 |
| MF | GO:0016709 | oxidoreductase activity, acting on paired donors, with incorporation or reduction of molecular oxygen, NAD(P)H as one donor, and incorporation of one atom of oxygen | 0.01160741 | 5 |
| MF | GO:0004497 | monooxygenase activity | 0.011611141 | 7 |
| MF | GO:0005507 | copper ion binding | 0.015570664 | 15 |
| MF | GO:0010333 | terpene synthase activity | 0.016331919 | 12 |
| MF | GO:0004089 | carbonate dehydratase activity | 0.016935773 | 5 |
| MF | GO:0050662 | coenzyme binding | 0.019032448 | 49 |
| MF | GO:0004674 | protein serine/threonine kinase activity | 0.023408098 | 8 |
| MF | GO:0016836 | hydro-lyase activity | 0.02785153 | 10 |
| MF | GO:0060589 | nucleoside-triphosphatase regulator activity | 0.02785153 | 10 |
| MF | GO:0005096 | GTPase activator activity | 0.030655291 | 7 |
| MF | GO:0030170 | pyridoxal phosphate binding | 0.031126213 | 20 |
| MF | GO:0070279 | vitamin B6 binding | 0.031126213 | 20 |
| MF | GO:0008194 | UDP-glycosyltransferase activity | 0.034313172 | 30 |
| MF | GO:0016638 | oxidoreductase activity, acting on the CH-NH2 group of donors | 0.037531556 | 7 |
| MF | GO:0004553 | hydrolase activity, hydrolyzing O-glycosyl compounds | 0.038412237 | 62 |
| MF | GO:0016798 | hydrolase activity, acting on glycosyl bonds | 0.039610976 | 64 |
| MF | GO:0000287 | magnesium ion binding | 0.042470323 | 21 |
| MF | GO:0019842 | vitamin binding | 0.043965497 | 22 |

**Table S2** In R2 stage, GJS-8 and XGH shared all DEGs GO enrichment significant pathway.

| Category | ID | Term | pvalue | Gene Number |
| --- | --- | --- | --- | --- |
| BP | GO:0005984 | disaccharide metabolic process | 1.58568E-06 | 21 |
| BP | GO:0009311 | oligosaccharide metabolic process | 2.41392E-06 | 21 |
| BP | GO:0005992 | trehalose biosynthetic process | 5.9519E-06 | 14 |
| BP | GO:0051704 | multi-organism process | 7.77783E-06 | 21 |
| BP | GO:0046351 | disaccharide biosynthetic process | 1.06187E-05 | 14 |
| BP | GO:0009312 | oligosaccharide biosynthetic process | 1.82455E-05 | 14 |
| BP | GO:0072350 | tricarboxylic acid metabolic process | 2.32894E-05 | 10 |
| BP | GO:0005991 | trehalose metabolic process | 4.88383E-05 | 14 |
| BP | GO:0006022 | aminoglycan metabolic process | 9.6192E-05 | 9 |
| BP | GO:0006026 | aminoglycan catabolic process | 9.6192E-05 | 9 |
| BP | GO:0006030 | chitin metabolic process | 9.6192E-05 | 9 |
| BP | GO:0006032 | chitin catabolic process | 9.6192E-05 | 9 |
| BP | GO:0006040 | amino sugar metabolic process | 9.6192E-05 | 9 |
| BP | GO:0016998 | cell wall macromolecule catabolic process | 9.6192E-05 | 9 |
| BP | GO:0044036 | cell wall macromolecule metabolic process | 9.6192E-05 | 9 |
| BP | GO:0046348 | amino sugar catabolic process | 9.6192E-05 | 9 |
| BP | GO:1901071 | glucosamine-containing compound metabolic process | 9.6192E-05 | 9 |
| BP | GO:1901072 | glucosamine-containing compound catabolic process | 9.6192E-05 | 9 |
| BP | GO:0044262 | cellular carbohydrate metabolic process | 0.000103681 | 52 |
| BP | GO:0008037 | cell recognition | 0.000518802 | 15 |
| BP | GO:0009856 | pollination | 0.000518802 | 15 |
| BP | GO:0009875 | pollen-pistil interaction | 0.000518802 | 15 |
| BP | GO:0044702 | single organism reproductive process | 0.000518802 | 15 |
| BP | GO:0044706 | multi-multicellular organism process | 0.000518802 | 15 |
| BP | GO:0048544 | recognition of pollen | 0.000518802 | 15 |
| BP | GO:0048583 | regulation of response to stimulus | 0.000708849 | 8 |
| BP | GO:0000003 | reproduction | 0.000940932 | 15 |
| BP | GO:0022414 | reproductive process | 0.000940932 | 15 |
| BP | GO:0044712 | single-organism catabolic process | 0.001095692 | 33 |
| BP | GO:1901136 | carbohydrate derivative catabolic process | 0.001185212 | 10 |
| BP | GO:0006720 | isoprenoid metabolic process | 0.001304183 | 14 |
| BP | GO:0008299 | isoprenoid biosynthetic process | 0.001304183 | 14 |
| BP | GO:0006090 | pyruvate metabolic process | 0.001334672 | 23 |
| BP | GO:0044723 | single-organism carbohydrate metabolic process | 0.001431407 | 60 |
| BP | GO:0006099 | tricarboxylic acid cycle | 0.001449401 | 7 |
| BP | GO:0006101 | citrate metabolic process | 0.001449401 | 7 |
| BP | GO:0009060 | aerobic respiration | 0.001449401 | 7 |
| BP | GO:0015977 | carbon fixation | 0.001449401 | 7 |
| BP | GO:1901565 | organonitrogen compound catabolic process | 0.001622043 | 15 |
| BP | GO:0071554 | cell wall organization or biogenesis | 0.001973248 | 18 |
| BP | GO:0006721 | terpenoid metabolic process | 0.002009152 | 8 |
| BP | GO:0016114 | terpenoid biosynthetic process | 0.002009152 | 8 |
| BP | GO:0044724 | single-organism carbohydrate catabolic process | 0.002293361 | 23 |
| BP | GO:0009628 | response to abiotic stimulus | 0.002516704 | 7 |
| BP | GO:0016051 | carbohydrate biosynthetic process | 0.002517259 | 33 |
| BP | GO:0043207 | response to external biotic stimulus | 0.002986353 | 6 |
| BP | GO:0051707 | response to other organism | 0.002986353 | 6 |
| BP | GO:0098542 | defense response to other organism | 0.002986353 | 6 |
| BP | GO:0009141 | nucleoside triphosphate metabolic process | 0.004251759 | 32 |
| BP | GO:0016052 | carbohydrate catabolic process | 0.004512739 | 27 |
| BP | GO:0015980 | energy derivation by oxidation of organic compounds | 0.006331427 | 7 |
| BP | GO:0045333 | cellular respiration | 0.006331427 | 7 |
| BP | GO:0034637 | cellular carbohydrate biosynthetic process | 0.008307562 | 29 |
| BP | GO:0032787 | monocarboxylic acid metabolic process | 0.00896073 | 34 |
| BP | GO:0006096 | glycolytic process | 0.009229242 | 20 |
| BP | GO:0006757 | ATP generation from ADP | 0.009229242 | 20 |
| BP | GO:0009135 | purine nucleoside diphosphate metabolic process | 0.009229242 | 20 |
| BP | GO:0009179 | purine ribonucleoside diphosphate metabolic process | 0.009229242 | 20 |
| BP | GO:0009185 | ribonucleoside diphosphate metabolic process | 0.009229242 | 20 |
| BP | GO:0046031 | ADP metabolic process | 0.009229242 | 20 |
| BP | GO:0005985 | sucrose metabolic process | 0.009357019 | 7 |
| BP | GO:0006066 | alcohol metabolic process | 0.009357019 | 7 |
| BP | GO:0009605 | response to external stimulus | 0.009357019 | 7 |
| BP | GO:0019751 | polyol metabolic process | 0.009357019 | 7 |
| BP | GO:0006091 | generation of precursor metabolites and energy | 0.009813676 | 27 |
| BP | GO:0006165 | nucleoside diphosphate phosphorylation | 0.010715893 | 20 |
| BP | GO:0009132 | nucleoside diphosphate metabolic process | 0.010715893 | 20 |
| BP | GO:0046939 | nucleotide phosphorylation | 0.010715893 | 20 |
| BP | GO:0006950 | response to stress | 0.010816597 | 65 |
| BP | GO:0009144 | purine nucleoside triphosphate metabolic process | 0.012085935 | 28 |
| BP | GO:0009199 | ribonucleoside triphosphate metabolic process | 0.012085935 | 28 |
| BP | GO:0009205 | purine ribonucleoside triphosphate metabolic process | 0.012085935 | 28 |
| BP | GO:0006979 | response to oxidative stress | 0.014751477 | 25 |
| BP | GO:0046034 | ATP metabolic process | 0.017392149 | 27 |
| BP | GO:0010035 | response to inorganic substance | 0.01834732 | 7 |
| BP | GO:0030001 | metal ion transport | 0.024199702 | 37 |
| BP | GO:0006487 | protein N-linked glycosylation | 0.024573432 | 7 |
| BP | GO:0001101 | response to acid chemical | 0.026081143 | 6 |
| BP | GO:1901700 | response to oxygen-containing compound | 0.026081143 | 6 |
| BP | GO:0006814 | sodium ion transport | 0.026192042 | 5 |
| BP | GO:0016567 | protein ubiquitination | 0.03023778 | 14 |
| BP | GO:0032446 | protein modification by small protein conjugation | 0.03023778 | 14 |
| BP | GO:0009073 | aromatic amino acid family biosynthetic process | 0.036121451 | 8 |
| BP | GO:0008272 | sulfate transport | 0.03867614 | 9 |
| BP | GO:0072348 | sulfur compound transport | 0.03867614 | 9 |
| BP | GO:1901615 | organic hydroxy compound metabolic process | 0.041035414 | 7 |
| BP | GO:0009123 | nucleoside monophosphate metabolic process | 0.045785588 | 27 |
| BP | GO:0009126 | purine nucleoside monophosphate metabolic process | 0.045785588 | 27 |
| BP | GO:0009161 | ribonucleoside monophosphate metabolic process | 0.045785588 | 27 |
| BP | GO:0009167 | purine ribonucleoside monophosphate metabolic process | 0.045785588 | 27 |
| BP | GO:0042221 | response to chemical | 0.046752148 | 30 |
| CC | GO:0005618 | cell wall | 0.000421191 | 23 |
| CC | GO:0030312 | external encapsulating structure | 0.000421191 | 23 |
| CC | GO:0071944 | cell periphery | 0.00247268 | 31 |
| CC | GO:0045259 | proton-transporting ATP synthase complex | 0.006869604 | 7 |
| CC | GO:0048046 | apoplast | 0.007430947 | 13 |
| CC | GO:0045263 | proton-transporting ATP synthase complex, coupling factor F(o) | 0.009743914 | 5 |
| CC | GO:0005576 | extracellular region | 0.012100546 | 13 |
| MF | GO:0001071 | nucleic acid binding transcription factor activity | 5.26352E-09 | 120 |
| MF | GO:0016798 | hydrolase activity, acting on glycosyl bonds | 2.38406E-08 | 101 |
| MF | GO:0043565 | sequence-specific DNA binding | 3.86829E-08 | 67 |
| MF | GO:0004553 | hydrolase activity, hydrolyzing O-glycosyl compounds | 5.31221E-08 | 97 |
| MF | GO:0005509 | calcium ion binding | 3.71546E-06 | 59 |
| MF | GO:0008289 | lipid binding | 4.25001E-05 | 24 |
| MF | GO:0016758 | transferase activity, transferring hexosyl groups | 5.41918E-05 | 97 |
| MF | GO:0004568 | chitinase activity | 5.9126E-05 | 9 |
| MF | GO:0016709 | oxidoreductase activity, acting on paired donors, with incorporation or reduction of molecular oxygen, NAD(P)H as one donor, and incorporation of one atom of oxygen | 0.000528273 | 7 |
| MF | GO:0008964 | phosphoenolpyruvate carboxylase activity | 0.001001957 | 7 |
| MF | GO:0004497 | monooxygenase activity | 0.001528053 | 9 |
| MF | GO:0016209 | antioxidant activity | 0.004146014 | 31 |
| MF | GO:0004611 | phosphoenolpyruvate carboxykinase activity | 0.004490737 | 7 |
| MF | GO:0016157 | sucrose synthase activity | 0.004490737 | 7 |
| MF | GO:0016684 | oxidoreductase activity, acting on peroxide as acceptor | 0.00516584 | 29 |
| MF | GO:0030170 | pyridoxal phosphate binding | 0.005470917 | 25 |
| MF | GO:0070279 | vitamin B6 binding | 0.005470917 | 25 |
| MF | GO:0004674 | protein serine/threonine kinase activity | 0.005630441 | 10 |
| MF | GO:0019842 | vitamin binding | 0.006125869 | 28 |
| MF | GO:0003830 | beta-1,4-mannosylglycoprotein 4-beta-N-acetylglucosaminyltransferase activity | 0.006221978 | 6 |
| MF | GO:0000062 | fatty-acyl-CoA binding | 0.008379335 | 5 |
| MF | GO:0003872 | 6-phosphofructokinase activity | 0.009060724 | 8 |
| MF | GO:0008194 | UDP-glycosyltransferase activity | 0.010748927 | 36 |
| MF | GO:0030246 | carbohydrate binding | 0.011519993 | 32 |
| MF | GO:0004620 | phospholipase activity | 0.01403229 | 6 |
| MF | GO:0016298 | lipase activity | 0.01403229 | 6 |
| MF | GO:1901681 | sulfur compound binding | 0.01598617 | 8 |
| MF | GO:0008375 | acetylglucosaminyltransferase activity | 0.017735733 | 16 |
| MF | GO:0016627 | oxidoreductase activity, acting on the CH-CH group of donors | 0.017735733 | 16 |
| MF | GO:0005516 | calmodulin binding | 0.018021302 | 7 |
| MF | GO:0016778 | diphosphotransferase activity | 0.020510946 | 5 |
| MF | GO:0004601 | peroxidase activity | 0.022806124 | 26 |
| MF | GO:0008509 | anion transmembrane transporter activity | 0.025804376 | 19 |
| MF | GO:0008443 | phosphofructokinase activity | 0.026087572 | 8 |
| MF | GO:0019200 | carbohydrate kinase activity | 0.026087572 | 8 |
| MF | GO:0015116 | sulfate transmembrane transporter activity | 0.027299795 | 9 |
| MF | GO:1901682 | sulfur compound transmembrane transporter activity | 0.027299795 | 9 |
| MF | GO:0008661 | 1-deoxy-D-xylulose-5-phosphate synthase activity | 0.029441551 | 5 |
| MF | GO:0016744 | transferase activity, transferring aldehyde or ketonic groups | 0.029441551 | 5 |
| MF | GO:0016762 | xyloglucan:xyloglucosyl transferase activity | 0.030719824 | 13 |
| MF | GO:0004650 | polygalacturonase activity | 0.032511958 | 8 |
| MF | GO:0008081 | phosphoric diester hydrolase activity | 0.032511958 | 8 |
| MF | GO:0004402 | histone acetyltransferase activity | 0.044229042 | 4 |
| MF | GO:0008883 | glutamyl-tRNA reductase activity | 0.044229042 | 4 |
| MF | GO:0034212 | peptide N-acetyltransferase activity | 0.044229042 | 4 |
| MF | GO:0061733 | peptide-lysine-N-acetyltransferase activity | 0.044229042 | 4 |
| MF | GO:0015035 | protein disulfide oxidoreductase activity | 0.044737461 | 11 |
| MF | GO:0015036 | disulfide oxidoreductase activity | 0.044737461 | 11 |
| MF | GO:0051537 | 2 iron, 2 sulfur cluster binding | 0.045757486 | 6 |

**Table S3** In R3 stage, GJS-8 and XGH shared all DEGs GO enrichment significant pathway.

| Category | ID | Term | pvalue | Gene Number |
| --- | --- | --- | --- | --- |
| BP | GO:0051704 | multi-organism process | 4.43745E-07 | 31 |
| BP | GO:0005984 | disaccharide metabolic process | 2.82975E-06 | 28 |
| BP | GO:0009605 | response to external stimulus | 3.2051E-06 | 14 |
| BP | GO:0009311 | oligosaccharide metabolic process | 5.04155E-06 | 28 |
| BP | GO:0005991 | trehalose metabolic process | 6.78953E-06 | 20 |
| BP | GO:0005992 | trehalose biosynthetic process | 8.87647E-06 | 18 |
| BP | GO:0046351 | disaccharide biosynthetic process | 1.98777E-05 | 18 |
| BP | GO:0009312 | oligosaccharide biosynthetic process | 4.15623E-05 | 18 |
| BP | GO:0009628 | response to abiotic stimulus | 8.86013E-05 | 11 |
| BP | GO:0007186 | G-protein coupled receptor signaling pathway | 0.00014609 | 15 |
| BP | GO:0008037 | cell recognition | 0.000173399 | 22 |
| BP | GO:0009856 | pollination | 0.000173399 | 22 |
| BP | GO:0009875 | pollen-pistil interaction | 0.000173399 | 22 |
| BP | GO:0044706 | multi-multicellular organism process | 0.000173399 | 22 |
| BP | GO:0048544 | recognition of pollen | 0.000173399 | 22 |
| BP | GO:0043207 | response to external biotic stimulus | 0.000246073 | 9 |
| BP | GO:0051707 | response to other organism | 0.000246073 | 9 |
| BP | GO:0098542 | defense response to other organism | 0.000246073 | 9 |
| BP | GO:0006022 | aminoglycan metabolic process | 0.000250527 | 11 |
| BP | GO:0006026 | aminoglycan catabolic process | 0.000250527 | 11 |
| BP | GO:0006030 | chitin metabolic process | 0.000250527 | 11 |
| BP | GO:0006032 | chitin catabolic process | 0.000250527 | 11 |
| BP | GO:0006040 | amino sugar metabolic process | 0.000250527 | 11 |
| BP | GO:0016998 | cell wall macromolecule catabolic process | 0.000250527 | 11 |
| BP | GO:0044036 | cell wall macromolecule metabolic process | 0.000250527 | 11 |
| BP | GO:0046348 | amino sugar catabolic process | 0.000250527 | 11 |
| BP | GO:1901071 | glucosamine-containing compound metabolic process | 0.000250527 | 11 |
| BP | GO:1901072 | glucosamine-containing compound catabolic process | 0.000250527 | 11 |
| BP | GO:0000003 | reproduction | 0.000437138 | 22 |
| BP | GO:0022414 | reproductive process | 0.000437138 | 22 |
| BP | GO:0044702 | single organism reproductive process | 0.000564634 | 21 |
| BP | GO:0072350 | tricarboxylic acid metabolic process | 0.000604987 | 11 |
| BP | GO:1901136 | carbohydrate derivative catabolic process | 0.00068639 | 14 |
| BP | GO:0016567 | protein ubiquitination | 0.000749185 | 26 |
| BP | GO:0032446 | protein modification by small protein conjugation | 0.000749185 | 26 |
| BP | GO:0006820 | anion transport | 0.001399027 | 45 |
| BP | GO:0044262 | cellular carbohydrate metabolic process | 0.001457504 | 75 |
| BP | GO:1901565 | organonitrogen compound catabolic process | 0.002700126 | 21 |
| BP | GO:0015711 | organic anion transport | 0.003330182 | 24 |
| BP | GO:0015849 | organic acid transport | 0.003330182 | 24 |
| BP | GO:0046942 | carboxylic acid transport | 0.003330182 | 24 |
| BP | GO:0009719 | response to endogenous stimulus | 0.00447783 | 24 |
| BP | GO:0009725 | response to hormone | 0.00447783 | 24 |
| BP | GO:0010033 | response to organic substance | 0.00447783 | 24 |
| BP | GO:0071554 | cell wall organization or biogenesis | 0.006104875 | 25 |
| BP | GO:0003333 | amino acid transmembrane transport | 0.00697932 | 16 |
| BP | GO:0006865 | amino acid transport | 0.00697932 | 16 |
| BP | GO:0098656 | anion transmembrane transport | 0.00697932 | 16 |
| BP | GO:1903825 | organic acid transmembrane transport | 0.00697932 | 16 |
| BP | GO:1905039 | carboxylic acid transmembrane transport | 0.00697932 | 16 |
| BP | GO:0048583 | regulation of response to stimulus | 0.007453463 | 9 |
| BP | GO:0006720 | isoprenoid metabolic process | 0.008193976 | 18 |
| BP | GO:0008299 | isoprenoid biosynthetic process | 0.008193976 | 18 |
| BP | GO:0006811 | ion transport | 0.008495406 | 143 |
| BP | GO:0006099 | tricarboxylic acid cycle | 0.009517705 | 8 |
| BP | GO:0006101 | citrate metabolic process | 0.009517705 | 8 |
| BP | GO:0009060 | aerobic respiration | 0.009517705 | 8 |
| BP | GO:0015977 | carbon fixation | 0.009517705 | 8 |
| BP | GO:0030001 | metal ion transport | 0.013925936 | 61 |
| BP | GO:0010035 | response to inorganic substance | 0.015945342 | 10 |
| BP | GO:0042221 | response to chemical | 0.016869196 | 51 |
| BP | GO:0016051 | carbohydrate biosynthetic process | 0.019146132 | 47 |
| BP | GO:0006066 | alcohol metabolic process | 0.021042285 | 9 |
| BP | GO:0019751 | polyol metabolic process | 0.021042285 | 9 |
| BP | GO:0006814 | sodium ion transport | 0.022249526 | 7 |
| BP | GO:0019682 | glyceraldehyde-3-phosphate metabolic process | 0.022249526 | 7 |
| BP | GO:0007154 | cell communication | 0.023459293 | 95 |
| BP | GO:0006575 | cellular modified amino acid metabolic process | 0.035328876 | 10 |
| BP | GO:0034637 | cellular carbohydrate biosynthetic process | 0.038936046 | 42 |
| BP | GO:0044723 | single-organism carbohydrate metabolic process | 0.041540991 | 86 |
| BP | GO:0001101 | response to acid chemical | 0.042771182 | 8 |
| BP | GO:0015980 | energy derivation by oxidation of organic compounds | 0.042771182 | 8 |
| BP | GO:0045333 | cellular respiration | 0.042771182 | 8 |
| BP | GO:1901700 | response to oxygen-containing compound | 0.042771182 | 8 |
| BP | GO:0015696 | ammonium transport | 0.049683518 | 6 |
| CC | GO:0005618 | cell wall | 0.000951096 | 34 |
| CC | GO:0030312 | external encapsulating structure | 0.000951096 | 34 |
| CC | GO:0071944 | cell periphery | 0.002124399 | 50 |
| CC | GO:0005576 | extracellular region | 0.002352508 | 22 |
| CC | GO:0048046 | apoplast | 0.006189523 | 20 |
| CC | GO:0045263 | proton-transporting ATP synthase complex, coupling factor F(o) | 0.032818302 | 6 |
| MF | GO:0043565 | sequence-specific DNA binding | 1.59792E-16 | 125 |
| MF | GO:0001071 | nucleic acid binding transcription factor activity | 1.57503E-09 | 196 |
| MF | GO:0005509 | calcium ion binding | 2.61367E-06 | 94 |
| MF | GO:0030170 | pyridoxal phosphate binding | 0.000215061 | 45 |
| MF | GO:0070279 | vitamin B6 binding | 0.000215061 | 45 |
| MF | GO:0004568 | chitinase activity | 0.000256334 | 11 |
| MF | GO:0008509 | anion transmembrane transporter activity | 0.000471204 | 37 |
| MF | GO:0004842 | ubiquitin-protein transferase activity | 0.000565868 | 30 |
| MF | GO:0019787 | ubiquitin-like protein transferase activity | 0.000565868 | 30 |
| MF | GO:0004553 | hydrolase activity, hydrolyzing O-glycosyl compounds | 0.000767902 | 136 |
| MF | GO:0008514 | organic anion transmembrane transporter activity | 0.000975593 | 19 |
| MF | GO:0008289 | lipid binding | 0.001176939 | 32 |
| MF | GO:0019842 | vitamin binding | 0.00148374 | 48 |
| MF | GO:0016798 | hydrolase activity, acting on glycosyl bonds | 0.001927871 | 138 |
| MF | GO:0008047 | enzyme activator activity | 0.003267029 | 16 |
| MF | GO:0004497 | monooxygenase activity | 0.003589187 | 12 |
| MF | GO:0031683 | G-protein beta/gamma-subunit complex binding | 0.003984374 | 9 |
| MF | GO:0032403 | protein complex binding | 0.003984374 | 9 |
| MF | GO:0016709 | oxidoreductase activity, acting on paired donors, with incorporation or reduction of molecular oxygen, NAD(P)H as one donor, and incorporation of one atom of oxygen | 0.004866499 | 8 |
| MF | GO:0000062 | fatty-acyl-CoA binding | 0.00579862 | 7 |
| MF | GO:0016758 | transferase activity, transferring hexosyl groups | 0.006133047 | 149 |
| MF | GO:0016836 | hydro-lyase activity | 0.006512338 | 19 |
| MF | GO:0005342 | organic acid transmembrane transporter activity | 0.0071533 | 16 |
| MF | GO:0015171 | amino acid transmembrane transporter activity | 0.0071533 | 16 |
| MF | GO:0046943 | carboxylic acid transmembrane transporter activity | 0.0071533 | 16 |
| MF | GO:0005096 | GTPase activator activity | 0.00720914 | 13 |
| MF | GO:0003830 | beta-1,4-mannosylglycoprotein 4-beta-N-acetylglucosaminyltransferase activity | 0.009654582 | 8 |
| MF | GO:0008964 | phosphoenolpyruvate carboxylase activity | 0.009654582 | 8 |
| MF | GO:0004674 | protein serine/threonine kinase activity | 0.012137261 | 14 |
| MF | GO:0004611 | phosphoenolpyruvate carboxykinase activity | 0.013177943 | 9 |
| MF | GO:0016778 | diphosphotransferase activity | 0.022516068 | 7 |
| MF | GO:0005516 | calmodulin binding | 0.024650292 | 10 |
| MF | GO:0001871 | pattern binding | 0.032506618 | 15 |
| MF | GO:0030247 | polysaccharide binding | 0.032506618 | 15 |
| MF | GO:0030695 | GTPase regulator activity | 0.032506618 | 15 |
| MF | GO:0060589 | nucleoside-triphosphatase regulator activity | 0.033217902 | 17 |
| MF | GO:0016831 | carboxy-lyase activity | 0.041766092 | 18 |
| MF | GO:0016157 | sucrose synthase activity | 0.04328975 | 8 |
| MF | GO:0016762 | xyloglucan:xyloglucosyl transferase activity | 0.04984688 | 20 |

**Table S4** Among all DEGs shared by GJS-8 and XGH, the genes related to hormone.

| gene_id | Category | RGJ8_1vsRGJ8_0_log2FoldChange | RGJ8_2vsRGJ8_0_log2FoldChange | RGJ8_3vsRGJ8_0_log2FoldChange | RXGH_1vsRXGH_0_log2FoldChange | RXGH_2vsRXGH_0_log2FoldChange | RXGH_3vsRXGH_0_log2FoldChange |
| --- | --- | --- | --- | --- | --- | --- | --- |
| Tai6.35951 | AUX1 | 1.92 | -0.48 | -1.21 | -1.24 | -2.11 | -5.40 |
| Tai6.1708 | AUX1 | 3.71 | 1.61 | 0.38 | -1.00 | 3.58 | 2.82 |
| Tai6.15639 | AUX1 | -0.40 | -2.37 | -2.22 | -1.22 | -2.97 | -2.12 |
| Tai6.39648 | AUX/IAA | 1.17 | 1.06 | 2.21 | 1.15 | 0.95 | 0.46 |
| Tai6.5262 | AUX/IAA | -1.95 | -1.58 | -0.86 | -0.21 | -1.69 | -0.67 |
| Tai6.27980 | AUX/IAA | 1.95 | 2.18 | 2.90 | 1.19 | 0.49 | 3.60 |
| Tai6.48777 | AUX/IAA | -2.57 | -2.77 | -0.85 | -1.11 | -3.25 | -1.47 |
| Tai6.6991 | AUX/IAA | -0.89 | -1.39 | -3.25 | -3.10 | -4.77 | -3.78 |
| Tai6.22518 | AUX/IAA | 1.12 | -0.63 | 2.76 | 1.53 | 0.16 | 0.72 |
| Tai6.37183 | AUX/IAA | -0.27 | 0.27 | 2.05 | 1.45 | 0.31 | -1.04 |
| Tai6.563 | AUX/IAA | -1.31 | -1.54 | -3.18 | -0.85 | -1.92 | -1.17 |
| Tai6.44587 | ARF | 0.48 | 0.68 | 1.96 | 1.18 | 1.80 | 1.67 |
| Tai6.23113 | ARF | 0.57 | 0.14 | 2.19 | 1.15 | 2.69 | 4.11 |
| Tai6.1333 | CH3 | -0.59 | -2.38 | -3.90 | -2.66 | -5.46 | -4.77 |
| Tai6.27395 | CH3 | 0.71 | -0.78 | -1.58 | -0.76 | -1.32 | -1.54 |
| Tai6.50418 | CH3 | -2.06 | -4.79 | -1.29 | -6.06 | -6.14 | -6.08 |
| Tai6.36369 | CH3 | 1.81 | 0.23 | 0.78 | 2.32 | 1.13 | 0.56 |
| Tai6.34441 | SAUR | -0.52 | -1.65 | -1.98 | -1.84 | -1.70 | -0.35 |
| Tai6.8225 | SAUR | -5.90 | -5.81 | -8.06 | -6.37 | -6.54 | -9.10 |
| Tai6.14155 | SAUR | 6.40 | 6.00 | 4.45 | NA | 4.09 | 5.30 |
| Tai6.35953 | AHP | -1.70 | -1.59 | -1.80 | -1.21 | -1.71 | -3.15 |
| Tai6.10485 | AHP | 1.55 | 3.00 | 3.65 | 4.08 | 5.63 | 3.40 |
| Tai6.9871 | A-ARR | -0.39 | -1.56 | -2.27 | -2.97 | -2.08 | -1.24 |
| Tai6.6182 | A-ARR | -1.73 | -0.34 | -1.68 | -2.33 | -1.08 | -0.90 |
| Tai6.35166 | A-ARR | 0.19 | -1.12 | -2.87 | -2.99 | -1.61 | -0.44 |
| Tai6.2629 | GID2 | -0.71 | -1.28 | -1.80 | -2.23 | -4.23 | -3.13 |
| Tai6.39357 | TF | 2.12 | 1.36 | 2.60 | 2.48 | 1.84 | 2.68 |
| Tai6.30031 | TF | -1.45 | -0.44 | -0.13 | -0.21 | -1.31 | -0.54 |
| Tai6.18308 | PYR/RYL | 1.45 | 0.72 | 2.74 | 1.22 | 2.49 | 1.43 |
| Tai6.22921 | PP2C | -2.68 | -3.94 | -3.75 | -3.15 | -4.32 | -4.27 |
| Tai6.46804 | PP2C | -2.72 | -2.74 | -3.35 | -2.42 | -3.44 | -3.31 |
| Tai6.39214 | PP2C | -1.83 | -1.35 | -0.78 | -1.33 | -2.40 | -2.15 |
| Tai6.11623 | SnRK2 | -2.76 | -3.77 | -4.73 | -1.11 | -3.56 | -5.05 |
| Tai6.48900 | ABF | 0.76 | 1.24 | 1.87 | 2.06 | 2.35 | 1.94 |
| Tai6.12247 | ETR | 1.90 | 2.07 | 2.07 | 0.76 | 1.63 | 3.01 |
| Tai6.10820 | SIMKK | 4.05 | 5.14 | 5.00 | -0.54 | 2.46 | 4.16 |
| Tai6.36354 | EIN2 | 0.62 | 0.94 | 1.18 | 1.08 | 1.61 | 1.37 |
| Tai6.54900 | EBF1/2 | 0.29 | 1.85 | 1.89 | -0.14 | 0.08 | 1.15 |
| Tai6.48960 | EIN3 | 0.85 | 0.79 | 3.16 | 2.44 | 2.01 | 1.88 |
| Tai6.14737 | ERF1/2 | -2.99 | 1.48 | -0.23 | -3.50 | -2.66 | -3.06 |
| Tai6.17891 | ERF1/2 | 1.58 | 2.93 | 1.96 | 1.82 | 2.21 | 0.76 |
| Tai6.18903 | BAK1 | -1.50 | -1.73 | -3.60 | -1.60 | -2.72 | -3.39 |
| Tai6.43006 | CYCD3 | 3.43 | 1.64 | 0.88 | 0.89 | 1.44 | 2.91 |
| Tai6.37902 | CYCD3 | 2.88 | 1.38 | -0.30 | 0.50 | 2.55 | 2.34 |
| Tai6.33033 | JAR1 | -1.54 | -1.09 | -1.98 | -1.08 | -1.38 | -1.17 |
| Tai6.36109 | JAZ | -1.24 | -0.13 | -1.30 | -3.95 | -2.84 | -2.35 |
| Tai6.51127 | JAZ | -1.37 | -1.68 | -3.31 | -1.60 | -3.28 | -2.66 |
| Tai6.16515 | JAZ | -2.21 | -2.03 | -3.33 | -2.63 | -3.64 | -4.40 |
| Tai6.11161 | JAZ | -3.72 | -2.40 | -3.75 | -6.00 | -5.69 | -5.08 |
| Tai6.30157 | JAZ | -2.71 | -1.52 | -3.10 | -4.28 | -2.95 | -3.06 |
| Tai6.32738 | NPR1 | 2.09 | 0.17 | 0.78 | 1.54 | 0.78 | 1.19 |
| Tai6.52704 | NPR1 | 1.49 | 0.69 | 1.96 | 2.36 | 1.59 | 2.19 |
| Tai6.3711 | TGA | -1.48 | -1.82 | -1.43 | -1.14 | -1.17 | -1.88 |
| Tai6.43035 | TGA | -1.09 | -0.38 | -0.62 | -1.25 | -1.55 | -1.82 |
| Tai6.10133 | PR-1 | -4.35 | -9.46 | -9.65 | -8.07 | 1.08 | -8.09 |
| Tai6.12476 | PR-1 | -1.78 | -6.68 | -8.64 | -6.01 | 1.63 | -4.26 |
| Tai6.29286 | PR-1 | -1.56 | -4.80 | -3.36 | -2.66 | 1.07 | -5.28 |
| Tai6.34570 | PR-1 | 3.00 | 0.39 | -4.33 | -1.66 | 1.08 | 2.61 |

**Table S5** Among all DEGs shared by GJS-8 and XGH, the genes related to MAPK signaling.

| Genes | RGJ8_1vsRGJ8_0_log2FoldChange | RGJ8_2vsRGJ8_0_log2FoldChange | RGJ8_3vsRGJ8_0_log2FoldChange | RXGH_1vsRXGH_0_log2FoldChange | RXGH_2vsRXGH_0_log2FoldChange | RXGH_3vsRXGH_0_log2FoldChange | Description |
| --- | --- | --- | --- | --- | --- | --- | --- |
| Tai6.44720 | 1.46 | 1.04 | 0.78 | 1.82 | 1.38 | 1.78 | MAP7 |
| Tai6.30659 | -0.87 | -1.41 | -3.00 | -1.27 | -1.61 | -2.42 | MAPK4 |
| Tai6.51504 | 0.13 | -0.68 | -1.42 | 0.07 | -1.07 | -1.44 | MAP |
| Tai6.19616 | -0.11 | -2.11 | -4.51 | -1.35 | -4.26 | -2.58 | mitogen-activated protein kinase |
| Tai6.19617 | 0.26 | -1.44 | -2.83 | -1.00 | -2.69 | 0.55 | mitogen-activated protein kinase |
| Tai6.3505 | -0.33 | -0.49 | -1.69 | -0.69 | -0.84 | -1.36 | MMK2-like |
| Tai6.38130 | -1.62 | -1.38 | -2.23 | -1.89 | -2.82 | -4.25 | mitogen-activated protein kinase |
| Tai6.51134 | 1.21 | 1.43 | 2.32 | 1.54 | 1.81 | 2.31 | mitogen-activated protein kinase |
| Tai6.10820 | 4.05 | 5.14 | 5.00 | -0.54 | 2.46 | 4.16 | mitogen-activated protein kinase |
| Tai6.38233 | -2.28 | -2.23 | -3.58 | -4.29 | -3.11 | -4.04 | mitogen-activated protein kinase |
| Tai6.14244 | -1.46 | -2.03 | -4.45 | -3.75 | -5.74 | -2.76 | mitogen-activated protein kinase |
| Tai6.8872 | -1.54 | -0.90 | -2.26 | -3.57 | -2.09 | -0.72 | mitogen-activated protein kinase |
| Tai6.17036 | -1.53 | -1.17 | -2.24 | -3.60 | -1.93 | -0.41 | mitogen-activated protein kinase |
| Tai6.47914 | -1.32 | -0.77 | -1.96 | -1.26 | -2.17 | -3.46 | mitogen-activated protein kinase |
| Tai6.53051 | -1.30 | -0.29 | -1.24 | -2.03 | -2.03 | -1.32 | mitogen-activated protein kinase |
| Tai6.46853 | -1.42 | -2.08 | -3.10 | -2.83 | -3.83 | -3.86 | mitogen-activated protein kinase |
| Tai6.49981 | -1.55 | -2.55 | -3.57 | -2.79 | -3.98 | -5.33 | mitogen-activated protein kinase |
| Tai6.34802 | -1.20 | -1.76 | -1.92 | -0.96 | -1.68 | -2.27 | mitogen-activated protein kinase |
| Tai6.28566 | -1.61 | -2.43 | -4.74 | -6.12 | -5.00 | -4.22 | mitogen-activated protein kinase |
| Tai6.52764 | -1.17 | -1.05 | -1.90 | -0.33 | -1.85 | -2.78 | mitogen-activated protein kinase |
| Tai6.24167 | -1.01 | 0.23 | -0.63 | -2.13 | -1.63 | -0.72 | mitogen-activated protein kinase |
| Tai6.49262 | -1.16 | -2.10 | -3.65 | -7.04 | -4.46 | -3.79 | mitogen-activated protein kinase |
| Tai6.53239 | 0.80 | 1.68 | 3.21 | -0.15 | 0.99 | 2.06 | mitogen-activated protein kinase |
| Tai6.7760 | 0.83 | 1.24 | 2.70 | -0.16 | 0.70 | 1.90 | mitogen-activated protein kinase |
| Tai6.16191 | -0.71 | -1.20 | -1.54 | -1.17 | -1.82 | -2.17 | mitogen-activated protein kinase |
| Tai6.19794 | -0.93 | -1.26 | -1.75 | -0.65 | -1.18 | -1.33 | mitogen-activated protein kinase |
| Tai6.9123 | 0.81 | 1.16 | 2.67 | -0.51 | 0.40 | 1.44 | mitogen-activated protein kinase |
| Tai6.10190 | 0.24 | -1.12 | -2.69 | 0.01 | -2.11 | -2.12 | mitogen-activated protein kinase |
| Tai6.28952 | 0.08 | -0.87 | -2.32 | -1.99 | -1.48 | -0.81 | mitogen-activated protein kinase |
| Tai6.40499 | 0.38 | -0.88 | -2.31 | 0.33 | -1.88 | -2.34 | mitogen-activated protein kinase |
| Tai6.2216 | -0.71 | -0.47 | -2.36 | -0.39 | -1.50 | -3.91 | mitogen-activated protein kinase |
| Tai6.30899 | -0.42 | -0.54 | -2.11 | -0.53 | -1.80 | -3.14 | mitogen-activated protein kinase |
| Tai6.19654 | -0.87 | -1.11 | -2.14 | -2.27 | -2.82 | -2.93 | mitogen-activated protein kinase |
| Tai6.3542 | -1.06 | -2.42 | -3.53 | -4.57 | -3.55 | -3.86 | mitogen-activated protein kinase |
| Tai6.53199 | -1.74 | -1.38 | -2.38 | 2.35 | 1.37 | 0.09 | mitogen-activated protein kinase |
| Tai6.41095 | -0.21 | -0.82 | -1.10 | -0.33 | -0.42 | -1.21 | mitogen-activated protein kinase |
| Tai6.4327 | 0.12 | 0.15 | 1.08 | 1.86 | 2.16 | 1.07 | mitogen-activated protein kinase |
| Tai6.4257 | -0.58 | -0.79 | -1.12 | -0.54 | -0.83 | -1.30 | mitogen-activated protein kinase |
| Tai6.7143 | -1.02 | -1.00 | -1.02 | -0.48 | -1.05 | -1.07 | mitogen-activated protein kinase |
| Tai6.54983 | -1.42 | -1.55 | -3.74 | -3.41 | -5.21 | -2.50 | mitogen-activated protein kinase |
| Tai6.2215 | -1.10 | -1.56 | -3.18 | -0.99 | -2.64 | -3.66 | mitogen-activated protein kinase |
| Tai6.19806 | -1.37 | -2.00 | -3.03 | -5.99 | -4.40 | -3.49 | mitogen-activated protein kinase |
| Tai6.11961 | 0.45 | 2.64 | 2.90 | -1.28 | -0.48 | -1.72 | mitogen-activated protein kinase |
| Tai6.11960 | 0.62 | 2.65 | 2.90 | -1.34 | -0.32 | -1.80 | mitogen-activated protein kinase |
| Tai6.33803 | -0.86 | -0.54 | -1.27 | -0.29 | -1.08 | -2.27 | mitogen-activated protein kinase |

**Table S6** Among all DEGs shared by GJS-8 and XGH, the genes related to calcium signaling.

| Gene_ID | RGJ8_1vsRGJ8_0_log2FoldChange | RGJ8_2vsRGJ8_0_log2FoldChange | RGJ8_3vsRGJ8_0_log2FoldChange | RXGH_1vsRXGH_0_log2FoldChange | RXGH_2vsRXGH_0_log2FoldChange | RXGH_3vsRXGH_0_log2FoldChange | Description |
| --- | --- | --- | --- | --- | --- | --- | --- |
| Tai6.6605 | -2.49 | -1.58 | -2.79 | -4.43 | -4.03 | -3.92 | CBL |
| Tai6.38872 | -2.15 | -1.39 | -2.63 | -4.74 | -4.62 | -3.89 | CBL |
| Tai6.2752 | 1.73 | 3.08 | 2.96 | 0.53 | 1.79 | 2.04 | CBL |
| Tai6.20325 | -2.05 | -0.90 | -2.58 | -2.71 | -2.16 | -2.87 | CBL |
| Tai6.23530 | -2.03 | -1.51 | -2.57 | -3.93 | -3.82 | -3.81 | CBL |
| Tai6.7769 | -1.75 | -0.47 | -1.89 | -2.81 | -2.24 | -2.99 | CBL |
| Tai6.34182 | -1.44 | -0.63 | -0.94 | -0.50 | -1.08 | -1.54 | CBL |
| Tai6.19154 | -1.38 | -1.06 | -1.56 | -0.91 | -1.49 | -1.72 | CBL |
| Tai6.12844 | -1.34 | -0.38 | -0.30 | -1.40 | -1.89 | -2.24 | CBL |
| Tai6.20643 | -1.17 | -0.33 | -0.13 | -1.19 | -1.80 | -1.95 | CBL |
| Tai6.36976 | 1.59 | 3.30 | 4.52 | 1.12 | 2.08 | 2.43 | CBL |
| Tai6.42208 | 1.21 | 2.83 | 4.21 | 1.70 | 1.80 | 2.42 | CBL |
| Tai6.41392 | -2.54 | -3.96 | -2.73 | -1.20 | -2.05 | -6.45 | CBL |
| Tai6.19483 | 0.64 | 1.90 | 3.35 | 1.10 | 3.21 | 4.29 | CBL |
| Tai6.2755 | 0.08 | 1.08 | 1.21 | -1.19 | -0.10 | 0.33 | CBL |
| Tai6.48403 | 0.82 | 0.81 | 4.33 | 1.08 | 2.14 | 1.87 | CBL |
| Tai6.31037 | 0.08 | 0.96 | 1.59 | -2.55 | -2.38 | -2.02 | CBL |
| Tai6.50374 | 0.09 | 0.60 | 1.45 | 0.12 | 0.77 | 1.07 | CBL |
| Tai6.21149 | 0.09 | 0.92 | 1.48 | -2.15 | -2.07 | -1.96 | CBL |
| Tai6.48830 | 0.62 | 0.12 | 1.50 | 2.00 | 1.61 | 1.14 | CBL |
| Tai6.19578 | -0.38 | 0.50 | 1.35 | 0.98 | 2.00 | 2.40 | CBL |
| Tai6.2754 | 0.58 | 2.37 | 3.13 | 3.76 | 2.45 | -0.93 | CBL |
| Tai6.33139 | -0.41 | -0.65 | -1.14 | -0.32 | -0.93 | -1.87 | CBL |
| Tai6.51594 | -1.10 | 0.13 | -0.95 | -1.83 | -1.57 | -2.33 | CBL |
| Tai6.42218 | 1.11 | 1.32 | -0.14 | -1.86 | -0.40 | -0.35 | CBL |
| Tai6.5552 | 0.91 | 1.43 | 1.86 | 1.74 | 1.47 | 1.16 | CBL |
| Tai6.21349 | -4.57 | 0.59 | -1.28 | -3.15 | -2.21 | -1.95 | CDPK |
| Tai6.9544 | -3.06 | -0.16 | -2.16 | -4.44 | -3.26 | -2.78 | CDPK |
| Tai6.17447 | -1.74 | -1.05 | -1.36 | -1.52 | -2.32 | -1.95 | CDPK |
| Tai6.17976 | 1.91 | 2.20 | 1.67 | 0.94 | 1.08 | 1.44 | CDPK |
| Tai6.16670 | 2.24 | 2.61 | 2.22 | 1.95 | 2.64 | 3.09 | CDPK |
| Tai6.11088 | -1.70 | -0.63 | -1.11 | -1.11 | -1.84 | -1.36 | CDPK |
| Tai6.14345 | -1.48 | -0.48 | -0.98 | -1.11 | -1.58 | -1.20 | CDPK |
| Tai6.7421 | -1.65 | -1.52 | -2.03 | -1.66 | -2.12 | -2.79 | CDPK |
| Tai6.25966 | -1.41 | -1.22 | -1.65 | -1.97 | -1.95 | -3.35 | CDPK |
| Tai6.7422 | -1.49 | -0.65 | -0.97 | -1.84 | -1.65 | -2.08 | CDPK |
| Tai6.38225 | 1.88 | 1.31 | 1.92 | 1.23 | 2.70 | 1.86 | CDPK |
| Tai6.40383 | -1.61 | -1.26 | -2.20 | -1.53 | -2.02 | -2.59 | CDPK |
| Tai6.10163 | -1.23 | -0.36 | -1.41 | -1.24 | -1.74 | -2.87 | CDPK |
| Tai6.51775 | -1.29 | -1.91 | -2.81 | -1.54 | -2.37 | -3.06 | CDPK |
| Tai6.44150 | 1.06 | 0.77 | 0.12 | 1.07 | 0.95 | 0.94 | CDPK |
| Tai6.51011 | -1.26 | -0.97 | -1.95 | -1.05 | -1.90 | -2.45 | CDPK |
| Tai6.4383 | 1.51 | 0.62 | 1.40 | 1.20 | 1.60 | 2.06 | CDPK |
| Tai6.11090 | -1.08 | 0.06 | -0.13 | -0.77 | -1.21 | -0.92 | CDPK |
| Tai6.49809 | 1.22 | 0.89 | -0.86 | 0.14 | -0.76 | -2.34 | CDPK |
| Tai6.42949 | -1.11 | -0.77 | -1.06 | -1.88 | -1.62 | -2.59 | CDPK |
| Tai6.49139 | 1.27 | 1.50 | 0.85 | 1.27 | 1.39 | 1.38 | CDPK |
| Tai6.15046 | -0.29 | -1.97 | -2.05 | 0.11 | -2.79 | -2.82 | CDPK |
| Tai6.1820 | -0.22 | -1.56 | -1.64 | 0.12 | -2.34 | -2.89 | CDPK |
| Tai6.1784 | -0.94 | -1.35 | -2.67 | -1.51 | -2.49 | -2.99 | CDPK |
| Tai6.2154 | -0.61 | -1.21 | -2.14 | -2.06 | -2.45 | -3.05 | CDPK |
| Tai6.5215 | -1.02 | -1.73 | -2.44 | -2.21 | -2.38 | -2.55 | CDPK |
| Tai6.756 | 0.93 | 1.13 | 1.07 | 1.05 | 1.23 | 1.44 | CDPK |
| Tai6.43979 | 0.80 | 1.06 | 1.06 | 0.43 | 0.64 | 1.14 | CDPK |
| Tai6.24479 | -0.66 | -1.03 | -1.82 | -2.04 | -2.51 | -2.95 | CDPK |
| Tai6.5956 | -0.30 | -0.66 | -1.49 | -0.83 | -2.39 | -2.14 | CDPK |
| Tai6.53380 | 0.00 | -1.06 | -1.16 | -0.48 | -1.29 | -1.46 | CDPK |
| Tai6.37218 | -0.43 | -0.03 | -1.34 | 0.01 | -0.75 | -1.51 | CDPK |
| Tai6.33112 | 1.21 | 1.47 | 1.78 | 3.05 | 2.86 | 3.09 | CDPK |
| Tai6.46800 | 0.60 | 0.19 | -1.55 | -0.14 | -1.11 | -1.95 | CDPK |
| Tai6.23393 | 0.89 | 0.37 | -1.50 | -0.35 | -1.27 | -2.03 | CDPK |
| Tai6.10206 | -0.06 | -0.14 | -1.06 | -1.24 | -1.39 | -2.29 | CDPK |
| Tai6.11470 | -3.05 | -4.73 | -6.35 | -5.06 | -5.24 | -5.84 | CaM |
| Tai6.30253 | -3.17 | -4.49 | -6.39 | -5.33 | -5.16 | -5.25 | CaM |
| Tai6.29963 | -3.06 | -4.60 | -7.34 | -6.72 | -6.81 | -4.97 | CaM |
| Tai6.33565 | -1.99 | -3.54 | -4.64 | 1.22 | -2.53 | -3.69 | CaM |
| Tai6.11296 | -3.03 | -4.78 | -8.70 | -4.83 | -4.18 | -5.31 | CaM |
| Tai6.1445 | -1.94 | -1.74 | -1.97 | -1.22 | -1.64 | -1.87 | CaM |
| Tai6.5851 | -1.48 | -0.88 | -1.13 | -1.87 | -2.44 | -1.70 | CaM |
| Tai6.47238 | -1.59 | -0.63 | -0.82 | -0.81 | -0.88 | -1.07 | CaM |
| Tai6.4448 | -1.42 | -1.58 | -3.06 | -0.80 | -2.69 | -3.07 | CaM |
| Tai6.46641 | -1.47 | -1.02 | -1.37 | -1.91 | -2.21 | -1.45 | CaM |
| Tai6.7137 | -1.77 | -1.27 | -1.67 | -0.70 | -2.23 | -3.54 | CaM |
| Tai6.18038 | -1.38 | -1.37 | -1.67 | -1.14 | -1.46 | -1.54 | CaM |
| Tai6.46291 | 1.40 | 0.62 | 0.02 | 1.19 | -0.33 | 0.48 | CaM |
| Tai6.52953 | 1.63 | -0.04 | -0.58 | 1.46 | 0.67 | 0.81 | CaM |
| Tai6.14544 | -1.11 | -1.14 | -1.26 | -0.51 | -2.61 | -4.51 | CaM |
| Tai6.44997 | -1.13 | -2.08 | -2.65 | -0.20 | -2.06 | -4.13 | CaM |
| Tai6.25137 | -1.20 | -0.77 | -1.23 | -0.35 | -0.85 | -1.54 | CaM |
| Tai6.20710 | -1.35 | -1.82 | -2.42 | 0.19 | -2.00 | -3.85 | CaM |
| Tai6.3553 | -1.24 | -1.29 | -1.36 | -1.92 | -2.56 | -1.75 | CaM |
| Tai6.55027 | 1.20 | 0.33 | -1.20 | 0.67 | -2.01 | -1.68 | CaM |
| Tai6.39391 | -1.06 | -0.69 | -0.93 | -0.94 | -1.46 | -1.30 | CaM |
| Tai6.27139 | -1.03 | -0.64 | -1.21 | -0.43 | -1.05 | -1.17 | CaM |
| Tai6.4447 | -1.18 | -1.78 | -3.32 | -0.42 | -3.06 | -0.72 | CaM |
| Tai6.20392 | -1.06 | -0.14 | -1.23 | 0.06 | -1.72 | -2.86 | CaM |
| Tai6.50689 | -0.77 | -2.19 | -2.95 | 0.13 | -0.97 | -1.28 | CaM |
| novel.317 | -1.87 | -4.56 | -7.31 | -2.66 | -0.97 | -4.10 | CaM |
| Tai6.31196 | -0.42 | -1.69 | -2.91 | -0.63 | -2.22 | -0.76 | CaM |
| Tai6.48440 | -0.97 | -1.49 | -2.99 | -0.34 | -3.41 | -1.16 | CaM |
| Tai6.1365 | 0.09 | -1.16 | -2.19 | -0.92 | -2.12 | -2.84 | CaM |
| Tai6.37697 | -0.63 | -1.14 | -2.12 | -0.37 | -1.94 | -3.27 | CaM |
| Tai6.20946 | -0.42 | -0.97 | -2.68 | -1.65 | -2.13 | -2.20 | CaM |
| Tai6.18653 | -0.62 | -0.81 | -3.23 | -2.64 | -2.88 | -3.31 | CaM |
| Tai6.47012 | -0.32 | -0.93 | -2.41 | -1.23 | -2.09 | -2.22 | CaM |
| Tai6.7821 | 0.18 | 0.61 | -1.85 | -2.94 | -1.70 | -2.97 | CaM |
| Tai6.33806 | -0.60 | -0.72 | -1.79 | -0.20 | -1.51 | -3.01 | CaM |
| Tai6.17457 | 0.30 | 0.06 | 1.75 | 1.30 | 1.77 | 1.80 | CaM |
| Tai6.21012 | 0.77 | 1.44 | 2.33 | 1.04 | -0.03 | -1.61 | CaM |
| Tai6.46076 | -0.20 | 0.59 | 1.33 | 1.03 | 0.93 | 0.76 | CaM |
| Tai6.7960 | -0.96 | -0.64 | -1.28 | -0.70 | -1.02 | -1.16 | CaM |
| Tai6.41803 | 0.14 | -0.93 | -2.26 | -0.68 | -1.75 | -2.26 | CaM |
| Tai6.42152 | 0.64 | -1.40 | -1.92 | -1.99 | -2.44 | -2.12 | CaM |
| Tai6.27541 | 0.02 | 1.08 | 1.19 | 1.02 | 0.86 | 0.67 | CaM |
| Tai6.36207 | -0.24 | -0.39 | -1.08 | -0.97 | -0.95 | -1.28 | CaM |
| Tai6.4407 | 0.50 | 0.99 | 1.12 | 1.38 | 1.00 | 0.25 | CaM |
| Tai6.4036 | -0.49 | -1.57 | -1.70 | -0.58 | -1.41 | -1.28 | CaM |
| Tai6.32686 | -3.43 | -1.88 | -3.36 | -2.46 | -4.10 | -4.86 | CBP |
| Tai6.31470 | -2.69 | -2.91 | -3.02 | -2.34 | -1.96 | -4.35 | CBP |
| Tai6.10562 | -2.21 | -5.27 | -8.52 | -4.05 | -6.58 | -4.33 | CBP |
| Tai6.20725 | -2.37 | -4.93 | -7.91 | -4.54 | -7.11 | -4.96 | CBP |
| Tai6.987 | -3.45 | -4.69 | -3.97 | -2.09 | -3.27 | -4.76 | CBP |
| Tai6.18823 | -2.85 | -2.94 | -2.92 | -2.56 | -2.39 | -3.52 | CBP |
| Tai6.1456 | -1.90 | -2.99 | -4.87 | -4.08 | -4.25 | -5.81 | CBP |
| Tai6.37392 | -1.63 | -3.48 | -5.86 | -2.93 | -4.43 | -5.03 | CBP |
| Tai6.27505 | -1.64 | -3.32 | -6.04 | -3.41 | -5.30 | -4.95 | CBP |
| Tai6.18191 | -1.56 | -3.45 | -5.80 | -3.46 | -4.18 | -4.20 | CBP |
| Tai6.35120 | -1.62 | -4.30 | -7.05 | -4.00 | -6.88 | -4.12 | CBP |
| Tai6.46371 | -1.88 | -4.44 | -7.33 | -3.79 | -9.73 | -3.98 | CBP |
| Tai6.46899 | -1.55 | -5.74 | -7.03 | -2.50 | -5.14 | -3.30 | CBP |
| Tai6.34669 | 1.20 | 0.89 | -0.21 | -0.20 | 1.15 | 1.67 | CBP |
| Tai6.15352 | 1.14 | 0.45 | -0.44 | -0.19 | 0.72 | 1.71 | CBP |
| Tai6.15353 | 1.31 | 0.85 | -0.18 | 0.42 | 0.78 | 1.48 | CBP |
| Tai6.4519 | -1.14 | -0.76 | -0.50 | -0.07 | -0.99 | -1.32 | CBP |
| Tai6.25748 | -1.15 | -1.46 | -1.78 | -0.33 | -1.97 | -3.47 | CBP |
| Tai6.6978 | -0.73 | -2.70 | -3.54 | -0.61 | -1.68 | -2.16 | CBP |
| Tai6.35642 | -0.88 | -3.61 | -6.46 | -4.47 | -6.34 | -2.88 | CBP |
| Tai6.8982 | 0.71 | -2.34 | -4.65 | -1.83 | -3.54 | -0.80 | CBP |
| Tai6.26462 | 0.40 | -2.44 | -5.29 | -1.98 | -3.49 | -1.24 | CBP |
| Tai6.4029 | -0.20 | -2.21 | -3.79 | -3.20 | -5.32 | -1.16 | CBP |
| Tai6.37435 | -0.95 | -1.54 | -2.12 | -0.60 | -1.04 | -1.69 | CBP |
| Tai6.4886 | 0.46 | 1.58 | -0.13 | 0.03 | -1.06 | -0.46 | CBP |
| Tai6.32161 | -0.02 | -1.95 | -4.41 | -1.40 | -4.81 | -3.38 | CBP |
| Tai6.2250 | -0.23 | -2.45 | -4.46 | -2.34 | -3.32 | -4.36 | CBP |
| Tai6.4398 | 0.37 | 1.24 | -0.46 | 0.20 | -1.94 | -1.18 | CBP |
| Tai6.7922 | 0.73 | -1.82 | -4.10 | -1.51 | -4.36 | -1.20 | CBP |
| Tai6.27602 | -0.92 | -1.48 | -1.74 | -0.56 | -2.99 | -4.44 | CBP |
| Tai6.31278 | -0.99 | -1.34 | -1.79 | -0.85 | -3.36 | -4.38 | CBP |
| Tai6.30794 | -0.46 | -1.09 | -3.29 | -2.78 | -4.76 | -2.14 | CBP |
| Tai6.20495 | -0.75 | -0.93 | -3.32 | -3.53 | -1.59 | -3.56 | CBP |
| Tai6.25703 | -1.46 | -1.04 | -4.09 | -2.73 | -1.63 | -3.18 | CBP |
| Tai6.31590 | -0.05 | -0.38 | -1.51 | -1.26 | -1.83 | -1.09 | CBP |
| Tai6.25033 | -0.20 | -0.45 | -1.40 | -0.80 | -1.07 | -1.11 | CBP |
| Tai6.54113 | 0.36 | 0.23 | -1.94 | 0.05 | -2.55 | -0.16 | CBP |
| Tai6.6416 | 0.41 | 0.51 | -1.70 | 0.03 | -2.25 | -0.16 | CBP |
| Tai6.47060 | -0.01 | -0.94 | -1.16 | -1.14 | -2.63 | -2.28 | CBP |
| Tai6.40903 | 0.34 | 0.66 | -1.63 | -0.13 | -2.58 | -0.18 | CBP |

**Table S7** Among all DEGs shared by GJS-8 and XGH, the genes related to phospholipid signaling.

| Genes | RGJ8_1vsRGJ8_0_log2FoldChange | RGJ8_2vsRGJ8_0_log2FoldChange | RGJ8_3vsRGJ8_0_log2FoldChange | RXGH_1vsRXGH_0_log2FoldChange | RXGH_2vsRXGH_0_log2FoldChange | RXGH_3vsRXGH_0_log2FoldChange |
| --- | --- | --- | --- | --- | --- | --- |
| Tai6.12367 | -3.45 | -0.87 | -3.24 | -1.82 | -1.83 | -2.11 |
| Tai6.44650 | 2.54 | 1.85 | 4.14 | 2.63 | 2.88 | 3.32 |
| Tai6.7440 | 0.82 | 1.80 | 2.33 | 0.46 | 0.96 | 1.60 |
| Tai6.16114 | 0.27 | 1.55 | 2.01 | 0.80 | 1.54 | 0.63 |
| Tai6.7439 | 0.57 | 1.13 | 1.67 | 0.15 | 0.17 | 1.15 |
| Tai6.48374 | -0.48 | 1.60 | 3.41 | -1.70 | -0.29 | 0.31 |
| Tai6.17546 | 2.31 | 1.06 | 3.65 | 3.64 | 3.89 | 4.28 |
| Tai6.37762 | 0.28 | 1.15 | 1.55 | 1.04 | 0.94 | 0.96 |
| Tai6.40141 | 0.28 | 0.96 | 1.28 | 0.46 | 1.18 | 0.05 |
| Tai6.4244 | 0.35 | 0.73 | 1.10 | 1.24 | 1.24 | 1.03 |
| Tai6.26026 | 0.44 | 1.13 | 1.14 | 0.34 | 1.03 | 1.04 |
| Tai6.21792 | 3.49 | 3.60 | 1.58 | 2.14 | 3.57 | 4.29 |
| Tai6.4424 | 4.01 | 3.60 | 1.79 | 2.01 | 2.97 | 3.56 |
| Tai6.27012 | 4.36 | 4.04 | 2.33 | 2.17 | 3.09 | 3.72 |
| Tai6.17736 | 1.37 | 1.70 | 1.33 | 1.28 | 1.74 | 1.60 |
| Tai6.37746 | -2.98 | -3.58 | -3.70 | -2.15 | -1.38 | -2.36 |
| Tai6.51908 | -1.45 | -3.06 | -3.99 | -1.74 | -2.62 | -4.34 |
| Tai6.4640 | -3.20 | -1.31 | -3.50 | -3.64 | -3.67 | -4.80 |
| Tai6.6882 | 0.13 | 1.04 | 2.66 | 1.10 | 0.77 | 0.87 |
| Tai6.50536 | 1.04 | 1.04 | 1.66 | 1.36 | 1.77 | 1.62 |
| Tai6.1016 | 1.00 | 0.96 | 1.70 | 1.38 | 1.62 | 1.94 |
| Tai6.32349 | -3.66 | -3.97 | -5.66 | -3.64 | -3.77 | -3.65 |

**Table S8** Among all DEGs shared by GJS-8 and XGH, the genes related to photoperiod.

| Gene_ID | RGJ8_1vsRGJ8_0_log2FoldChange | RGJ8_2vsRGJ8_0_log2FoldChange | RGJ8_3vsRGJ8_0_log2FoldChange | RXGH_1vsRXGH_0_log2FoldChange | RXGH_2vsRXGH_0_log2FoldChange | RXGH_3vsRXGH_0_log2FoldChange | Description |
| --- | --- | --- | --- | --- | --- | --- | --- |
| Tai6.48131 | -2.05 | -0.97 | -1.22 | -2.05 | -1.72 | -3.53 | GATA transcription factor |
| Tai6.15631 | -1.77 | -0.72 | -1.24 | -1.71 | -1.54 | -2.43 | GATA transcription factor |
| Tai6.15633 | -1.53 | -0.37 | -0.79 | -1.72 | -1.30 | -2.51 | GATA transcription factor |
| Tai6.52929 | 1.25 | -0.13 | -1.58 | 1.60 | -0.47 | -2.15 | GATA transcription factor |
| Tai6.53733 | -0.56 | -3.60 | -4.50 | -1.47 | -3.96 | -3.65 | GATA transcription factor |
| Tai6.11865 | -0.41 | -3.33 | -4.15 | -1.12 | -3.68 | -3.65 | GATA transcription factor |
| Tai6.11314 | -0.08 | -2.90 | -3.87 | -0.03 | -2.71 | -2.68 | GATA transcription factor |
| Tai6.27468 | 0.88 | 1.42 | 1.24 | 1.04 | 1.19 | 1.20 | GATA transcription factor |
| Tai6.9273 | 0.62 | -1.31 | -2.09 | 0.57 | -1.54 | -2.60 | GATA transcription factor |
| Tai6.45006 | 0.95 | -0.89 | -1.49 | 0.82 | -0.98 | -2.08 | GATA transcription factor |
| Tai6.7179 | 0.38 | -0.44 | -1.07 | -0.31 | -0.07 | -1.76 | GATA transcription factor |
| Tai6.35486 | -0.80 | -1.68 | -1.61 | -1.08 | -1.08 | -1.37 | GATA transcription factor |
| Tai6.34659 | -0.49 | -2.01 | -1.01 | -0.25 | -1.25 | -1.18 | GATA transcription factor |
| Tai6.5144 | -0.33 | -1.17 | -1.44 | -0.42 | -1.63 | -1.29 | GATA transcription factor |
| Tai6.18322 | 5.42 | 7.45 | 5.95 | 0.26 | 7.28 | 8.31 | LOB domain-containing protein |
| Tai6.27900 | 8.32 | 9.31 | 9.09 | 0.29 | 4.84 | 6.70 | LOB domain-containing protein |
| Tai6.27010 | 8.08 | 9.64 | 8.86 | NA | 9.99 | 10.91 | LOB domain-containing protein |
| Tai6.7597 | 1.83 | 1.64 | 3.20 | 1.13 | -1.03 | -0.98 | LOB domain-containing protein |
| Tai6.9448 | 1.54 | 1.58 | 0.91 | -0.40 | -0.31 | 1.32 | LOB domain-containing protein |
| Tai6.14685 | 1.85 | 3.14 | 1.61 | -2.95 | -3.68 | -0.46 | LOB domain-containing protein |
| Tai6.47563 | 1.74 | 2.99 | 1.17 | -2.01 | -2.35 | -0.83 | LOB domain-containing protein |
| Tai6.48572 | -0.55 | -2.27 | -2.76 | -1.20 | -0.53 | -3.71 | LOB domain-containing protein |
| Tai6.48903 | -0.34 | -2.05 | -2.69 | -0.19 | -0.34 | -4.00 | LOB domain-containing protein |
| Tai6.43082 | 0.96 | -2.34 | -3.82 | -2.80 | -1.72 | -2.58 | LOB domain-containing protein |
| Tai6.43055 | 1.04 | 1.79 | -1.52 | -5.89 | -0.96 | 2.52 | LOB domain-containing protein |
| Tai6.48336 | 1.46 | 0.45 | 2.19 | 2.20 | 2.91 | 1.57 | LOB domain-containing protein |
| Tai6.10194 | 1.16 | 1.10 | 2.82 | 1.23 | 0.88 | 1.13 | Phototropin protein genes |
| Tai6.43604 | 1.29 | 1.50 | 3.18 | 1.61 | 1.61 | 1.62 | Phototropin protein genes |
| Tai6.21811 | 1.41 | 0.92 | 2.73 | 1.32 | 1.08 | 1.34 | Phototropin protein genes |
| Tai6.10801 | -0.69 | -2.77 | -1.53 | 1.50 | 0.09 | -1.55 | Phototropin protein genes |
| Tai6.23046 | 2.76 | 3.74 | 2.53 | -0.31 | 1.04 | 2.01 | Phototropin protein genes |
| Tai6.3107 | 3.33 | 4.20 | 5.56 | 1.82 | 4.33 | 4.07 | CONSTANS-like |
| Tai6.16421 | 3.25 | 4.15 | 5.49 | 2.47 | 4.64 | 4.39 | CONSTANS-like |
| Tai6.35600 | 2.31 | 1.88 | -0.30 | 2.00 | 3.37 | 2.77 | CONSTANS-like |
| Tai6.45783 | 2.29 | 1.93 | -0.15 | 1.37 | 2.02 | 1.51 | CONSTANS-like |
| Tai6.41474 | -1.66 | 0.33 | 0.62 | 0.14 | 2.05 | 2.58 | CONSTANS-like |
| Tai6.46416 | 1.73 | 1.64 | -0.39 | 1.71 | 3.15 | 2.57 | CONSTANS-like |
| Tai6.41327 | -0.84 | -1.43 | -2.94 | -1.80 | -2.96 | -2.01 | CONSTANS-like |
| Tai6.10341 | 3.83 | 5.36 | 8.62 | NA | 4.99 | 5.48 | CONSTANS-like |
| Tai6.34586 | 0.22 | 1.61 | 2.82 | 2.47 | 3.25 | 3.76 | CONSTANS-like |
| Tai6.2022 | -0.79 | -1.25 | 0.94 | -1.62 | -1.31 | -1.87 | CONSTANS-like |
| Tai6.48287 | 3.12 | 4.91 | 7.31 | -3.10 | 1.16 | 2.36 | CONSTANS-like |
| Tai6.38540 | NA | 4.98 | 5.37 | 5.47 | 6.90 | 3.96 | CONSTANS-like |
| Tai6.24991 | 1.58 | 2.04 | 4.11 | 2.32 | 3.75 | 5.07 | CONSTANS-like |
| Tai6.17524 | -0.30 | 0.39 | 1.31 | 0.79 | 2.26 | 2.49 | CONSTANS-like |
| Tai6.40922 | 0.40 | 0.47 | 1.58 | 1.30 | 1.86 | 2.54 | CONSTANS-like |
| Tai6.37000 | 0.52 | 0.60 | 1.46 | 1.04 | 1.91 | 2.20 | CONSTANS-like |
| Tai6.2880 | -0.02 | 0.10 | 1.38 | 1.59 | 2.62 | 3.05 | CONSTANS-like |
| Tai6.6711 | 2.00 | 2.65 | 2.76 | 1.48 | 4.94 | 5.76 | CONSTANS-like |
| Tai6.45563 | 3.85 | 5.63 | 8.17 | -3.91 | 3.20 | 3.98 | CONSTANS-like |
| Tai6.51911 | 0.96 | 0.28 | 3.93 | 2.52 | -1.25 | -3.33 | CONSTANS-like |
| Tai6.29839 | 3.25 | 2.98 | 4.50 | 1.64 | 2.63 | 2.56 | phytochrome kinase |
| Tai6.10677 | 3.27 | 3.06 | 4.80 | 2.34 | 3.13 | 2.53 | phytochrome kinase |
| Tai6.1452 | 1.58 | 1.72 | 2.35 | 1.96 | 1.89 | 2.34 | phytochromes |
| Tai6.16858 | 1.75 | 1.62 | 1.89 | 2.41 | 3.01 | 3.48 | phytochromes |
| Tai6.31795 | 1.72 | 1.46 | 3.48 | 1.99 | 1.77 | 1.52 | phytochromes |
| Tai6.49806 | 1.98 | 2.60 | 3.55 | 1.92 | 1.91 | 2.34 | phytochromes |
| Tai6.50519 | 0.36 | 1.01 | 1.65 | 1.24 | 1.31 | 1.16 | phytochromes |
| Tai6.33324 | -0.05 | 0.36 | 1.04 | 0.49 | 0.79 | 1.03 | phytochromes |
| Tai6.14617 | 2.57 | 1.22 | 0.65 | 0.80 | 1.28 | 1.61 | COP-interactive proteins genes |
| Tai6.17879 | 1.91 | 1.49 | 1.26 | 0.91 | 1.26 | 1.82 | COP-interactive proteins genes |
| Tai6.28382 | 3.31 | 3.61 | 4.00 | 1.22 | 1.34 | 4.28 | COP-interactive proteins genes |
| Tai6.38298 | -1.35 | -1.69 | -3.38 | -0.41 | -1.89 | -3.95 | COP-interactive proteins genes |
| Tai6.43220 | 0.75 | 1.84 | 1.07 | 1.20 | 0.76 | 1.00 | COP-interactive proteins genes |
| Tai6.2391 | 0.47 | 0.81 | 2.46 | 0.75 | 0.91 | 1.03 | COP-interactive proteins genes |
| Tai6.43088 | -4.34 | -4.51 | -4.90 | -0.97 | -2.31 | -6.13 | BEL related genes |
| Tai6.46822 | 3.04 | 3.50 | 4.27 | 2.14 | 3.03 | 4.19 | BEL related genes |
| Tai6.44490 | 2.14 | 2.06 | 3.10 | 2.11 | 2.52 | 3.79 | BEL related genes |
| Tai6.31611 | 2.97 | 3.38 | 4.44 | 2.11 | 2.75 | 3.91 | BEL related genes |
| Tai6.36202 | 2.54 | 2.98 | 3.45 | 1.97 | 2.53 | 3.56 | BEL related genes |
| Tai6.53028 | 1.31 | 1.47 | 1.78 | 1.41 | 0.95 | 1.09 | BEL related genes |
| Tai6.22648 | 1.23 | 2.71 | 3.57 | 1.93 | 2.35 | 3.18 | BEL related genes |
| Tai6.42353 | 1.12 | 2.29 | 3.42 | 1.83 | 2.31 | 3.15 | BEL related genes |
| Tai6.675 | 1.19 | 3.04 | 3.66 | 2.04 | 2.89 | 3.55 | BEL related genes |
| Tai6.22647 | 0.73 | 2.72 | 3.30 | 1.79 | 2.62 | 3.50 | BEL related genes |
| Tai6.32256 | 0.00 | -2.86 | -2.39 | -0.02 | -1.63 | -1.66 | BEL related genes |
| Tai6.35938 | 0.17 | -2.96 | -2.20 | 0.38 | -1.72 | -2.06 | BEL related genes |
| Tai6.54407 | 1.47 | 2.81 | 3.64 | 2.20 | 2.18 | 3.28 | BEL related genes |
| Tai6.14585 | 0.30 | -2.42 | -0.53 | 0.09 | -1.39 | -2.04 | BEL related genes |
| Tai6.47011 | 1.11 | 1.69 | 2.59 | 1.43 | 1.29 | 1.79 | BEL related genes |
| Tai6.673 | 0.21 | 1.62 | 1.80 | 2.34 | 2.34 | 2.79 | BEL related genes |
| Tai6.2549 | 1.80 | 1.47 | 2.90 | 5.89 | 3.69 | NA | BEL related genes |
| Tai6.19544 | 0.02 | 0.13 | 1.32 | 1.35 | 0.49 | 0.07 | BEL related genes |
| Tai6.50640 | 1.24 | 0.77 | 1.55 | 1.06 | 1.49 | 2.07 | BEL related genes |

**Table S9** Among all DEGs shared by GJS-8 and XGH, the genes related to cell wall and cell cycle .

| Genes | RGJ8_1vsRGJ8_0_log2FoldChange | RGJ8_2vsRGJ8_0_log2FoldChange | RGJ8_3vsRGJ8_0_log2FoldChange | RXGH_1vsRXGH_0_log2FoldChange | RXGH_2vsRXGH_0_log2FoldChange | RXGH_3vsRXGH_0_log2FoldChange | Description |
| --- | --- | --- | --- | --- | --- | --- | --- |
| Tai6.40235 | 3.97 | 2.07 | 3.24 | 0.36 | 3.13 | 4.95 | XTH |
| Tai6.23772 | 4.39 | 2.82 | -0.84 | -2.28 | -0.13 | 1.47 | XTH |
| Tai6.27392 | 3.94 | 2.63 | -0.33 | -3.21 | 0.30 | 1.99 | XTH |
| Tai6.7480 | 4.10 | 1.82 | 2.61 | 1.45 | 4.32 | 5.82 | XTH |
| Tai6.10257 | 1.73 | 0.45 | 0.16 | 0.00 | -0.62 | -1.54 | XTH |
| Tai6.30799 | 1.76 | -0.08 | 0.04 | 1.11 | 1.09 | 1.52 | XTH |
| Tai6.41314 | 1.68 | -0.89 | -4.24 | -0.40 | -2.79 | -2.29 | XTH |
| Tai6.33778 | 2.07 | 0.01 | -2.54 | -1.30 | -2.23 | -1.43 | XTH |
| Tai6.38747 | 3.48 | 2.82 | 0.62 | NA | 5.49 | 6.10 | XTH |
| Tai6.12156 | 3.22 | 2.10 | 2.64 | 2.30 | 2.48 | 2.80 | XTH |
| Tai6.33773 | 1.64 | -0.69 | -3.10 | -1.03 | -2.35 | -2.12 | XTH |
| Tai6.7477 | 1.41 | -1.44 | -3.72 | -0.21 | -2.87 | -1.03 | XTH |
| Tai6.41313 | 1.43 | -1.71 | -4.13 | -0.75 | -3.50 | -1.49 | XTH |
| Tai6.19976 | 1.25 | -1.68 | -4.01 | -0.86 | -3.56 | -1.61 | XTH |
| Tai6.39066 | 1.20 | -0.82 | -2.99 | -1.07 | -3.52 | -3.12 | XTH |
| Tai6.50165 | -1.32 | -1.87 | -2.20 | -2.18 | -1.18 | -1.38 | XTH |
| Tai6.50164 | -1.25 | -1.98 | -2.95 | -1.95 | -1.50 | -1.28 | XTH |
| Tai6.19977 | -0.08 | -3.00 | -4.45 | -1.13 | -4.69 | -3.35 | XTH |
| Tai6.41316 | -0.09 | -2.67 | -4.47 | -1.00 | -3.98 | -3.56 | XTH |
| Tai6.37601 | 0.23 | 1.78 | 0.34 | -1.33 | 1.57 | 2.35 | XTH |
| Tai6.51527 | -1.03 | -2.35 | -3.24 | -1.39 | -1.23 | -1.25 | XTH |
| Tai6.29120 | 0.10 | -2.00 | -4.64 | -1.54 | -2.70 | -2.12 | XTH |
| Tai6.1268 | -1.46 | -2.07 | -3.16 | -1.53 | -1.94 | -1.64 | XTH |
| Tai6.39068 | 0.52 | -1.53 | -3.94 | -1.71 | -2.61 | -1.57 | XTH |
| Tai6.34778 | 0.48 | -1.33 | -2.79 | -1.48 | -1.35 | -1.71 | XTH |
| Tai6.34098 | 0.03 | -0.54 | 1.18 | 1.81 | 0.32 | -0.90 | XTH |
| Tai6.7478 | 2.83 | 1.29 | -1.94 | 1.38 | -0.41 | 0.23 | XTH |
| Tai6.49879 | 1.98 | 0.40 | -2.09 | 1.59 | 1.69 | 2.80 | XTH |
| Tai6.23751 | 0.44 | -1.14 | -2.65 | -1.45 | -1.67 | -2.28 | XTH |
| Tai6.7060 | -10.37 | -7.46 | -10.68 | -7.26 | -5.60 | -7.28 | expansin |
| Tai6.45007 | 2.63 | 0.86 | -0.45 | -1.29 | 1.38 | 2.17 | expansin |
| Tai6.22652 | 2.66 | 1.04 | 0.09 | -1.94 | 0.85 | 2.01 | expansin |
| Tai6.8814 | 2.51 | 2.15 | 0.28 | 0.20 | 1.41 | 2.05 | expansin |
| Tai6.39878 | -8.84 | -8.95 | -9.14 | -4.41 | -6.28 | -6.22 | expansin |
| Tai6.24528 | 3.39 | 2.55 | 1.85 | NA | 4.18 | 4.53 | expansin |
| Tai6.30690 | -7.73 | -7.84 | -8.03 | -5.84 | -5.93 | -5.86 | expansin |
| Tai6.37400 | 2.64 | 0.18 | -3.53 | 0.30 | 2.78 | 3.74 | expansin |
| Tai6.15410 | 3.49 | 4.13 | 0.53 | NA | 5.85 | 7.51 | expansin |
| Tai6.39708 | 2.10 | 2.03 | 1.02 | 1.62 | 1.82 | 2.21 | expansin |
| Tai6.2725 | 5.11 | 5.94 | 2.43 | 2.80 | 4.12 | 3.23 | expansin |
| Tai6.29231 | -1.50 | -2.42 | -2.54 | -3.77 | -3.71 | -7.95 | expansin |
| Tai6.10018 | 2.29 | 1.59 | -3.85 | 3.60 | 5.34 | 4.64 | expansin |
| Tai6.35918 | 4.96 | 6.07 | 4.90 | 1.11 | 2.42 | 1.92 | expansin |
| Tai6.48229 | -0.54 | -0.99 | -1.72 | -1.15 | -1.92 | -1.87 | expansin |
| Tai6.6176 | -4.72 | -4.83 | -5.03 | -6.70 | -6.78 | -6.72 | expansin |
| Tai6.27356 | -6.32 | -7.70 | -8.01 | -6.95 | -10.10 | -10.03 | expansin |
| Tai6.25366 | -3.80 | -2.58 | -3.48 | -1.74 | -7.73 | -3.31 | expansin |
| Tai6.3314 | 3.13 | 3.69 | 1.26 | -0.92 | 2.47 | 2.96 | expansin |
| Tai6.48325 | 3.86 | 4.03 | 0.80 | NA | 5.67 | 7.58 | expansin |
| Tai6.42950 | 1.66 | 1.71 | 0.29 | 1.00 | 1.63 | 2.02 | expansin |
| Tai6.40940 | -5.12 | -8.74 | -8.93 | -3.57 | -7.08 | -7.01 | expansin |
| Tai6.37317 | -1.18 | -2.50 | -3.75 | -1.60 | -1.48 | -2.73 | extensin |
| Tai6.10380 | -3.01 | -0.15 | -1.34 | -4.30 | -6.18 | -6.12 | extensin |
| Tai6.16217 | 0.99 | -2.31 | -6.36 | -5.67 | -0.97 | 0.06 | extensin |
| Tai6.41443 | 2.70 | 2.39 | 1.44 | 1.06 | 2.49 | 3.36 | FtsZ |
| Tai6.24331 | 2.47 | 2.13 | 2.21 | 1.84 | 3.01 | 3.71 | FtsZ |
| Tai6.8819 | 1.40 | 1.50 | 1.01 | 1.54 | 2.09 | 2.23 | FtsZ |
| Tai6.17057 | 2.53 | 2.50 | 2.75 | 2.43 | 3.86 | 4.37 | FtsZ |
| Tai6.48637 | 1.92 | 1.82 | 2.03 | 1.74 | 2.98 | 3.40 | FtsZ |
| Tai6.53863 | 1.83 | 2.66 | 1.66 | 2.10 | 2.66 | 2.73 | FtsZ |
| Tai6.41442 | 1.64 | 1.43 | 0.76 | 1.41 | 2.02 | 2.47 | FtsZ |
| Tai6.15786 | 2.07 | 1.98 | 2.43 | 1.83 | 2.90 | 3.02 | FtsZ |
| Tai6.14677 | 0.89 | 1.85 | 3.01 | 2.09 | 2.76 | 3.06 | CDC5 |
| Tai6.27497 | 0.77 | 2.03 | 3.11 | 0.18 | 1.29 | 1.75 | CDC5 |
| Tai6.27495 | 0.70 | 1.17 | 1.92 | 0.43 | 1.18 | 1.64 | CDC5 |
| Tai6.14678 | 0.72 | 0.76 | 1.72 | 0.45 | 1.12 | 1.69 | CDC5 |
| Tai6.27466 | 0.39 | 0.81 | 1.30 | 0.01 | -0.03 | 1.13 | CDC5 |
| Tai6.13582 | 1.23 | 2.54 | 2.97 | 0.51 | 0.74 | 1.67 | CDC5 |
| Tai6.33532 | -4.53 | -4.74 | -8.54 | -5.45 | -5.27 | -5.48 | CDC |
| Tai6.40703 | -3.93 | -4.40 | -6.56 | -3.67 | -6.15 | -6.76 | CDC |
| Tai6.46711 | -4.63 | -5.56 | -6.59 | -3.57 | -7.26 | -5.99 | CDC |
| Tai6.40700 | -3.88 | -5.37 | -6.64 | -4.22 | -6.84 | -7.63 | CDC |
| Tai6.27262 | 1.12 | 1.62 | 2.11 | 2.67 | 3.14 | 2.73 | CDC |
| Tai6.24627 | -0.97 | -1.49 | -1.43 | -0.65 | -1.23 | -1.05 | CDC |
| Tai6.8609 | 1.43 | 1.25 | 0.71 | 1.25 | 1.48 | 1.70 | CDC |
| Tai6.2172 | 0.59 | 0.58 | 1.73 | 1.49 | 3.45 | 2.77 | CDC |
| Tai6.37188 | 0.88 | 0.92 | 1.47 | 0.87 | 2.39 | 1.82 | CDC |
| Tai6.45276 | -0.54 | -0.47 | -1.51 | -0.39 | -0.97 | -1.33 | CDK |
| Tai6.20766 | -0.39 | 0.06 | 1.12 | 1.03 | 1.37 | 1.30 | CDK |
| Tai6.34932 | 2.82 | 2.75 | 1.18 | 0.76 | 1.56 | 2.38 | CDK |
| Tai6.51091 | -4.15 | -2.24 | -1.44 | -1.50 | -2.80 | -6.65 | CDK |
| Tai6.18929 | 1.10 | 0.66 | -1.35 | -2.46 | 0.00 | 0.25 | CDK |
| Tai6.54357 | -2.09 | -2.23 | -5.51 | 0.93 | -1.05 | -2.35 | CDK |
| Tai6.20390 | -0.84 | -1.32 | -1.91 | -0.29 | -1.57 | -1.70 | CDK |
| Tai6.47554 | -2.40 | -1.05 | -0.86 | -1.09 | -0.66 | -1.02 | CDKI |
| Tai6.54898 | 3.59 | 3.42 | 2.67 | 0.99 | 2.41 | 3.04 | CDKI |
| Tai6.14247 | -0.98 | -5.07 | -5.65 | -1.60 | -4.66 | -4.18 | CDKI |
| Tai6.14248 | -0.60 | -4.38 | -4.97 | -1.30 | -4.42 | -3.46 | CDKI |
| Tai6.17043 | 0.86 | 0.76 | 2.33 | 0.42 | 2.12 | 2.73 | CDKI |
| Tai6.20391 | -0.74 | -0.55 | -1.31 | -0.33 | -0.83 | -1.59 | CDKI |
| Tai6.35770 | -2.91 | -2.28 | -5.38 | -6.62 | -5.50 | -5.56 | CDKI |
| Tai6.48434 | 2.67 | 3.18 | 3.34 | 3.35 | 4.82 | 5.01 | CDKI |
| Tai6.33040 | 2.72 | 2.87 | 4.15 | 2.30 | 4.59 | 3.83 | CDKI |
| Tai6.14275 | 2.60 | 1.84 | 2.92 | 3.48 | 5.97 | 5.69 | CDKI |
| Tai6.22797 | 1.74 | 1.23 | 2.12 | 1.28 | 1.38 | 0.42 | CDKI |

**Table S10** Among all DEGs shared by GJS-8 and XGH, the genes related to starch and sucrose metabolism.

| Gene-ID | RGJ8_1vsRGJ8_0_log2FoldChange | RGJ8_2vsRGJ8_0_log2FoldChange | RGJ8_3vsRGJ8_0_log2FoldChange | RXGH_1vsRXGH_0_log2FoldChange | RXGH_2vsRXGH_0_log2FoldChange | RXGH_3vsRXGH_0_log2FoldChange | Description |
| --- | --- | --- | --- | --- | --- | --- | --- |
| Tai6.13146 | 1.81 | -0.04 | -0.12 | -1.11 | -0.06 | 0.47 | SuSy |
| Tai6.43693 | 1.94 | 2.40 | 1.92 | 0.97 | 1.83 | 2.60 | SuSy |
| Tai6.43694 | 3.26 | -2.08 | -1.06 | -1.07 | -1.10 | -1.28 | SuSy |
| Tai6.13148 | 1.95 | 0.85 | 0.49 | -1.23 | 0.56 | 1.33 | SuSy |
| Tai6.12239 | 1.32 | 2.42 | 3.82 | 1.65 | 2.53 | 3.04 | SuSy |
| Tai6.17029 | 1.80 | 1.04 | 1.25 | 0.70 | 2.96 | 2.82 | SuSy |
| Tai6.12238 | 1.04 | 1.57 | 2.90 | 1.33 | 1.85 | 2.49 | SuSy |
| Tai6.54702 | 2.16 | 1.43 | 1.17 | -2.13 | 1.98 | 2.13 | SuSy |
| Tai6.38791 | 1.43 | 2.46 | 3.91 | 1.57 | 2.22 | 2.86 | SuSy |
| Tai6.7701 | 1.67 | 1.34 | 1.83 | -1.60 | 2.66 | 2.61 | SuSy |
| Tai6.29613 | 1.22 | 0.32 | 0.84 | -0.04 | 2.46 | 2.54 | SuSy |
| Tai6.38107 | 1.30 | 2.02 | 3.36 | 1.25 | 1.89 | 2.38 | SuSy |
| Tai6.38789 | 1.25 | 1.68 | 2.99 | 1.17 | 1.67 | 2.29 | SuSy |
| Tai6.34487 | 1.00 | 2.36 | 1.91 | 0.72 | 1.45 | 2.23 | SPS |
| Tai6.24187 | 2.56 | 3.56 | 3.19 | 1.27 | 2.35 | 2.71 | SPS |
| Tai6.44980 | 2.70 | 3.08 | 2.45 | 1.30 | 2.42 | 3.95 | SS |
| Tai6.14480 | 3.59 | 4.97 | 4.44 | 2.56 | 4.97 | 5.35 | SS |
| Tai6.11941 | 2.88 | 3.46 | 2.86 | 0.96 | 1.95 | 3.39 | SS |
| Tai6.11942 | 4.25 | 5.86 | 4.79 | 1.78 | 2.87 | 4.25 | SS |
| Tai6.14481 | 1.72 | 2.67 | 1.63 | 1.10 | 3.11 | 3.86 | SS |
| Tai6.13470 | 1.40 | 1.69 | 2.39 | 2.17 | 2.57 | 2.34 | SS |
| Tai6.2382 | 4.24 | 4.38 | 5.17 | 4.64 | 5.06 | 5.81 | SS |
| Tai6.13468 | 1.07 | 0.95 | 1.54 | 1.41 | 1.59 | 1.74 | SS |
| Tai6.51436 | 4.90 | 5.28 | 5.72 | 1.90 | 3.46 | 3.96 | SS |
| Tai6.53320 | 2.82 | 3.42 | 3.64 | 3.41 | 5.21 | 6.46 | SS |
| Tai6.37221 | 6.46 | 2.39 | 3.51 | -0.19 | 3.84 | 3.91 | INV |
| Tai6.26803 | 2.80 | 2.15 | 1.49 | 0.07 | 1.50 | 2.10 | INV |
| Tai6.35661 | -2.04 | -1.91 | -4.24 | -2.40 | -4.99 | -5.13 | INV |
| Tai6.35660 | -2.42 | -3.03 | -6.53 | -3.19 | -5.68 | -6.43 | INV |
| Tai6.47650 | 2.01 | 1.67 | 2.94 | 0.52 | 2.19 | 2.64 | INV |
| Tai6.6679 | 5.25 | 6.49 | 6.29 | 1.93 | 4.41 | 6.31 | GBSS |
| Tai6.6680 | 4.97 | 6.72 | 6.46 | 2.09 | 5.09 | 6.87 | GBSS |
| Tai6.18732 | 4.91 | 6.14 | 6.17 | 1.63 | 4.33 | 6.42 | GBSS |
| Tai6.3651 | 5.09 | 6.97 | 7.00 | 2.09 | 4.82 | 6.64 | GBSS |
| Tai6.26337 | 3.23 | 4.21 | 4.97 | 2.62 | 3.72 | 4.42 | GBSS |
| Tai6.25704 | 3.24 | 4.25 | 4.64 | 2.41 | 3.71 | 4.57 | GBSS |
| Tai6.22333 | 6.34 | 5.87 | 4.98 | NA | 7.70 | 9.34 | GBSS |
| Tai6.50687 | -2.21 | -1.91 | -2.66 | -1.74 | -1.26 | -2.04 | GBSS |
| Tai6.22331 | 5.83 | 4.87 | 6.38 | 4.79 | 5.75 | 6.83 | GBSS |
| Tai6.23496 | -0.49 | -3.07 | -4.17 | -1.50 | -4.71 | -2.53 | GBSS |
| Tai6.49382 | 3.45 | 4.56 | 3.74 | 1.47 | 3.20 | 3.84 | SSS |
| Tai6.11939 | 3.55 | 4.06 | 2.64 | 1.19 | 2.38 | 3.71 | SSS |
| Tai6.22200 | 2.27 | 3.34 | 2.30 | 1.32 | 2.59 | 3.10 | SSS |
| Tai6.44979 | 2.37 | 3.03 | 2.32 | 2.85 | 3.34 | 5.57 | SSS |
| Tai6.5328 | 2.26 | 2.63 | 2.24 | 1.97 | 3.23 | 4.00 | SBE |
| Tai6.5330 | 2.56 | 3.36 | 3.14 | 2.18 | 3.53 | 4.11 | SBE |
| Tai6.43557 | 2.48 | 3.73 | 3.17 | 2.39 | 4.04 | 4.59 | SBE |
| Tai6.53925 | 2.33 | 3.52 | 2.81 | 2.21 | 3.42 | 4.00 | SBE |
| Tai6.49478 | 3.36 | 4.86 | 4.29 | 2.14 | 3.66 | 4.57 | SBE |
| Tai6.32912 | 4.30 | 5.72 | 5.16 | 2.12 | 5.69 | 6.73 | SBE |
| Tai6.32914 | 6.21 | 8.09 | 7.19 | 3.93 | 7.93 | 9.04 | SBE |
| Tai6.32175 | 1.20 | 1.03 | 0.51 | 0.43 | -0.05 | 1.19 | SBE |
| Tai6.7676 | 2.15 | 0.62 | 0.75 | -0.77 | -1.39 | -1.15 | SBE |
| Tai6.7675 | 1.07 | 1.54 | 4.01 | 2.35 | 2.38 | 2.33 | SBE |
| Tai6.35527 | -0.43 | -0.83 | -1.86 | -0.98 | -1.41 | -1.35 | SBE |
| Tai6.6953 | 15.29 | 16.31 | 13.80 | -0.91 | 7.51 | 8.57 | Beta-amylase |
| Tai6.470 | 4.03 | 5.32 | 4.03 | -1.87 | 9.52 | 9.78 | Beta-amylase |
| Tai6.52712 | 1.28 | 2.28 | 1.79 | 1.35 | 1.32 | 0.91 | Beta-amylase |
| Tai6.24843 | 5.50 | 8.63 | 7.93 | NA | 10.40 | 10.72 | Beta-amylase |
| Tai6.4222 | 0.94 | 1.30 | 0.30 | 0.70 | 0.38 | 1.06 | Beta-amylase |
| Tai6.53184 | -3.17 | -1.90 | -3.33 | -4.96 | -3.34 | -3.02 | alpha-amylase |
| Tai6.3947 | -2.45 | -1.33 | -2.24 | -1.76 | -1.76 | -1.28 | alpha-amylase |
| Tai6.47656 | -2.41 | -3.51 | -4.16 | -2.59 | -1.73 | -2.18 | alpha-amylase |
| Tai6.3946 | -1.51 | -0.03 | -0.56 | -1.84 | -2.53 | -2.03 | alpha-amylase |
| Tai6.4702 | 0.39 | 0.80 | 1.03 | 0.89 | 0.83 | 1.10 | alpha-amylase |
| Tai6.40042 | 6.00 | 7.50 | 7.11 | 3.22 | 5.28 | 6.93 | isoamylase |
| Tai6.43440 | 6.23 | 7.60 | 7.40 | 3.29 | 5.71 | 7.09 | isoamylase |
| Tai6.7562 | 1.42 | 1.28 | 0.94 | 1.95 | 1.96 | 2.29 | isoamylase |
| Tai6.3161 | 1.84 | 1.70 | 1.43 | 1.07 | 2.09 | 2.64 | isoamylase |
| Tai6.3160 | 1.89 | 2.07 | 2.10 | 2.05 | 2.34 | 2.34 | isoamylase |

**Table S11** TF from all DEGs shared by GJS-8 and XGH.

| geneID | RGJ8_1vsRGJ8_0_log2FoldChange | RGJ8_2vsRGJ8_0_log2FoldChange | RGJ8_3vsRGJ8_0_log2FoldChange | RXGH_1vsRXGH_0_log2FoldChange | RXGH_2vsRXGH_0_log2FoldChange | RXGH_3vsRXGH_0_log2FoldChange | Family |
| --- | --- | --- | --- | --- | --- | --- | --- |
| Tai6.39723 | 8.99 | 7.46 | 4.41 | 2.56 | 7.60 | 8.03 | bHLH |
| Tai6.25160 | 6.18 | 5.78 | 6.10 | 3.31 | 5.04 | 6.90 | bHLH |
| Tai6.6972 | 5.46 | 5.22 | 5.25 | 1.63 | 3.76 | 5.86 | bHLH |
| Tai6.48482 | 5.02 | 4.65 | 4.63 | 2.49 | 4.61 | 6.47 | bHLH |
| Tai6.2655 | 4.28 | 3.87 | 4.27 | 3.73 | 7.05 | 7.84 | HB |
| Tai6.41030 | 4.26 | 2.42 | 1.78 | 3.44 | 4.24 | 4.16 | C2C2 |
| Tai6.36911 | 4.14 | 4.10 | 3.89 | 2.77 | 1.51 | 2.69 | MYB |
| Tai6.10338 | 4.14 | 4.77 | 5.42 | 4.13 | 4.84 | 4.02 | zf-HD |
| Tai6.54979 | 4.10 | 4.32 | 5.18 | 3.79 | 4.45 | 5.16 | MYB |
| Tai6.40804 | 4.09 | 5.42 | 6.10 | 2.10 | 1.05 | 1.68 | NAC |
| Tai6.37032 | 3.87 | 4.70 | 5.84 | 3.59 | 4.83 | 4.75 | TRAF |
| Tai6.23445 | 3.66 | 2.10 | 2.04 | 1.34 | 2.45 | 3.33 | C2H2 |
| Tai6.17474 | 3.62 | 4.22 | 3.54 | 1.79 | 2.33 | 2.81 | Trihelix |
| Tai6.14401 | 3.50 | 3.32 | 4.31 | 1.33 | 3.09 | 2.73 | MYB |
| Tai6.21473 | 3.42 | 4.20 | 2.94 | 1.79 | 3.23 | 3.71 | PLATZ |
| Tai6.23289 | 3.37 | 3.33 | 4.99 | 1.84 | 2.44 | 2.43 | GARP |
| Tai6.10677 | 3.27 | 3.06 | 4.80 | 2.34 | 3.13 | 2.53 | bHLH |
| Tai6.7362 | 3.16 | 3.14 | 3.81 | 3.38 | 3.60 | 4.99 | GARP |
| Tai6.35293 | 3.11 | 3.34 | 4.84 | 2.24 | 3.62 | 3.56 | GARP |
| Tai6.35293 | 3.11 | 3.34 | 4.84 | 2.24 | 3.62 | 3.56 | GARP |
| Tai6.46822 | 3.04 | 3.50 | 4.27 | 2.14 | 3.03 | 4.19 | HB |
| Tai6.54505 | 3.01 | 3.55 | 4.45 | 2.66 | 4.37 | 4.40 | MYB |
| Tai6.31611 | 2.97 | 3.38 | 4.44 | 2.11 | 2.75 | 3.91 | HB |
| Tai6.37290 | 2.96 | 3.14 | 3.61 | 1.39 | 2.21 | 1.93 | MYB |
| Tai6.44974 | 2.92 | 1.87 | 5.50 | 2.15 | 1.62 | 2.92 | C2C2 |
| Tai6.19763 | 2.89 | 3.66 | 4.99 | 2.64 | 3.46 | 3.10 | HB |
| Tai6.22026 | 2.85 | 3.02 | 2.88 | 1.93 | 3.27 | 3.76 | HB |
| Tai6.50152 | 2.83 | 2.05 | 2.85 | 1.45 | 1.16 | 3.29 | SBP |
| Tai6.34818 | 2.75 | 3.25 | 3.97 | 1.97 | 4.03 | 4.69 | OFP |
| Tai6.49035 | 2.75 | 3.84 | 4.75 | 1.49 | 5.46 | 7.72 | MYB |
| Tai6.18777 | 2.73 | 2.76 | 2.80 | 1.45 | 2.26 | 2.70 | C3H |
| Tai6.11112 | 2.69 | 2.24 | 4.30 | 2.33 | 3.58 | 2.94 | zf-HD |
| Tai6.35550 | 2.69 | 2.64 | 3.45 | 2.21 | 2.43 | 3.34 | HB |
| Tai6.33025 | 2.68 | 3.09 | 4.47 | 1.46 | 1.84 | 2.48 | Trihelix |
| Tai6.8000 | 2.62 | 3.06 | 3.74 | 1.13 | 2.15 | 1.39 | AP2/ERF |
| Tai6.8322 | 2.60 | 1.82 | 2.28 | 1.37 | 2.72 | 4.10 | Jumonji |
| Tai6.29439 | 2.59 | 2.91 | 3.03 | 1.64 | 3.15 | 3.38 | C2H2 |
| Tai6.36202 | 2.54 | 2.98 | 3.45 | 1.97 | 2.53 | 3.56 | HB |
| Tai6.21109 | 2.51 | 2.44 | 3.35 | 1.40 | 1.93 | 2.88 | C2H2 |
| Tai6.6500 | 2.51 | 1.86 | 1.20 | 2.22 | 2.72 | 3.21 | Whirly |
| Tai6.18811 | 2.51 | 1.85 | 1.99 | 1.74 | 1.88 | 1.72 | GARP |
| Tai6.17660 | 2.48 | 2.55 | 2.72 | 2.04 | 3.45 | 3.19 | bHLH |
| Tai6.42462 | 2.47 | 2.92 | 2.28 | 1.22 | 2.72 | 2.92 | bZIP |
| Tai6.25588 | 2.46 | 2.10 | 1.49 | 1.21 | 2.73 | 2.25 | Whirly |
| Tai6.9409 | 2.42 | 1.87 | 1.91 | 1.25 | 2.30 | 2.24 | Tify |
| Tai6.21282 | 2.41 | 1.93 | 2.47 | 1.59 | 1.84 | 2.19 | HB |
| Tai6.28955 | 2.40 | 2.86 | 2.77 | 2.04 | 6.18 | 8.36 | MYB |
| Tai6.13876 | 2.39 | 2.26 | 2.51 | 2.15 | 3.02 | 3.12 | C2H2 |
| Tai6.14743 | 2.38 | 2.11 | 3.10 | 2.48 | 2.83 | 3.58 | GARP |
| Tai6.31049 | 2.35 | 1.49 | 1.19 | 1.15 | 1.02 | 1.64 | Jumonji |
| Tai6.33758 | 2.33 | 2.21 | 2.99 | 1.69 | 1.85 | 2.99 | HB |
| Tai6.2430 | 2.31 | 2.53 | 3.55 | 1.40 | 1.85 | 1.99 | WRKY |
| Tai6.23759 | 2.29 | 2.12 | 2.92 | 1.97 | 2.09 | 2.95 | HB |
| Tai6.30863 | 2.24 | 3.06 | 2.73 | 1.89 | 1.70 | 2.91 | bHLH |
| Tai6.48276 | 2.21 | 1.48 | 1.41 | 2.60 | 2.41 | 2.97 | AUX/IAA |
| Tai6.37849 | 2.20 | 3.26 | 2.96 | 1.59 | 1.36 | 1.92 | HB |
| Tai6.28506 | 2.18 | 2.45 | 3.16 | 1.58 | 1.98 | 2.46 | C2H2 |
| Tai6.43837 | 2.17 | 3.80 | 3.33 | 3.77 | 3.63 | 4.10 | HB |
| Tai6.24971 | 2.15 | 2.30 | 3.05 | 2.48 | 2.52 | 2.99 | AUX/IAA |
| Tai6.44490 | 2.14 | 2.06 | 3.10 | 2.11 | 2.52 | 3.79 | HB |
| Tai6.15913 | 2.13 | 2.33 | 2.26 | 5.79 | 4.41 | 4.89 | C2H2 |
| Tai6.11522 | 2.13 | 2.23 | 2.24 | 1.92 | 2.99 | 3.12 | C2H2 |
| Tai6.39357 | 2.12 | 1.36 | 2.60 | 2.48 | 1.84 | 2.68 | bHLH |
| Tai6.27126 | 2.11 | 1.65 | 2.44 | 2.43 | 2.63 | 2.52 | HB |
| Tai6.9043 | 2.11 | 1.92 | 1.83 | 1.69 | 2.11 | 2.56 | BBR-BPC |
| Tai6.42753 | 2.09 | 2.35 | 3.03 | 2.87 | 2.62 | 2.65 | RB |
| Tai6.27324 | 2.08 | 2.04 | 2.94 | 2.14 | 2.23 | 2.68 | AUX/IAA |
| Tai6.2374 | 2.08 | 1.03 | 4.53 | 1.10 | 1.11 | 2.47 | C2C2 |
| Tai6.19075 | 2.07 | 1.84 | 2.01 | 1.09 | 1.27 | 2.57 | B3-ARF |
| Tai6.28426 | 2.03 | 1.98 | 2.81 | 2.13 | 3.21 | 3.74 | C3H |
| Tai6.28426 | 2.03 | 1.98 | 2.81 | 2.13 | 3.21 | 3.74 | C3H |
| Tai6.14003 | 1.97 | 1.84 | 1.94 | 1.68 | 1.95 | 1.61 | MADS |
| Tai6.50453 | 1.95 | 1.47 | 1.21 | 1.30 | 1.23 | 1.51 | SWI/SNF-BAF60b |
| Tai6.555 | 1.94 | 1.81 | 2.43 | 1.96 | 2.20 | 2.68 | AUX/IAA |
| Tai6.10686 | 1.93 | 1.91 | 3.73 | 2.68 | 2.72 | 2.83 | TRAF |
| Tai6.43850 | 1.88 | 3.14 | 4.95 | 2.08 | 2.38 | 3.76 | FAR1 |
| Tai6.34428 | 1.87 | 1.77 | 2.01 | 1.33 | 1.26 | 2.34 | B3-ARF |
| Tai6.36696 | 1.83 | 3.46 | 3.19 | 2.04 | 1.61 | 2.33 | HB |
| Tai6.7999 | 1.82 | 2.46 | 2.40 | 1.52 | 2.05 | 2.37 | AP2/ERF |
| Tai6.12513 | 1.81 | 3.08 | 4.04 | 1.53 | 2.16 | 1.01 | HB |
| Tai6.47905 | 1.80 | 1.46 | 2.09 | 1.50 | 2.02 | 1.49 | GARP |
| Tai6.5561 | 1.71 | 1.01 | 3.99 | 1.01 | 1.73 | 2.02 | C3H |
| Tai6.10285 | 1.71 | 2.08 | 4.41 | 3.01 | 4.83 | 4.31 | AP2/ERF |
| Tai6.3408 | 1.70 | 1.81 | 1.68 | 1.50 | 2.04 | 2.34 | Tify |
| Tai6.18460 | 1.69 | 1.65 | 2.20 | 2.38 | 2.08 | 2.12 | RB |
| Tai6.35786 | 1.67 | 1.74 | 3.66 | 2.41 | 1.88 | 2.46 | C2H2 |
| Tai6.8152 | 1.64 | 1.68 | 1.65 | 1.66 | 2.05 | 2.95 | B3-ARF |
| Tai6.2539 | 1.59 | 2.61 | 2.66 | 1.48 | 2.41 | 3.74 | NF-Y |
| Tai6.8327 | 1.58 | 1.26 | 1.45 | 2.00 | 2.30 | 2.85 | B3-ARF |
| Tai6.1909 | 1.50 | 1.08 | 1.27 | 1.70 | 2.58 | 2.79 | SNF2 |
| Tai6.4836 | 1.50 | 1.65 | 2.80 | 2.05 | 1.69 | 2.21 | GARP |
| Tai6.3468 | 1.49 | 1.88 | 3.04 | 2.67 | 2.67 | 2.32 | bZIP |
| Tai6.29213 | 1.48 | 1.54 | 2.51 | 1.16 | 2.83 | 2.10 | HB |
| Tai6.54407 | 1.47 | 2.81 | 3.64 | 2.20 | 2.18 | 3.28 | HB |
| Tai6.33205 | 1.47 | 1.81 | 2.11 | 1.66 | 1.47 | 2.48 | MYB |
| Tai6.43410 | 1.46 | 2.03 | 3.32 | 2.01 | 2.28 | 2.82 | GARP |
| Tai6.41523 | 1.44 | 1.16 | 2.08 | 1.42 | 1.86 | 2.73 | HB |
| Tai6.19743 | 1.41 | 1.59 | 2.99 | 2.03 | 2.21 | 2.35 | GARP |
| Tai6.29839 | 3.25 | 2.98 | 4.50 | 1.64 | 2.63 | 2.56 | bHLH |
| Tai6.8147 | 1.41 | 1.21 | 1.73 | 1.73 | 3.29 | 4.31 | AP2/ERF |
| Tai6.2888 | 1.40 | 1.80 | 2.93 | 2.21 | 1.38 | 1.47 | C2H2 |
| Tai6.12106 | 1.38 | 1.46 | 3.89 | 3.10 | 3.58 | 3.53 | HB |
| Tai6.43199 | 1.38 | 1.62 | 2.58 | 2.54 | 5.16 | 5.88 | OFP |
| Tai6.40312 | 1.36 | 1.13 | 1.48 | 2.28 | 2.52 | 2.29 | DDT |
| Tai6.52115 | 1.35 | 1.84 | 3.82 | 2.99 | 2.72 | 2.76 | GRAS |
| Tai6.28064 | 1.34 | 2.17 | 4.01 | 3.15 | 2.88 | 2.86 | GRAS |
| Tai6.23417 | 1.32 | 1.88 | 2.24 | 1.47 | 2.29 | 3.01 | HB |
| Tai6.29801 | 1.31 | 1.81 | 4.26 | 2.59 | 2.47 | 2.27 | TUB |
| Tai6.36805 | 1.30 | 1.49 | 3.01 | 1.41 | 1.33 | 2.01 | TCP |
| Tai6.18504 | 1.27 | 1.28 | 2.63 | 2.37 | 2.29 | 1.99 | HB |
| Tai6.15499 | 1.26 | 1.31 | 2.26 | 1.97 | 2.35 | 3.27 | C2H2 |
| Tai6.53840 | 1.23 | 1.25 | 1.70 | 1.83 | 1.38 | 2.25 | B3 |
| Tai6.22648 | 1.23 | 2.71 | 3.57 | 1.93 | 2.35 | 3.18 | HB |
| Tai6.16599 | 1.22 | 1.09 | 2.64 | 1.78 | 1.63 | 2.25 | GARP- |
| Tai6.29451 | 1.21 | 2.60 | 2.64 | 1.16 | 2.43 | 4.01 | NF-Y |
| Tai6.46022 | 1.20 | 1.73 | 3.37 | 3.23 | 2.57 | 2.84 | GRAS |
| Tai6.21659 | 1.16 | 1.49 | 2.31 | 1.28 | 1.47 | 2.29 | PHD |
| Tai6.8885 | 1.14 | 1.15 | 1.31 | 1.14 | 2.02 | 2.63 | C3H |
| Tai6.42353 | 1.12 | 2.29 | 3.42 | 1.83 | 2.31 | 3.15 | HB |
| Tai6.48864 | 1.09 | 1.14 | 3.37 | 2.50 | 2.50 | 2.31 | TUB |
| Tai6.35152 | 1.08 | 2.36 | 3.60 | 2.23 | 3.14 | 3.55 | GRAS |
| Tai6.34692 | 1.06 | 1.16 | 2.80 | 1.91 | 2.79 | 3.12 | C3H |
| Tai6.52240 | 1.05 | 1.34 | 2.72 | 1.71 | 1.97 | 3.09 | HB |
| Tai6.10174 | 1.05 | 1.52 | 2.82 | 2.12 | 2.28 | 3.15 | HB |
| Tai6.20284 | 1.04 | 1.28 | 2.36 | 1.83 | 1.68 | 2.56 | B3-ARF |
| Tai6.25300 | 1.02 | 1.79 | 3.15 | 1.90 | 2.26 | 2.56 | Trihelix |
| novel.209 | -7.28 | -3.59 | -6.22 | -3.23 | -2.73 | -4.57 | NAC |
| Tai6.23333 | -6.29 | -4.73 | -6.89 | -8.08 | -6.31 | -6.33 | Tify |
| Tai6.16385 | -6.20 | -10.76 | -10.95 | -7.25 | -1.44 | -6.60 | C2H2 |
| Tai6.23570 | -6.17 | -1.41 | -4.60 | -8.81 | -6.88 | -5.93 | AP2/ERF |
| Tai6.13450 | -6.09 | -3.60 | -6.39 | -2.80 | -2.89 | -1.04 | bHLH |
| Tai6.20990 | -5.84 | -1.53 | -5.29 | -6.64 | -5.84 | -5.18 | AP2/ERF |
| Tai6.23231 | -5.63 | -5.29 | -9.60 | -4.06 | -4.19 | -6.17 | WRKY |
| Tai6.23995 | -5.63 | -1.69 | -4.37 | -5.09 | -3.60 | -6.16 | AP2/ERF |
| Tai6.20525 | -5.42 | -4.74 | -6.39 | -4.96 | -3.90 | -5.56 | WRKY |
| Tai6.39862 | -5.31 | -3.12 | -5.10 | -8.06 | -5.65 | -4.73 | Tify |
| Tai6.2779 | -5.01 | -6.54 | -6.80 | -8.33 | -4.32 | -13.28 | WRKY |
| Tai6.2426 | -4.77 | -3.03 | -3.95 | -3.09 | -2.15 | -6.20 | WRKY |
| Tai6.16972 | -4.72 | -5.11 | -6.65 | -2.26 | -3.27 | -4.63 | bHLH |
| Tai6.29656 | -4.72 | -4.30 | -3.96 | -2.90 | -2.48 | -6.88 | WRKY |
| novel.184 | -4.53 | -3.94 | -5.51 | -3.67 | -4.49 | -5.48 | bHLH |
| Tai6.32866 | -4.47 | -4.21 | -6.85 | -4.50 | -3.38 | -6.32 | WRKY |
| Tai6.39645 | -4.37 | -1.81 | -3.51 | -4.66 | -3.35 | -4.03 | NAC |
| Tai6.25385 | -4.33 | -5.95 | -9.41 | -3.80 | -4.31 | -4.17 | NAC |
| Tai6.50246 | -4.27 | -4.37 | -4.58 | -3.33 | -3.42 | -3.35 | bHLH |
| Tai6.38771 | -4.25 | -2.45 | -3.85 | -6.60 | -4.92 | -3.38 | NAC |
| Tai6.1936 | -3.98 | -3.77 | -5.82 | -8.97 | -6.40 | -7.36 | bHLH |
| Tai6.10006 | -3.96 | -3.70 | -6.24 | -6.44 | -5.63 | -5.26 | MYB |
| Tai6.31214 | -3.95 | -3.92 | -4.83 | -4.10 | -3.88 | -7.69 | C2H2 |
| Tai6.39775 | -3.92 | -5.87 | -6.07 | -3.97 | -4.05 | -3.99 | MYB |
| Tai6.5904 | -3.92 | -1.65 | -3.38 | -6.90 | -4.26 | -3.39 | NAC |
| Tai6.39415 | -3.82 | -1.37 | -3.54 | -4.11 | -3.40 | -2.75 | B3 |
| Tai6.4739 | -3.79 | -5.71 | -5.78 | -4.03 | -5.56 | -5.44 | WRKY |
| Tai6.311 | -3.74 | -3.94 | -6.67 | -6.56 | -2.21 | -6.60 | WRKY |
| Tai6.11161 | -3.72 | -2.40 | -3.75 | -6.00 | -5.69 | -5.08 | Tify |
| Tai6.25702 | -3.69 | -4.68 | -3.95 | -2.61 | -5.22 | -6.22 | NAC |
| Tai6.25702 | -3.69 | -4.68 | -3.95 | -2.61 | -5.22 | -6.22 | NAC |
| Tai6.15517 | -3.64 | -2.00 | -3.93 | -1.03 | -4.81 | -4.75 | NAC |
| Tai6.20503 | -3.59 | -4.97 | -3.16 | -2.37 | -4.78 | -5.45 | NAC |
| Tai6.7893 | -3.54 | -2.68 | -4.41 | -4.20 | -3.49 | -7.57 | WRKY |
| Tai6.8831 | -3.53 | -2.06 | -4.02 | -5.53 | -5.88 | -7.05 | WRKY |
| Tai6.4533 | -3.51 | -3.34 | -5.63 | -4.98 | -3.43 | -5.35 | HSF |
| Tai6.28866 | -3.50 | -6.58 | -7.13 | -4.95 | -6.74 | -5.94 | WRKY |
| Tai6.12658 | -3.45 | -1.76 | -4.18 | -9.01 | -9.10 | -9.03 | bHLH |
| Tai6.48108 | -3.42 | -3.18 | -5.17 | -5.98 | -5.38 | -8.44 | WRKY |
| Tai6.49584 | -3.36 | -1.43 | -3.43 | -5.28 | -4.30 | -4.64 | NAC |
| Tai6.19508 | -3.32 | -6.43 | -6.74 | -4.61 | -6.34 | -5.94 | WRKY |
| Tai6.10828 | -3.28 | -2.76 | -4.91 | -5.36 | -4.68 | -8.24 | WRKY |
| Tai6.14505 | -3.25 | -3.27 | -5.16 | -5.04 | -3.93 | -5.12 | HSF |
| Tai6.27180 | -3.23 | -2.72 | -6.40 | -5.52 | -4.42 | -5.98 | WRKY |
| Tai6.7968 | -3.23 | -5.19 | -4.17 | -3.47 | -2.32 | -7.71 | MYB |
| Tai6.44393 | -3.22 | -5.66 | -5.21 | -3.14 | -5.01 | -4.95 | WRKY |
| Tai6.36357 | -3.13 | -4.72 | -2.11 | -2.49 | -2.67 | -3.71 | NAC |
| Tai6.9244 | -3.10 | -3.32 | -2.65 | -2.62 | -1.16 | -4.92 | WRKY |
| Tai6.50086 | -3.07 | -3.49 | -2.87 | -2.05 | -2.15 | -3.40 | NAC |
| Tai6.22762 | -3.06 | -2.21 | -1.82 | -2.73 | -2.39 | -3.10 | NAC |
| Tai6.2939 | -3.05 | -2.67 | -5.25 | -5.93 | -4.28 | -6.65 | WRKY |
| Tai6.5134 | -3.05 | -3.23 | -1.14 | -2.23 | -2.51 | -3.90 | HMG |
| Tai6.44706 | -3.01 | -1.57 | -3.25 | -3.60 | -2.78 | -2.61 | MYB |
| Tai6.36611 | -3.00 | -2.60 | -4.10 | -5.23 | -3.11 | -6.03 | WRKY |
| Tai6.40433 | -3.00 | -3.94 | -3.98 | -2.44 | -3.23 | -4.67 | MYB |
| Tai6.32284 | -2.99 | -1.75 | -2.36 | -6.82 | -4.36 | -6.08 | HB |
| Tai6.44809 | -2.94 | -3.85 | -2.99 | -2.22 | -2.68 | -3.71 | NAC |
| Tai6.2602 | -2.94 | -2.84 | -2.84 | -3.76 | -3.72 | -3.15 | AP2/ERF |
| Tai6.22045 | -2.93 | -2.19 | -3.18 | -1.76 | -1.06 | -1.70 | HB |
| Tai6.26652 | -2.90 | -4.81 | -5.94 | -5.36 | -2.14 | -3.89 | WRKY |
| Tai6.34684 | -2.90 | -2.62 | -1.61 | -2.81 | -1.29 | -4.95 | WRKY |
| Tai6.43076 | -2.82 | -4.14 | -4.23 | -2.40 | -4.23 | -5.75 | MYB |
| novel.212 | -2.82 | -3.27 | -3.81 | -3.64 | -2.84 | -5.82 | WRKY |
| Tai6.36831 | -2.81 | -2.11 | -3.14 | -4.21 | -3.98 | -5.27 | Tify |
| Tai6.55404 | -2.79 | -2.02 | -3.78 | -3.17 | -3.67 | -4.37 | HSF |
| Tai6.1831 | -2.79 | -3.95 | -5.75 | -2.91 | -2.91 | -9.28 | MYB |
| Tai6.54705 | -2.76 | -2.56 | -3.71 | -2.51 | -4.33 | -3.22 | MYB |
| Tai6.9566 | -2.75 | -1.09 | -2.56 | -3.45 | -3.01 | -2.78 | MYB |
| Tai6.28884 | -2.74 | -2.62 | -3.39 | -1.87 | -3.30 | -3.48 | HMG |
| Tai6.12432 | -2.73 | -2.02 | -3.77 | -3.80 | -5.04 | -3.19 | MYB |
| Tai6.25196 | -2.72 | -2.23 | -3.73 | -4.31 | -2.95 | -7.68 | WRKY |
| Tai6.55297 | -2.71 | -1.93 | -3.69 | -2.72 | -3.18 | -4.15 | HSF |
| Tai6.30157 | -2.71 | -1.52 | -3.10 | -4.28 | -2.95 | -3.06 | Tify |
| Tai6.21218 | -2.69 | -1.11 | -2.48 | -5.21 | -4.38 | -4.11 | MYB |
| Tai6.39639 | -2.67 | -2.91 | -4.50 | -3.55 | -2.60 | -7.73 | WRKY |
| Tai6.11744 | -2.67 | -1.77 | -4.93 | -3.05 | -4.94 | -3.53 | MYB |
| Tai6.22529 | -2.61 | -1.80 | -1.11 | -1.32 | -1.76 | -3.66 | NF-Y |
| Tai6.37772 | -2.60 | -1.66 | -3.42 | -2.71 | -3.62 | -3.34 | MYB |
| Tai6.19751 | -2.59 | -4.66 | -4.77 | -3.82 | -4.23 | -5.08 | NAC |
| Tai6.44645 | -2.58 | -2.96 | -4.33 | -6.05 | -6.08 | -6.08 | MYB |
| Tai6.31362 | -2.53 | -1.99 | -1.45 | -1.54 | -1.71 | -1.55 | HB |
| Tai6.48345 | -2.44 | -4.69 | -2.67 | -1.40 | -1.70 | -6.06 | NAC |
| Tai6.36227 | -2.43 | -2.99 | -3.65 | -2.95 | -2.58 | -2.64 | AP2/ERF |
| Tai6.6766 | -2.43 | -2.90 | -4.57 | -4.24 | -4.26 | -5.59 | NAC |
| Tai6.21644 | -2.36 | -2.71 | -4.45 | -5.13 | -5.64 | -6.14 | MYB |
| Tai6.5678 | -2.36 | -1.62 | -2.61 | -3.69 | -3.63 | -4.87 | Tify |
| Tai6.31360 | -2.36 | -1.22 | -1.22 | -1.87 | -1.97 | -2.17 | HB |
| Tai6.26672 | -2.32 | -2.46 | -3.23 | -1.10 | -1.40 | -1.92 | HB |
| Tai6.16515 | -2.21 | -2.03 | -3.33 | -2.63 | -3.64 | -4.40 | Tify |
| Tai6.28023 | -2.17 | -2.84 | -5.19 | -4.32 | -5.49 | -6.21 | WRKY |
| Tai6.32399 | -2.16 | -1.32 | -2.32 | -1.90 | -2.55 | -2.32 | HB |
| Tai6.41936 | -2.14 | -3.22 | -6.04 | -4.14 | -5.40 | -3.98 | MYB |
| Tai6.28127 | -2.13 | -3.96 | -6.07 | -3.97 | -6.60 | -6.73 | Tify |
| Tai6.5233 | -2.07 | -1.63 | -1.20 | -1.65 | -2.02 | -2.67 | C2H2 |
| Tai6.8842 | -2.05 | -2.15 | -2.64 | -1.90 | -2.33 | -2.98 | Trihelix |
| Tai6.51105 | -2.00 | -2.42 | -4.54 | -2.85 | -4.32 | -4.29 | GRAS |
| Tai6.33637 | -1.95 | -2.63 | -7.07 | -2.29 | -3.77 | -2.70 | C2H2 |
| Tai6.5889 | -1.95 | -1.18 | -2.13 | -1.93 | -1.96 | -2.20 | C2H2 |
| Tai6.47754 | -1.95 | -1.52 | -2.77 | -2.45 | -3.10 | -3.95 | Tify |
| Tai6.37238 | -1.92 | -3.37 | -3.85 | -1.32 | -3.38 | -3.94 | bZIP |
| Tai6.28018 | -1.91 | -2.74 | -5.54 | -4.82 | -6.07 | -8.31 | WRKY |
| Tai6.36172 | -1.90 | -2.41 | -4.54 | -5.83 | -5.44 | -5.87 | WRKY |
| Tai6.55057 | -1.89 | -2.70 | -5.23 | -4.45 | -5.20 | -5.07 | WRKY |
| Tai6.53538 | -1.88 | -6.50 | -7.47 | -4.46 | -4.63 | -2.89 | AP2/ERF |
| Tai6.29844 | -1.87 | -2.38 | -3.50 | -4.02 | -6.40 | -4.41 | AUX/IAA |
| Tai6.41937 | -1.84 | -1.64 | -2.81 | -1.92 | -1.77 | -1.24 | MYB |
| Tai6.44904 | -1.82 | -6.31 | -7.90 | -4.58 | -6.40 | -7.21 | AP2/ERF |
| Tai6.10712 | -1.82 | -4.12 | -4.27 | -2.26 | -3.55 | -5.39 | GARP |
| Tai6.12856 | -1.80 | -4.12 | -6.42 | -4.88 | -5.22 | -6.02 | MYB |
| Tai6.38873 | -1.79 | -3.47 | -5.52 | -5.27 | -5.40 | -5.51 | MYB |
| Tai6.48992 | -1.77 | -3.99 | -3.03 | -2.04 | -3.47 | -4.12 | WRKY |
| Tai6.33082 | -1.76 | -1.95 | -2.81 | -1.96 | -3.15 | -2.03 | HB |
| Tai6.11867 | -1.74 | -5.47 | -9.17 | -3.98 | -5.81 | -4.67 | AP2/ERF |
| Tai6.36951 | -1.74 | -3.07 | -3.68 | -2.99 | -1.67 | -2.72 | AP2/ERF |
| Tai6.15652 | -1.74 | -1.28 | -1.75 | -1.97 | -2.65 | -2.04 | Trihelix |
| Tai6.11610 | -1.73 | -1.49 | -1.55 | -2.25 | -2.96 | -4.93 | MYB |
| Tai6.37876 | -1.72 | -1.29 | -1.16 | -1.31 | -1.47 | -2.80 | NF-Y |
| Tai6.31939 | -1.71 | -1.22 | -3.13 | -2.91 | -4.16 | -5.47 | GRAS |
| Tai6.8517 | -1.70 | -3.30 | -5.34 | -3.94 | -6.32 | -6.22 | Tify |
| Tai6.16959 | -1.69 | -4.06 | -5.30 | -1.05 | -3.63 | -4.78 | AP2/ERF |
| Tai6.17504 | -1.68 | -2.18 | -3.49 | -1.02 | -2.61 | -2.89 | TRAF |
| Tai6.20995 | -1.66 | -1.01 | -1.37 | -1.00 | -1.09 | -1.45 | NF-Y |
| Tai6.16967 | -1.62 | -2.25 | -1.89 | -2.17 | -2.31 | -2.98 | MADS |
| Tai6.50677 | -1.59 | -2.11 | -4.16 | -3.74 | -4.70 | -4.20 | MYB |
| Tai6.7360 | -1.59 | -4.73 | -8.22 | -4.13 | -6.38 | -4.96 | AP2/ERF |
| Tai6.36949 | -1.57 | -3.09 | -4.50 | -1.62 | -4.66 | -4.89 | WRKY |
| Tai6.28246 | -1.57 | -2.69 | -5.60 | -2.21 | -4.29 | -2.26 | MYB |
| Tai6.11868 | -1.54 | -5.82 | -6.50 | -2.12 | -6.13 | -5.43 | AP2/ERF |
| Tai6.37832 | -1.54 | -5.02 | -7.49 | -3.94 | -6.04 | -4.60 | AP2/ERF |
| Tai6.28021 | -1.52 | -2.53 | -3.42 | -4.95 | -7.36 | -5.94 | WRKY |
| Tai6.31197 | -1.50 | -1.25 | -1.16 | -1.18 | -1.05 | -1.33 | C2H2 |
| Tai6.34961 | -1.50 | -1.35 | -1.32 | -1.52 | -2.34 | -2.84 | IWS1 |
| Tai6.35007 | -1.49 | -1.19 | -1.79 | -1.61 | -1.86 | -1.80 | HB |
| Tai6.32076 | -1.48 | -2.34 | -2.20 | -1.38 | -2.56 | -2.93 | NAC |
| Tai6.190 | -1.44 | -1.01 | -1.16 | -1.72 | -1.50 | -2.66 | NAC |
| Tai6.28289 | -1.44 | -1.64 | -2.70 | -2.10 | -2.22 | -2.15 | S1Fa-like |
| Tai6.9706 | -1.42 | -1.25 | -2.00 | -2.32 | -1.58 | -1.68 | C2H2 |
| Tai6.2771 | -1.41 | -2.60 | -4.46 | -1.34 | -4.38 | -4.71 | WRKY |
| Tai6.23287 | -1.39 | -1.88 | -4.14 | -2.37 | -3.75 | -3.60 | GRAS |
| Tai6.955 | -1.38 | -1.31 | -1.35 | -1.27 | -1.35 | -1.67 | TRAF |
| Tai6.51127 | -1.37 | -1.68 | -3.31 | -1.60 | -3.28 | -2.66 | Tify |
| Tai6.3250 | -1.33 | -4.06 | -3.72 | -1.23 | -1.72 | -2.66 | WRKY |
| Tai6.39511 | -1.33 | -1.48 | -2.24 | -1.72 | -1.12 | -2.70 | AUX/IAA |
| Tai6.9492 | -1.32 | -2.74 | -3.24 | -2.31 | -3.19 | -2.77 | MYB |
| Tai6.27144 | -1.32 | -2.70 | -4.59 | -3.37 | -4.71 | -3.46 | MYB |
| Tai6.50167 | -1.32 | -1.73 | -3.55 | -1.90 | -4.50 | -3.90 | WRKY |
| Tai6.40603 | -1.31 | -1.54 | -2.36 | -1.22 | -2.71 | -2.87 | NAC |
| Tai6.36173 | -1.31 | -1.76 | -3.08 | -5.43 | -4.40 | -5.46 | WRKY |
| Tai6.2653 | -1.30 | -4.06 | -4.13 | -2.06 | -2.51 | -3.34 | WRKY |
| Tai6.35278 | -1.30 | -1.63 | -1.21 | -1.67 | -1.82 | -2.05 | RWP-RK |
| Tai6.20291 | -1.29 | -3.77 | -5.40 | -4.19 | -2.54 | -4.22 | AP2/ERF |
| Tai6.17758 | -1.29 | -1.85 | -2.46 | -3.44 | -3.17 | -3.37 | WRKY |
| Tai6.13790 | -1.29 | -1.95 | -3.40 | -2.18 | -3.86 | -2.95 | Tify |
| Tai6.38342 | -1.28 | -1.11 | -1.06 | -1.10 | -2.05 | -1.40 | PHD |
| Tai6.10545 | -1.24 | -2.00 | -1.84 | -1.05 | -1.61 | -2.28 | HB |
| Tai6.55279 | -1.23 | -2.88 | -4.35 | -4.00 | -5.69 | -4.44 | WRKY |
| Tai6.458 | -1.21 | -1.24 | -1.05 | -1.62 | -1.20 | -1.97 | AP2/ERF |
| Tai6.5432 | -1.20 | -1.64 | -2.85 | -2.54 | -2.50 | -4.17 | WRKY |
| Tai6.3309 | -1.15 | -1.41 | -3.25 | -1.64 | -4.00 | -3.63 | WRKY |
| Tai6.21951 | -1.12 | -1.91 | -2.99 | -1.55 | -1.48 | -5.42 | AP2/ERF |
| Tai6.18182 | -1.11 | -1.58 | -2.55 | -1.99 | -1.78 | -5.41 | WRKY |
| Tai6.42054 | -1.09 | -3.07 | -4.93 | -2.49 | -2.40 | -3.49 | WRKY |
| Tai6.27259 | -1.09 | -3.13 | -3.39 | -2.11 | -2.69 | -3.59 | WRKY |
| Tai6.22767 | -1.08 | -2.18 | -2.91 | -2.85 | -2.14 | -3.28 | GARP |
| Tai6.39926 | -1.08 | -1.35 | -1.98 | -2.14 | -2.20 | -2.20 | HB |
| Tai6.30649 | -1.06 | -3.12 | -4.39 | -2.77 | -3.06 | -3.74 | WRKY |
| Tai6.44727 | -1.77 | -2.25 | -4.78 | -3.98 | -4.14 | -6.25 | NAC |
| Tai6.43947 | -1.60 | -1.32 | -1.05 | -1.45 | -1.27 | -1.55 | C2H2 |
| Tai6.38569 | -1.60 | -1.18 | -1.29 | -1.31 | -1.48 | -1.24 | SWI/SNF-BAF60b |
| Tai6.5041 | -1.23 | -3.71 | -2.02 | -3.98 | -3.50 | -1.76 | B3 |

**Table S12** GO enrichment analysis of green module gene.

| Category | ID | Term | pvalue | Gene Number |
| --- | --- | --- | --- | --- |
| BP | GO:0006099 | tricarboxylic acid cycle | 5.1133E-05 | 5 |
| BP | GO:0006101 | citrate metabolic process | 5.1133E-05 | 5 |
| BP | GO:0009060 | aerobic respiration | 5.1133E-05 | 5 |
| BP | GO:0015977 | carbon fixation | 5.1133E-05 | 5 |
| BP | GO:0015980 | energy derivation by oxidation of organic compounds | 0.000159142 | 5 |
| BP | GO:0045333 | cellular respiration | 0.000159142 | 5 |
| BP | GO:0072350 | tricarboxylic acid metabolic process | 0.000159142 | 5 |
| BP | GO:0016071 | mRNA metabolic process | 0.000308224 | 9 |
| BP | GO:0003333 | amino acid transmembrane transport | 0.000858026 | 6 |
| BP | GO:0006865 | amino acid transport | 0.000858026 | 6 |
| BP | GO:0098656 | anion transmembrane transport | 0.000858026 | 6 |
| BP | GO:1903825 | organic acid transmembrane transport | 0.000858026 | 6 |
| BP | GO:1905039 | carboxylic acid transmembrane transport | 0.000858026 | 6 |
| BP | GO:0071705 | nitrogen compound transport | 0.001016867 | 20 |
| BP | GO:0017038 | protein import | 0.001100914 | 4 |
| BP | GO:0006397 | mRNA processing | 0.001820807 | 7 |
| BP | GO:0000096 | sulfur amino acid metabolic process | 0.001894014 | 4 |
| BP | GO:0006888 | ER to Golgi vesicle-mediated transport | 0.001894014 | 4 |
| BP | GO:0071702 | organic substance transport | 0.002521072 | 20 |
| BP | GO:0009719 | response to endogenous stimulus | 0.002547548 | 7 |
| BP | GO:0009725 | response to hormone | 0.002547548 | 7 |
| BP | GO:0010033 | response to organic substance | 0.002547548 | 7 |
| BP | GO:0006094 | gluconeogenesis | 0.004198418 | 3 |
| BP | GO:0019319 | hexose biosynthetic process | 0.004198418 | 3 |
| BP | GO:0046364 | monosaccharide biosynthetic process | 0.004198418 | 3 |
| BP | GO:0048519 | negative regulation of biological process | 0.004516138 | 4 |
| BP | GO:0006352 | DNA-templated transcription, initiation | 0.006133808 | 6 |
| BP | GO:0019752 | carboxylic acid metabolic process | 0.007119269 | 25 |
| BP | GO:0010629 | negative regulation of gene expression | 0.007308084 | 3 |
| BP | GO:0006396 | RNA processing | 0.007610578 | 14 |
| BP | GO:0006082 | organic acid metabolic process | 0.008682824 | 25 |
| BP | GO:0043436 | oxoacid metabolic process | 0.008682824 | 25 |
| BP | GO:0006413 | translational initiation | 0.008715288 | 5 |
| BP | GO:0009892 | negative regulation of metabolic process | 0.009257975 | 3 |
| BP | GO:0010605 | negative regulation of macromolecule metabolic process | 0.009257975 | 3 |
| BP | GO:0015711 | organic anion transport | 0.009926321 | 6 |
| BP | GO:0015849 | organic acid transport | 0.009926321 | 6 |
| BP | GO:0046942 | carboxylic acid transport | 0.009926321 | 6 |
| BP | GO:0006520 | cellular amino acid metabolic process | 0.010243434 | 16 |
| BP | GO:0019318 | hexose metabolic process | 0.010878855 | 5 |
| BP | GO:0051641 | cellular localization | 0.011207393 | 13 |
| BP | GO:0008380 | RNA splicing | 0.011825247 | 4 |
| BP | GO:0008104 | protein localization | 0.013702078 | 15 |
| BP | GO:0046907 | intracellular transport | 0.013893731 | 12 |
| BP | GO:0051649 | establishment of localization in cell | 0.013893731 | 12 |
| BP | GO:0044700 | single organism signaling | 0.014164217 | 16 |
| BP | GO:0007165 | signal transduction | 0.015703323 | 16 |
| BP | GO:0023052 | signaling | 0.015703323 | 16 |
| BP | GO:0006091 | generation of precursor metabolites and energy | 0.016176174 | 9 |
| BP | GO:0015031 | protein transport | 0.017038498 | 14 |
| BP | GO:0015833 | peptide transport | 0.017038498 | 14 |
| BP | GO:0042886 | amide transport | 0.017038498 | 14 |
| BP | GO:0030001 | metal ion transport | 0.019422412 | 12 |
| BP | GO:0001522 | pseudouridine synthesis | 0.019443469 | 4 |
| BP | GO:0048193 | Golgi vesicle transport | 0.019443469 | 4 |
| BP | GO:0034220 | ion transmembrane transport | 0.019530482 | 10 |
| BP | GO:0045184 | establishment of protein localization | 0.020405825 | 14 |
| BP | GO:0006811 | ion transport | 0.021892621 | 24 |
| BP | GO:0072522 | purine-containing compound biosynthetic process | 0.023611376 | 6 |
| BP | GO:0005996 | monosaccharide metabolic process | 0.025121419 | 5 |
| BP | GO:0033036 | macromolecule localization | 0.029641037 | 15 |
| BP | GO:0018193 | peptidyl-amino acid modification | 0.030739136 | 6 |
| BP | GO:0016569 | covalent chromatin modification | 0.030833885 | 3 |
| BP | GO:0016570 | histone modification | 0.030833885 | 3 |
| BP | GO:0034613 | cellular protein localization | 0.033041869 | 11 |
| BP | GO:0070727 | cellular macromolecule localization | 0.033041869 | 11 |
| BP | GO:0006575 | cellular modified amino acid metabolic process | 0.03506825 | 3 |
| BP | GO:0000375 | RNA splicing, via transesterification reactions | 0.03958622 | 3 |
| BP | GO:0000377 | RNA splicing, via transesterification reactions with bulged adenosine as nucleophile | 0.03958622 | 3 |
| BP | GO:0000398 | mRNA splicing, via spliceosome | 0.03958622 | 3 |
| BP | GO:0006820 | anion transport | 0.039889137 | 8 |
| BP | GO:0051716 | cellular response to stimulus | 0.04130706 | 18 |
| BP | GO:0006886 | intracellular protein transport | 0.041370986 | 10 |
| BP | GO:0031124 | mRNA 3'-end processing | 0.045309721 | 2 |

**Table S13** KEGG enrichment analysis of green module gene.

| KEGGID | Term | pvalue | Gene Number |
| --- | --- | --- | --- |
| sot03022 | Basal transcription factors | 0.005952286 | 6 |
| sot03040 | Spliceosome | 0.006451269 | 14 |
| sot00770 | Pantothenate and CoA biosynthesis | 0.007868938 | 4 |
| sot04712 | Circadian rhythm - plant | 0.014344305 | 5 |
| sot00290 | Valine, leucine and isoleucine biosynthesis | 0.016524321 | 3 |
| sot00650 | Butanoate metabolism | 0.024004428 | 4 |
| sot03420 | Nucleotide excision repair | 0.035041383 | 6 |
| sot03430 | Mismatch repair | 0.042793883 | 4 |
| sot03015 | mRNA surveillance pathway | 0.05156637 | 8 |
| sot00230 | Purine metabolism | 0.076862254 | 7 |
| sot00620 | Pyruvate metabolism | 0.094218647 | 6 |
| sot03440 | Homologous recombination | 0.10617304 | 4 |
| sot03030 | DNA replication | 0.113329808 | 4 |
| sot00790 | Folate biosynthesis | 0.120627483 | 2 |
| sot00970 | Aminoacyl-tRNA biosynthesis | 0.120694965 | 4 |
| sot01210 | 2-Oxocarboxylic acid metabolism | 0.120694965 | 4 |
| sot00630 | Glyoxylate and dicarboxylate metabolism | 0.122260365 | 5 |
| sot04016 | MAPK signaling pathway - plant | 0.135046696 | 7 |
| sot00670 | One carbon pool by folate | 0.135389363 | 2 |
| sot03020 | RNA polymerase | 0.148442987 | 3 |
| sot04075 | Plant hormone signal transduction | 0.15266469 | 10 |
| sot03013 | RNA transport | 0.16144879 | 8 |
| sot03008 | Ribosome biogenesis in eukaryotes | 0.162527551 | 5 |
| sot02010 | ABC transporters | 0.197619812 | 2 |
| sot04120 | Ubiquitin mediated proteolysis | 0.234620266 | 6 |
| sot03060 | Protein export | 0.260881917 | 3 |
| sot03050 | Proteasome | 0.284741559 | 3 |
| sot00513 | Various types of N-glycan biosynthesis | 0.343696407 | 2 |
| sot03410 | Base excision repair | 0.343696407 | 2 |
| sot00900 | Terpenoid backbone biosynthesis | 0.344944042 | 3 |
| sot04933 | AGE-RAGE signaling pathway in diabetic complications | 0.357999236 | 1 |
| sot01230 | Biosynthesis of amino acids | 0.370612343 | 9 |
| sot00051 | Fructose and mannose metabolism | 0.380957419 | 3 |
| sot04146 | Peroxisome | 0.409617179 | 4 |
| sot00511 | Other glycan degradation | 0.412543579 | 1 |
| sot00310 | Lysine degradation | 0.421903229 | 2 |
| sot04144 | Endocytosis | 0.458621387 | 6 |
| sot00450 | Selenocompound metabolism | 0.462481554 | 1 |
| sot00400 | Phenylalanine, tyrosine and tryptophan biosynthesis | 0.466522137 | 2 |
| sot00460 | Cyanoamino acid metabolism | 0.466522137 | 2 |
| sot00710 | Carbon fixation in photosynthetic organisms | 0.496643765 | 3 |
| sot00010 | Glycolysis / Gluconeogenesis | 0.505533672 | 5 |
| sot00052 | Galactose metabolism | 0.509060436 | 2 |
| sot00020 | Citrate cycle (TCA cycle) | 0.622803807 | 2 |
| sot00600 | Sphingolipid metabolism | 0.623435144 | 1 |
| sot00100 | Steroid biosynthesis | 0.639840162 | 1 |
| sot00260 | Glycine, serine and threonine metabolism | 0.696493832 | 2 |
| sot00380 | Tryptophan metabolism | 0.698657148 | 1 |
| sot04136 | Autophagy - other | 0.698657148 | 1 |
| sot00220 | Arginine biosynthesis | 0.724380572 | 1 |
| sot04130 | SNARE interactions in vesicular transport | 0.736411891 | 1 |
| sot01200 | Carbon metabolism | 0.738706087 | 8 |
| sot03018 | RNA degradation | 0.756502775 | 3 |
| sot04145 | Phagosome | 0.765798069 | 2 |
| sot04141 | Protein processing in endoplasmic reticulum | 0.797447834 | 5 |
| sot00053 | Ascorbate and aldarate metabolism | 0.798394293 | 1 |
| sot00592 | alpha-Linolenic acid metabolism | 0.798394293 | 1 |
| sot00510 | N-Glycan biosynthesis | 0.807212381 | 1 |
| sot00040 | Pentose and glucuronate interconversions | 0.845874162 | 1 |
| sot00250 | Alanine, aspartate and glutamate metabolism | 0.845874162 | 1 |
| sot04070 | Phosphatidylinositol signaling system | 0.852627099 | 1 |
| sot00071 | Fatty acid degradation | 0.865263613 | 1 |
| sot00562 | Inositol phosphate metabolism | 0.871172088 | 1 |
| sot00030 | Pentose phosphate pathway | 0.876823083 | 1 |
| sot00240 | Pyrimidine metabolism | 0.901589743 | 1 |
| sot00520 | Amino sugar and nucleotide sugar metabolism | 0.920797252 | 2 |
| sot00561 | Glycerolipid metabolism | 0.94518524 | 1 |
| sot00480 | Glutathione metabolism | 0.949914481 | 1 |
| sot04626 | Plant-pathogen interaction | 0.951115043 | 2 |
| sot00190 | Oxidative phosphorylation | 0.98714294 | 1 |
| sot00500 | Starch and sucrose metabolism | 0.990654917 | 1 |
| sot03010 | Ribosome | 0.993556837 | 4 |

**Table S14** Genes with significant differences in tuberous root development between two varieties.

| Genes | Description | RGJ8_1vsRXGH_1_log2FoldChange | RGJ8_2vsRXGH_2_log2FoldChange | RGJ8_3vsRXGH_3_log2FoldChange | RGJ8_1vsRGJ8_0_log2FoldChange | RGJ8_2vsRGJ8_0_log2FoldChange | RGJ8_3vsRGJ8_0_log2FoldChange | RXGH_1vsRXGH_0_log2FoldChange | RXGH_2vsRXGH_0_log2FoldChange | RXGH_3vsRXGH_0_log2FoldChange |
| --- | --- | --- | --- | --- | --- | --- | --- | --- | --- | --- |
| Tai6.31062 | MYB | 11.38 | 6.19 | -0.57 | 4.88 | 3.19 | -0.59 | -3.98 | -0.75 | 2.31 |
| Tai6.45668 | MYB | -1.81 | -0.62 | 2.68 | -0.08 | 0.13 | 0.78 | 0.10 | -0.95 | -3.49 |
| Tai6.50925 | MYB | -1.24 | -3.62 | -1.74 | 0.36 | -2.00 | -2.90 | -0.65 | -0.70 | -3.38 |
| Tai6.11744 | MYB | 0.92 | 3.65 | -0.83 | -2.67 | -1.77 | -4.93 | -3.05 | -4.94 | -3.53 |
| Tai6.5873 | MYB | -4.79 | -5.34 | -3.23 | -1.00 | -1.24 | -0.09 | -0.21 | 0.01 | -0.81 |
| Tai6.7912 | MYB | -2.90 | -2.82 | 1.27 | -1.79 | -2.56 | -0.37 | 0.71 | -0.20 | -2.01 |
| Tai6.39273 | MYB | -1.61 | -1.54 | 2.75 | -1.64 | -2.17 | 0.10 | 0.86 | 0.19 | -1.73 |
| Tai6.50832 | MYB | -2.19 | -2.03 | -1.13 | -0.32 | 0.70 | 1.75 | 0.05 | 0.83 | 1.07 |
| Tai6.11727 | MYB | 0.68 | 1.45 | 2.58 | -0.26 | -0.53 | 0.87 | -0.71 | -1.81 | -1.46 |
| Tai6.6826 | MYB | 5.10 | 2.32 | 0.45 | 1.50 | -0.69 | -2.06 | -2.86 | -2.35 | -1.73 |
| Tai6.42596 | MYB | 1.21 | 0.63 | 2.38 | 1.05 | 1.01 | 1.87 | 0.79 | 1.26 | 0.50 |
| Tai6.44286 | MYB | 0.07 | 2.29 | 2.18 | -3.54 | -0.74 | -2.20 | -2.91 | -2.39 | -3.62 |
| Tai6.53844 | MYB | -2.02 | -2.39 | 4.76 | 1.01 | -1.85 | 1.84 | 3.24 | 0.67 | -2.64 |
| Tai6.38929 | MYB | 1.75 | 4.69 | 0.89 | -1.19 | -0.52 | -3.39 | -1.75 | -4.11 | -3.04 |
| Tai6.10006 | MYB | 2.30 | 1.69 | -1.13 | -3.96 | -3.70 | -6.24 | -6.44 | -5.63 | -5.26 |
| Tai6.48428 | MYB | 2.59 | 0.44 | 0.59 | 0.12 | -0.99 | -2.21 | -1.51 | -0.54 | -1.79 |
| Tai6.18134 | MYB | -2.63 | -3.77 | -2.10 | 1.21 | 0.72 | 2.24 | 3.35 | 3.93 | 3.87 |
| Tai6.48937 | MYB | 3.06 | 3.11 | 1.65 | -0.32 | -2.43 | -3.40 | -1.95 | -4.19 | -3.58 |
| Tai6.13297 | MYB | 3.92 | 3.73 | 1.41 | 1.40 | 1.09 | 0.10 | NA | NA | 3.04 |
| Tai6.36389 | MYB | 2.92 | 1.81 | 2.47 | 1.06 | 1.21 | 2.06 | 0.38 | 1.57 | 1.85 |
| Tai6.44645 | MYB | 2.12 | 1.68 | 0.44 | -2.58 | -2.96 | -4.33 | -6.05 | -6.08 | -6.08 |
| Tai6.37772 | MYB | 1.68 | 3.46 | 1.55 | -2.60 | -1.66 | -3.42 | -2.71 | -3.62 | -3.34 |
| Tai6.41936 | MYB | 3.45 | 3.56 | -0.55 | -2.14 | -3.22 | -6.04 | -4.14 | -5.40 | -3.98 |
| Tai6.34267 | MYB | -0.05 | -2.67 | -3.41 | 0.72 | 0.31 | -1.24 | -0.69 | 1.44 | 0.73 |
| Tai6.14802 | MYB | -2.68 | -1.51 | -0.36 | -0.88 | 0.38 | 1.23 | -0.57 | -0.55 | -0.77 |
| Tai6.37982 | MYB | -4.01 | -4.43 | -2.32 | -1.41 | -1.65 | -0.62 | 0.16 | 0.26 | -0.70 |
| Tai6.27144 | MYB | 4.63 | 4.52 | 1.51 | -1.32 | -2.70 | -4.59 | -3.37 | -4.71 | -3.46 |
| Tai6.52916 | MYB | 2.37 | 3.07 | 3.51 | 0.45 | 0.16 | -0.01 | 3.19 | 2.16 | 1.68 |
| Tai6.50497 | MYB | 4.23 | -0.84 | -1.76 | 1.79 | -0.91 | -2.26 | -3.33 | -1.01 | -1.35 |
| Tai6.33690 | MYB | 5.18 | 1.48 | -0.63 | 0.65 | 0.04 | -0.54 | -1.93 | 1.09 | 2.73 |
| Tai6.43737 | MYB | -0.52 | -3.90 | 1.21 | 1.85 | 0.48 | 3.93 | 1.90 | 3.84 | 2.24 |
| Tai6.24407 | MYB | -5.28 | -7.22 | 3.98 | NA | NA | 3.99 | 3.56 | 3.51 | -3.29 |
| Tai6.5606 | MYB | -0.95 | -1.23 | -2.47 | 0.03 | 0.77 | 0.01 | -1.68 | -0.73 | -0.16 |
| Tai6.43739 | MYB | -2.61 | -3.54 | 0.63 | 0.67 | 1.18 | 3.95 | 2.15 | 3.48 | 2.15 |
| Tai6.28246 | MYB | 3.25 | 4.21 | -0.73 | -1.57 | -2.69 | -5.60 | -2.21 | -4.29 | -2.26 |
| Tai6.6204 | MYB | 0.97 | 3.65 | 0.09 | -1.56 | -0.94 | -3.82 | -2.02 | -4.16 | -3.34 |
| Tai6.21218 | MYB | 1.90 | 2.56 | 1.06 | -2.69 | -1.11 | -2.48 | -5.21 | -4.38 | -4.11 |
| Tai6.54254 | MYB | 6.43 | 5.89 | 3.56 | 0.23 | -0.46 | -0.81 | NA | NA | NA |
| Tai6.2734 | MYB | 2.30 | 3.12 | 3.80 | -1.14 | -1.27 | 1.72 | 2.57 | NA | 3.96 |
| Tai6.28396 | MYB | 1.83 | 2.67 | 1.38 | -3.55 | -0.27 | -2.40 | -3.75 | -1.41 | -2.10 |
| Tai6.42166 | MYB | -2.52 | -3.22 | 0.88 | -1.79 | -3.12 | -0.40 | 0.88 | 0.18 | -1.11 |
| Tai6.51056 | MYB | 2.41 | 0.95 | 0.07 | 0.52 | -1.27 | -1.14 | -0.95 | -1.35 | -0.25 |
| Tai6.42605 | MYB | 4.20 | 0.02 | -0.86 | 2.12 | -0.09 | -1.12 | -2.44 | -0.54 | -0.59 |
| Tai6.2271 | MYB | 2.03 | -0.49 | -0.86 | 0.72 | 0.19 | -1.61 | -0.24 | 1.68 | 0.34 |
| Tai6.7968 | MYB | 1.98 | -1.23 | 5.80 | -3.23 | -5.19 | -4.17 | -3.47 | -2.32 | -7.71 |
| Tai6.31218 | MYB | -2.28 | -3.37 | -1.33 | 1.19 | 0.90 | 2.31 | 2.43 | 3.16 | 2.62 |
| Tai6.24408 | MYB | -1.55 | -2.70 | 2.13 | -0.87 | -2.68 | -2.05 | 0.03 | -0.70 | -4.80 |
| Tai6.19655 | MYB | 9.37 | 4.03 | -1.74 | 5.94 | 4.30 | 0.19 | -3.10 | 0.51 | 2.28 |
| Tai6.44686 | MYB | 5.02 | 2.99 | 0.20 | 1.39 | -1.23 | -3.22 | -2.64 | -3.32 | -2.38 |
| Tai6.11326 | MYB | -2.69 | -3.04 | -2.17 | 1.16 | 0.97 | 2.49 | -1.76 | -1.71 | -0.96 |
| Tai6.54469 | MYB | -7.22 | -5.08 | -0.75 | -2.42 | -1.39 | 1.86 | 2.75 | 1.53 | 0.62 |
| Tai6.29343 | MYB | 6.08 | 10.07 | 7.97 | -3.07 | 0.77 | -1.55 | NA | NA | NA |
| Tai6.54255 | MYB | 4.14 | 1.42 | -0.92 | 0.33 | -0.16 | -1.10 | -2.14 | 0.01 | 1.53 |
| Tai6.13577 | MYB | -2.43 | -2.66 | 0.74 | 0.48 | 0.21 | 3.14 | 0.59 | 0.44 | 0.09 |
| Tai6.20609 | MYB | 0.53 | 1.61 | 2.26 | -0.01 | 0.94 | 2.27 | 1.37 | 1.17 | 1.94 |
| Tai6.11610 | MYB | 1.21 | 2.09 | 4.13 | -1.73 | -1.49 | -1.55 | -2.25 | -2.96 | -4.93 |
| Tai6.24680 | MYB | 6.21 | 3.48 | 1.60 | 1.83 | -0.37 | -1.88 | -2.88 | -2.41 | -1.92 |
| Tai6.38873 | MYB | 4.33 | 2.71 | 0.85 | -1.79 | -3.47 | -5.52 | -5.27 | -5.40 | -5.51 |
| Tai6.869 | MYB | 2.70 | 1.37 | 0.97 | 1.27 | -0.40 | 0.53 | -0.07 | -0.50 | 0.94 |
| Tai6.22164 | MYB | 2.79 | -1.61 | -1.67 | 1.15 | -2.47 | -2.85 | 0.05 | 0.73 | 0.52 |
| Tai6.7417 | MYB | -5.22 | -3.34 | NA | NA | NA | NA | 3.04 | 0.99 | NA |
| Tai6.39174 | MYB | 3.75 | NA | 5.39 | -0.91 | -3.02 | 0.40 | NA | NA | NA |
| Tai6.51885 | MYB | 10.58 | 10.58 | 8.89 | 3.28 | 3.15 | 1.24 | NA | NA | NA |
| Tai6.2041 | MYB | -2.49 | -1.68 | -1.62 | -1.25 | -0.37 | 0.38 | 0.61 | 0.61 | 1.41 |
| Tai6.18013 | MYB | 1.39 | 2.25 | -0.20 | -0.40 | -1.27 | -2.28 | -1.86 | -3.65 | -2.12 |
| Tai6.23945 | MYB | 1.83 | 0.99 | 3.03 | -0.88 | -3.37 | -3.11 | 0.48 | -1.24 | -2.88 |
| Tai6.12397 | MYB | 1.98 | 2.70 | 2.06 | 0.58 | 0.93 | 1.14 | -0.12 | -0.56 | 0.38 |
| Tai6.10658 | MYB | -0.14 | -0.70 | 2.40 | 0.60 | 1.01 | 3.18 | -1.63 | -0.73 | -1.54 |
| Tai6.12856 | MYB | 4.17 | 2.12 | 0.72 | -1.80 | -4.12 | -6.42 | -4.88 | -5.22 | -6.02 |
| Tai6.44154 | MYB | -2.28 | -2.95 | 2.71 | -1.71 | -3.57 | -0.73 | 0.95 | -0.31 | -3.03 |
| Tai6.52268 | MYB | 2.60 | 1.90 | 0.53 | -0.84 | -1.13 | -3.00 | -1.86 | -1.49 | -1.89 |
| Tai6.28005 | MYB | -2.81 | -2.33 | -2.48 | 0.12 | 0.89 | 0.64 | -0.13 | 0.08 | 0.07 |
| Tai6.21198 | MYB | 2.15 | 2.15 | 0.82 | 0.22 | -1.70 | -2.38 | -1.61 | -3.61 | -2.85 |
| Tai6.44382 | MYB | 4.93 | 1.82 | -0.56 | 0.84 | 0.03 | -0.49 | -2.69 | -0.47 | 1.51 |
| Tai6.52652 | MYB | -7.36 | -4.06 | -1.97 | -2.62 | 0.74 | 1.16 | 7.13 | 7.13 | 5.47 |
| Tai6.31061 | MYB | 9.46 | 5.44 | -1.51 | 6.27 | 4.83 | -0.61 | NA | 2.46 | 3.94 |
| Tai6.12271 | MYB | -1.83 | -2.24 | -0.79 | 0.22 | 1.31 | 2.82 | -0.74 | 0.68 | 0.83 |
| Tai6.38491 | MYB | -2.01 | -0.29 | -1.63 | -2.07 | -1.58 | -3.20 | -0.77 | -2.07 | -2.26 |
| Tai6.7144 | MYB | -2.69 | -2.61 | -0.03 | -0.98 | -0.35 | 1.23 | 0.53 | 1.02 | 0.09 |
| Tai6.31724 | MYB | -3.81 | -4.50 | -3.54 | 0.55 | 0.03 | 0.92 | 0.03 | 0.14 | 0.15 |
| Tai6.47905 | MYB | 1.97 | 1.04 | 2.33 | 1.80 | 1.46 | 2.09 | 1.50 | 2.02 | 1.49 |
| Tai6.45825 | MYB | 2.55 | -0.31 | 0.45 | 0.84 | -1.26 | -0.95 | -1.07 | -0.39 | -0.74 |
| Tai6.51693 | MYB | 1.29 | 1.96 | 2.84 | -0.92 | -0.35 | 0.53 | -0.31 | -0.49 | -0.40 |
| Tai6.44285 | MYB | -6.02 | -4.82 | -4.99 | -3.92 | -4.03 | -4.22 | 0.74 | -0.67 | -0.58 |
| Tai6.32321 | MYB | -5.49 | -3.04 | -1.43 | -3.33 | -0.83 | 0.61 | -0.47 | -0.51 | -0.59 |
| novel.272 | MYB | 2.36 | 1.50 | 3.99 | -1.45 | -0.76 | -1.28 | -1.87 | -0.41 | -3.17 |
| Tai6.46159 | MYB | -1.65 | -2.57 | -3.94 | 1.32 | 1.21 | -0.74 | 0.40 | 1.12 | 0.68 |
| Tai6.23332 | MYB | -0.90 | -1.78 | -2.77 | 1.76 | 1.37 | -0.31 | 0.50 | 0.89 | 0.33 |
| Tai6.33480 | bHLH | 1.07 | 6.12 | 3.90 | -0.45 | -1.10 | -1.35 | 0.92 | -4.47 | -2.65 |
| Tai6.17666 | bHLH | -3.14 | -4.99 | -4.19 | 2.36 | 1.81 | 3.02 | 0.58 | 1.75 | 2.16 |
| Tai6.1919 | bHLH | 0.51 | 0.40 | 2.46 | 0.44 | 0.22 | 1.80 | 1.34 | 1.17 | 0.77 |
| Tai6.27475 | bHLH | 9.20 | 2.15 | -0.77 | 7.36 | 6.33 | 4.09 | NA | 5.73 | 6.44 |
| Tai6.31167 | bHLH | 0.59 | 0.89 | 2.46 | -1.24 | -2.37 | -2.12 | -2.56 | -4.05 | -5.25 |
| Tai6.28734 | bHLH | 13.88 | 5.94 | 5.44 | 2.86 | 1.49 | -0.40 | -4.88 | 1.39 | 0.13 |
| Tai6.20663 | bHLH | 2.42 | 1.83 | 1.37 | 1.34 | 0.98 | 0.70 | -0.12 | 0.03 | 0.32 |
| Tai6.36587 | bHLH | -1.90 | -2.38 | -1.23 | 0.44 | 0.62 | 1.67 | 1.07 | 1.67 | 1.66 |
| Tai6.27510 | bHLH | 0.26 | 2.20 | 0.85 | -1.52 | -0.55 | -1.81 | -1.21 | -2.24 | -2.06 |
| Tai6.37579 | bHLH | -1.43 | 1.79 | 5.80 | -2.00 | -2.74 | -1.76 | 4.45 | 0.48 | NA |
| Tai6.26376 | bHLH | -2.10 | -2.58 | -0.69 | 0.12 | 0.20 | 0.29 | -0.04 | 0.44 | -1.26 |
| Tai6.6972 | bHLH | 4.25 | 1.78 | -0.15 | 5.46 | 5.22 | 5.25 | 1.63 | 3.76 | 5.86 |
| Tai6.25261 | bHLH | -2.02 | -2.81 | -1.90 | 0.71 | 0.73 | 1.60 | 0.78 | 1.52 | 1.59 |
| Tai6.44305 | bHLH | -2.18 | -2.10 | -1.61 | 0.83 | 1.44 | 2.43 | 0.92 | 1.37 | 1.97 |
| Tai6.16987 | bHLH | 4.68 | 4.90 | -0.01 | 0.73 | 0.80 | -0.79 | NA | NA | 1.05 |
| Tai6.15420 | bHLH | 2.97 | 0.13 | -0.20 | 1.17 | -1.46 | -0.76 | 0.73 | 0.85 | 1.98 |
| Tai6.5084 | bHLH | 5.35 | 6.42 | 3.42 | -0.60 | 0.70 | -1.93 | -2.70 | -2.55 | -2.06 |
| Tai6.30613 | bHLH | -3.31 | -1.74 | -2.61 | -1.80 | -1.62 | -2.35 | 0.90 | -0.57 | -0.33 |
| Tai6.33665 | bHLH | 7.55 | 4.61 | 3.17 | 2.79 | 1.52 | -0.27 | -0.65 | 0.96 | 0.73 |
| Tai6.23420 | bHLH | 0.16 | 0.23 | 2.56 | 0.26 | -0.81 | -0.09 | 1.11 | -0.10 | -1.58 |
| Tai6.29234 | bHLH | 4.45 | 1.86 | 0.35 | -0.13 | -1.17 | -1.86 | -1.59 | -0.14 | 0.79 |
| Tai6.42247 | bHLH | 9.30 | 6.38 | 5.45 | 2.98 | 2.01 | 0.16 | -1.19 | 0.69 | -0.09 |
| Tai6.40550 | bHLH | 2.52 | 2.49 | 2.17 | 0.20 | 0.57 | 0.87 | -1.82 | -1.51 | -0.80 |
| Tai6.48463 | bHLH | 2.36 | 1.54 | 1.08 | 0.04 | -1.45 | -2.55 | -0.42 | -1.18 | -1.71 |
| Tai6.32052 | bHLH | -1.75 | -2.24 | -0.29 | 0.58 | 0.15 | 1.54 | 1.08 | 1.09 | 0.61 |
| Tai6.1459 | bHLH | 2.30 | 1.19 | 2.61 | 1.37 | -0.21 | 1.21 | -1.23 | -1.76 | -1.68 |
| Tai6.53726 | bHLH | 4.32 | 1.84 | 0.20 | -0.21 | -1.35 | -2.32 | -1.33 | -0.07 | 0.69 |
| Tai6.40984 | bHLH | 2.16 | 4.27 | -0.42 | 0.35 | 1.63 | -2.28 | -0.40 | -1.30 | -0.42 |
| Tai6.52011 | bHLH | 2.93 | 2.48 | 2.97 | 1.12 | 1.40 | 1.79 | -0.18 | 0.48 | 0.48 |
| Tai6.29538 | bHLH | -0.80 | 2.95 | 0.29 | 0.35 | 1.49 | 0.30 | 0.14 | -2.51 | -0.97 |
| Tai6.21150 | bHLH | -2.43 | -1.68 | -0.79 | -0.39 | -0.89 | -0.34 | 0.16 | -1.15 | -1.40 |
| Tai6.23189 | bHLH | 4.93 | 2.49 | -0.59 | 4.18 | 4.97 | 3.02 | -0.19 | 2.91 | 4.11 |
| Tai6.26250 | bHLH | 1.26 | 2.32 | 0.99 | -0.73 | 0.38 | -0.37 | -1.07 | -1.08 | -0.41 |
| Tai6.48482 | bHLH | 3.71 | 1.10 | -0.62 | 5.02 | 4.65 | 4.63 | 2.49 | 4.61 | 6.47 |
| Tai6.17660 | bHLH | -1.41 | -2.85 | -2.29 | 2.48 | 2.55 | 2.72 | 2.04 | 3.45 | 3.19 |
| Tai6.11337 | bHLH | 5.79 | 6.02 | 7.51 | 0.89 | 1.03 | 1.84 | -0.09 | -0.23 | -0.82 |
| Tai6.9341 | bHLH | 3.06 | 6.33 | 7.37 | -0.79 | 0.35 | -1.61 | 0.55 | -1.64 | -4.03 |
| Tai6.31899 | bHLH | 6.28 | 2.76 | 0.60 | 1.79 | 0.36 | -1.80 | -2.25 | -0.26 | -0.15 |
| Tai6.20661 | bHLH | 4.14 | 3.36 | 2.56 | 1.02 | 0.50 | 0.02 | -1.50 | -1.33 | -0.90 |
| Tai6.45123 | bHLH | 3.85 | 1.87 | -0.56 | 2.50 | 2.08 | 1.68 | 0.79 | 2.27 | 4.41 |
| Tai6.39723 | bHLH | 8.08 | 1.39 | -2.08 | 8.99 | 7.46 | 4.41 | 2.56 | 7.60 | 8.03 |
| Tai6.38308 | bHLH | 3.47 | 3.09 | 2.24 | 0.67 | 0.28 | 0.82 | -0.54 | -0.61 | 0.88 |
| Tai6.6746 | bHLH | -2.83 | -2.45 | -0.79 | -0.20 | 0.74 | 2.18 | 0.90 | 1.40 | 1.24 |
| Tai6.42935 | bHLH | 3.61 | 2.67 | 4.24 | 0.89 | 0.47 | 1.67 | 1.47 | 1.98 | 1.74 |
| Tai6.47392 | bHLH | 2.36 | 1.31 | 2.18 | 0.84 | 0.21 | 1.17 | 0.63 | 0.99 | 1.20 |
| Tai6.40705 | bHLH | -3.73 | -3.55 | -0.57 | 1.21 | 1.41 | 2.60 | 0.56 | 0.49 | -1.26 |
| Tai6.38469 | bHLH | 4.70 | 4.18 | 7.09 | -0.33 | -1.76 | -1.04 | -1.68 | -2.59 | -4.24 |
| Tai6.16969 | bHLH | NA | -4.80 | -4.25 | -4.45 | -4.56 | -4.75 | -5.58 | -3.00 | -3.66 |
| Tai6.28154 | bHLH | 3.37 | 3.33 | 3.29 | 0.46 | 0.46 | -0.13 | -2.42 | -2.46 | -2.89 |
| Tai6.33571 | bHLH | 1.22 | 2.52 | 1.20 | -0.67 | 0.41 | -0.28 | -1.18 | -1.46 | -0.74 |
| Tai6.4030 | bHLH | -0.13 | -2.87 | 0.27 | 0.55 | -2.04 | -0.89 | -0.74 | -0.66 | -2.53 |
| Tai6.21148 | bHLH | -3.57 | -1.10 | -0.69 | -0.46 | -0.23 | 0.54 | 1.68 | -0.61 | -0.19 |
| Tai6.28736 | bHLH | 7.84 | 5.31 | 4.77 | 3.00 | 1.82 | -0.17 | 0.51 | 1.82 | 0.52 |
| Tai6.50538 | bHLH | 2.03 | 0.01 | 0.67 | 0.49 | -2.23 | -0.47 | -0.22 | -1.02 | 0.22 |
| Tai6.6656 | bHLH | 1.09 | 0.19 | 2.34 | -0.15 | -1.51 | -0.87 | -0.15 | -0.69 | -2.06 |
| Tai6.20566 | bHLH | -2.63 | -0.70 | -1.76 | -2.47 | -2.36 | -3.44 | 0.68 | -1.21 | -1.14 |
| Tai6.44623 | bHLH | 1.57 | 2.38 | 5.96 | 1.58 | 0.87 | 2.41 | 1.13 | -0.47 | -2.31 |
| Tai6.701 | bHLH | -3.09 | -3.27 | 3.43 | -0.56 | -3.80 | -0.56 | 2.32 | -0.73 | -3.62 |
| Tai6.8091 | bHLH | 1.19 | 2.39 | 1.44 | -0.52 | 0.29 | -1.59 | -1.08 | -1.54 | -2.36 |
| Tai6.53559 | bHLH | -2.27 | -5.52 | -4.47 | 0.14 | -3.80 | -3.97 | 3.84 | 3.12 | 1.99 |
| Tai6.36476 | bHLH | 2.27 | 0.30 | 2.40 | 0.97 | -0.28 | 1.61 | 0.07 | 0.74 | 0.63 |
| Tai6.25160 | bHLH | 2.44 | 0.19 | -1.21 | 6.18 | 5.78 | 6.10 | 3.31 | 5.04 | 6.90 |
| Tai6.6151 | bHLH | 4.83 | 4.40 | 2.59 | 2.33 | 2.56 | 2.00 | -0.02 | 0.56 | 1.90 |
| Tai6.7420 | bHLH | -1.85 | -0.68 | -2.01 | 0.42 | 1.82 | 0.27 | 1.42 | 1.57 | 1.45 |
| Tai6.38167 | bHLH | 9.89 | 3.50 | 1.15 | 9.95 | 8.13 | 6.57 | NA | 2.23 | 2.91 |
| Tai6.40757 | bHLH | -3.38 | -7.26 | -3.36 | 1.64 | NA | 2.20 | 1.25 | 1.44 | 1.70 |
| Tai6.41328 | bHLH | 2.41 | 2.20 | 3.50 | 0.44 | -0.64 | 0.43 | -0.46 | -1.37 | -1.51 |
| Tai6.42698 | bHLH | -1.54 | -2.48 | -0.71 | -0.15 | 0.71 | 1.16 | -1.64 | 0.09 | -1.15 |
| Tai6.8220 | bHLH | -2.13 | -2.55 | -1.67 | -0.96 | -1.17 | -0.75 | 0.56 | 0.71 | 0.34 |
| Tai6.5404 | bHLH | -2.47 | -1.32 | 0.72 | -1.43 | -2.14 | -0.74 | -1.23 | -3.14 | -3.68 |
| Tai6.407 | bHLH | 3.11 | 4.44 | 3.40 | -0.27 | -1.00 | -0.33 | -0.03 | -2.04 | -0.28 |
| Tai6.43997 | bHLH | 2.50 | 1.69 | 1.59 | -0.20 | -0.30 | -0.14 | 0.17 | 0.81 | 1.15 |
| Tai6.27983 | bHLH | 3.53 | 3.25 | 3.63 | 0.16 | 0.76 | 1.01 | -2.67 | -1.86 | -1.90 |
| Tai6.6703 | bHLH | 2.36 | 1.82 | 0.86 | 0.53 | -0.13 | -0.63 | -1.33 | -1.50 | -0.95 |
| Tai6.24951 | bHLH | -1.56 | -2.56 | -0.39 | -0.16 | 0.69 | 1.45 | -1.28 | 0.51 | -0.82 |
| Tai6.14855 | bHLH | 2.35 | 2.07 | 1.05 | 1.33 | 0.52 | 0.04 | 0.51 | -0.11 | 0.55 |
| Tai6.37610 | bHLH | 9.00 | 4.93 | 3.03 | 2.63 | 1.19 | -0.71 | -2.74 | -0.35 | -0.23 |
| Tai6.7827 | bHLH | 2.10 | 1.64 | 0.98 | 0.90 | 0.59 | 0.46 | -1.00 | -0.91 | -0.29 |
| Tai6.6702 | bHLH | 2.26 | 1.87 | 1.18 | 0.92 | 0.68 | 0.79 | -1.46 | -1.37 | -0.47 |
| Tai6.29733 | bHLH | 2.05 | 0.10 | -1.29 | 1.57 | 0.76 | 0.08 | 0.08 | 1.13 | 1.96 |
| Tai6.44574 | bHLH | -7.08 | -8.81 | -8.96 | NA | NA | NA | -0.15 | 1.35 | 1.40 |
| Tai6.10491 | bHLH | 1.16 | 2.74 | 1.18 | -0.91 | -0.97 | -1.93 | -1.53 | -3.24 | -2.52 |
| Tai6.44288 | bHLH | 0.23 | 3.40 | 2.02 | -5.35 | -1.23 | -3.26 | -5.07 | -4.19 | -4.71 |
| Tai6.42781 | bHLH | 3.02 | 1.41 | 0.19 | 1.96 | 1.27 | 0.51 | 0.53 | 1.36 | 1.93 |
| Tai6.35040 | bHLH | 3.62 | 3.24 | 3.16 | 0.95 | 0.81 | 1.05 | 0.35 | 0.51 | 0.94 |
| Tai6.5402 | bHLH | 2.69 | 4.53 | 5.40 | -1.08 | -1.51 | -1.87 | -0.15 | -2.46 | -3.53 |
| Tai6.28799 | WD40 | 4.07 | 6.37 | 5.73 | -0.64 | -0.45 | -1.27 | NA | NA | NA |
| Tai6.46208 | WD40 | -6.30 | -4.76 | -4.79 | -1.72 | -0.71 | -1.17 | -0.40 | -0.99 | -1.33 |
| Tai6.10898 | WD40 | -3.63 | -3.92 | -4.58 | -0.99 | -0.96 | -1.26 | 0.67 | 0.93 | 1.39 |
| Tai6.43683 | C4H | 3.78 | 4.62 | 0.86 | 1.05 | 1.49 | -2.80 | -2.67 | -3.13 | -3.58 |
| Tai6.20405 | C4H | -8.03 | -11.7 | -7.10 | -1.38 | -3.32 | -0.22 | -0.19 | 1.46 | 0.03 |
| Tai6.31820 | C4H | 3.32 | 3.59 | 0.52 | 0.71 | 1.37 | -1.91 | -3.35 | -3.03 | -3.13 |
| Tai6.7553 | C4H | 4.25 | 2.12 | 2.64 | 2.92 | 1.34 | -0.46 | -1.55 | -1.05 | -3.20 |
| Tai6.31403 | C4H | 3.89 | 4.09 | 0.47 | 2.90 | 1.21 | -2.41 | -0.69 | -2.62 | -2.52 |
| Tai6.20404 | C4H | -8.77 | -9.67 | -7.83 | NA | NA | NA | 0.06 | 0.74 | -1.20 |
| Tai6.23786 | 4CL | 2.92 | 3.42 | 3.35 | 0.86 | 0.39 | -0.53 | 0.64 | -0.33 | -1.03 |
| Tai6.46820 | 4CL | 1.38 | 2.28 | 0.54 | -0.31 | 0.33 | -0.27 | 0.44 | 0.12 | 1.36 |
| Tai6.3857 | 4CL | -5.35 | -2.53 | NA | -3.53 | -1.86 | -3.82 | 3.15 | 1.91 | NA |
| Tai6.587 | 4CL | 9.58 | 3.04 | 4.08 | 2.49 | -0.52 | -3.38 | NA | 3.31 | NA |
| novel.273 | 4CL | -0.55 | -4.21 | -2.60 | 2.18 | -1.42 | -0.97 | 0.93 | 0.90 | -0.14 |
| Tai6.54279 | 4CL | 2.51 | 2.20 | 2.05 | 1.22 | 1.34 | 0.66 | -0.79 | -0.43 | -0.84 |
| Tai6.586 | 4CL | 8.57 | 1.27 | 3.33 | 3.11 | -0.35 | -2.44 | NA | 3.59 | NA |
| Tai6.8930 | 4CL | 3.53 | 5.41 | 3.74 | 1.47 | 1.62 | 1.76 | 0.67 | -1.12 | 0.77 |
| Tai6.8922 | 4CL | 4.77 | 4.44 | 4.47 | 0.10 | -0.32 | -0.53 | -1.65 | -1.77 | -1.91 |
| Tai6.37076 | 4CL | -2.33 | -0.80 | -0.72 | -0.50 | 0.32 | 0.74 | 0.67 | -0.10 | 0.32 |
| Tai6.54351 | 4CL | 7.63 | 4.00 | NA | 2.91 | -0.81 | -5.02 | NA | NA | NA |
| Tai6.23346 | 4CL | 8.07 | 4.91 | NA | 3.16 | -0.10 | -3.49 | NA | NA | NA |
| Tai6.46186 | CHS | 4.95 | 4.24 | -0.55 | 2.35 | 1.54 | -1.26 | NA | NA | NA |
| Tai6.10232 | CHS | 2.06 | -0.98 | -2.00 | 2.88 | 1.51 | 0.57 | -1.14 | 0.47 | 0.62 |
| Tai6.1734 | CHS | 7.44 | 4.79 | 2.58 | 4.11 | 3.80 | 0.41 | 0.72 | 3.01 | 1.96 |
| Tai6.53503 | CHS | 10.49 | 6.40 | 6.27 | 3.80 | 2.89 | -0.76 | NA | 2.98 | NA |
| Tai6.45381 | CHS | 8.19 | 5.50 | 3.33 | 3.80 | 3.18 | -0.02 | 0.47 | 2.47 | 1.57 |
| Tai6.1120 | CHS | 3.26 | 0.62 | -2.12 | 1.72 | 0.15 | -1.81 | -0.25 | 0.74 | 1.61 |
| Tai6.1732 | CHS | 9.80 | 4.99 | 3.10 | 3.09 | 2.75 | -0.97 | -3.27 | 0.91 | -0.76 |
| Tai6.29634 | CHS | 4.56 | 3.34 | NA | 2.73 | 1.41 | -0.39 | NA | NA | NA |
| Tai6.32804 | CHIL | 10.49 | 3.97 | 0.52 | 2.52 | 1.73 | -3.15 | -3.94 | 1.49 | 0.18 |
| Tai6.7605 | CHIL | 8.47 | 4.22 | -0.33 | 3.17 | 2.47 | -2.43 | NA | 1.30 | 1.05 |
| Tai6.40394 | LDOX/ANS | 9.42 | 7.65 | 5.90 | 4.34 | 3.76 | 0.95 | -0.46 | 0.67 | -0.27 |
| Tai6.42091 | LDOX/ANS | 9.89 | 6.46 | 4.26 | 4.08 | 3.67 | 0.98 | -0.57 | 2.37 | 2.01 |

**Table S15** Primer sequences of qRT-PCR

| Primer name | Primer sequence(5'−3') |
| --- | --- |
| Tai6.24971-F | CAAAGGCACAGGTGGTAGGA |
| Tai6.24971-R | CTTCGGTCTTGGGAGGATTAG |
| Tai6.46822-F | ACCCAGAGTGCGTGGATACT |
| Tai6.46822-R | TCTGAAGGGATGACAGGTGG |
| Tai6.3107-F | AGACTCTTGGCAACGGTAACG |
| Tai6.3107-R | GCTTGTTCCTCTTGCTCCTGT |
| Tai6.42353-F | CCGTATCCGAGTGAAGCAGA |
| Tai6.42353-R | GTTTCCACAGCCTTACCCTAG |
| Tai6.22648-F | CCGTATCCGAGTGAAGCAGA |
| Tai6.22648-R | GTTTCCACAGCCTTACCCTAG |
| Tai6.25300-F | AACCTCAACCCATCGATTTCTAC |
| Tai6.25300-R | GCCGTTACCACCCCCAGG |
| UBI-F | TCGCCGACTACAACATCCAG |
| UBI-R | TTCCTCAGCCTCTGCACCTTT |
